# Landscape transcriptomics reveals ecological constraints on fish under anthropogenic coastal change

**DOI:** 10.1038/s41467-026-77751-2

**Published:** 2026-10-09

**Authors:** Emma Gairin, Saori Miura, Zoé Chamot, Camille A. Sautereau, Jann Zwahlen, Hiroki Takamiyagi, Yann Gibert, Marcela Herrera, Vincent Laudet

**Affiliations:** 1 Marine Eco-Evo-Devo Unit, Okinawa Institute of Science and Technology, Onna-son, Japan; 2 Sorbonne Université, CNRS, Biologie Intégrative des Organismes Marins, BIOM, Observatoire Océanologique, Banyuls-sur-Mer, France; 3 CNRS IRL 2028 “Eco-Evo-Devo of Coral Reef Fish Life Cycle” (EARLY), Okinawa Institute of Science and Technology, Onna-son, Japan; 4 Department of Medical and Molecular Genetics, Indiana University, Indianapolis, IN USA; 5 Marine Research Station, Institute of Cellular and Organismic Biology (ICOB), Academia Sinica, Yilan, Taiwan

**Keywords:** Ecological genetics, Environmental impact, Molecular ecology, Marine biology

## Abstract

Urban zones are rapidly expanding worldwide, exposing organisms to complex and multidimensional stressors. However, linking organismal responses to environmental change at ecologically relevant scales remains a major challenge. To investigate the constraints imposed on fish living in coastal urban environments, we apply a landscape-scale framework to identify molecular signatures associated with human activities. Using transcriptomes from a common coral reef fish, *Chrysiptera cyanea*, sampled across 18 coastal sites in Okinawa, Japan, we implement scalable analytical and visualisation tools and identify coordinated shifts in metabolic, immune, and inflammatory pathways. By integrating field data with a fasting-feeding experiment, we find that transcriptomes of adult fish from urbanised sites resemble those of well-fed experimental fish, suggesting that these areas provide an energy-rich diet while imposing chronic stress. Our findings reveal how anthropogenic activities reshape organismal resilience and demonstrate the power of landscape transcriptomics as a link between molecular signatures and ecological outcomes across changing environments.

## Abstract (web summary)

Human activities are transforming coastal ecosystems, but their effects on wild animals remain difficult to measure. Here authors use landscape transcriptomics to reveal how urbanisation impacts metabolism, immunity and inflammation in reef fish.

## Introduction

Human activities are altering natural habitats worldwide, creating novel types of ecosystems exposed to complex combinations of stressors^1,2^. In the marine realm, a large portion of coastlines have become anthropised. Increasing numbers of organisms are exposed to habitat modification and stress from industrial, agricultural, and residential activities^3^. However, the impact of urbanisation on coastal organisms remains poorly understood^4,5^. Ecological monitoring commonly relies on parallel strategies: tracking changes in environmental parameters, such as water quality or biological communities^6–8^, and exposing model organisms to a single or few stressors under controlled laboratory conditions^9,10^. While these approaches provide valuable insights, they struggle to convey the impacts of the cocktail of pressures encountered by wild organisms in anthropised habitats^11^.

At the forefront of the response to environmental change, shifts in gene expression finely modulate biological processes in organisms^12^. In aquatic ecosystems, fish are a common study taxon^12,13^, and their physiology is well-characterised in model species such as zebrafish^14–17^. Extensive genomic resources and homology across teleost genes enable detailed molecular studies even in non-model species^18,19^. With the rise of high-throughput sequencing, transcriptomics has become a central approach for ecological and ecotoxicological studies in the laboratory but also in the field^13,20,21^. Unlike targeted methods, transcriptomics provides unbiased and hypothesis-free gene expression data^11,22–24^. Importantly, changes in gene expression can precede physiological effects, offering mechanistic insight and serving as early warning signals for later-stage outcomes such as disease, reduced fitness, or population declines^20,24^. Moreover, by focusing on conserved genes and pathways, transcriptomic responses can be compared across taxa, facilitating the development of generalisable frameworks for understanding organismal resilience under anthropogenic stress^19^. Building on these advantages, landscape transcriptomics—the study of how genome-wide expression patterns reflect dynamic, landscape-scale environmental drivers—has emerged as a comprehensive approach to monitor environmental change in wild organisms^11,12^. However, field-based transcriptomic studies remain scarce and their capacity to reveal ecologically meaningful patterns is yet to be fully validated.

To explore how urbanised environments reconfigure gene expression in coastal fish, we conduct a large-scale study centred on the blue damselfish *Chrysiptera cyanea*, a territorial reef species inhabiting diverse shallow coastal habitats of Okinawa, Japan^25^. We hypothesise that juvenile and adult *C. cyanea* integrate multiple and interacting environmental factors into coordinated shifts in gene expression, and that these can reveal physiological trade-offs involved in urban life that remain invisible to classical ecological monitoring. Specifically, we predict that human activities lead to a signature of chronic stress and altered energy metabolism, a pattern reported in terrestrial urban wildlife^26–28^.

In the field, gene expression is influenced by numerous factors, and these can have non-linear effects; sampling across stressor gradients rather than categorical factors is increasingly advocated for^29–31^. However, explicitly accounting for all factors affecting organisms would lead to a combinatorial explosion^9,32^. To address this, we adopt a semi-randomised and multifactorial sampling strategy^32–34^: we follow a gradient of urbanisation while capturing natural variability—including sampling time, temperature, salinity, fish size, and reproductive status. At each site, we collected five juveniles of varying sizes and four to five adult females or males. We use whole-body transcriptomes of juveniles to assess organism-wide responses, and target adult liver transcriptomes to evaluate longer-term adjustments of metabolism and detoxification.

An obstacle for ecological applications of transcriptomics is that statistical modelling of complex and context-dependent effects and interactions between factors requires careful design^29,35,36^, can suffer from overparameterisation^37,38^, and risks downplaying regulatory interactions between genes themselves^39,40^. Although analytical frameworks exist^40–43^, few simultaneously account for non-linear multi-stressor gradients and maintain sufficient transparency to identify meaningful patterns in field transcriptomes. To detect the footprint of human activities on juvenile and adult damselfish and focus on biological interpretability, we combine dimensional reduction, linear and quadratic modelling, and novel visualisation approaches. In particular, we developed a scalable framework to diagnose organismal status across environmental gradients by summarising transcriptomic data with radar plots^44^. In parallel, we experimentally manipulated food availability to obtain reference transcriptomic signatures linked to nutritional intake, facilitating the interpretation of feeding status in wild fish. Overall, instead of compartmentalising the effect of individual stressors, we aim to diagnose the status of fish in heterogeneous environments and to test whether classical biomarkers reflect human presence in coastal fish. Together, our approaches position landscape transcriptomes as robust resources to assess ecosystem condition and capture biological constraints associated with real-world settings.

## Results

### Environmental conditions drive coordinated molecular phenotypes in a coral reef fish

**Fig. 1:**
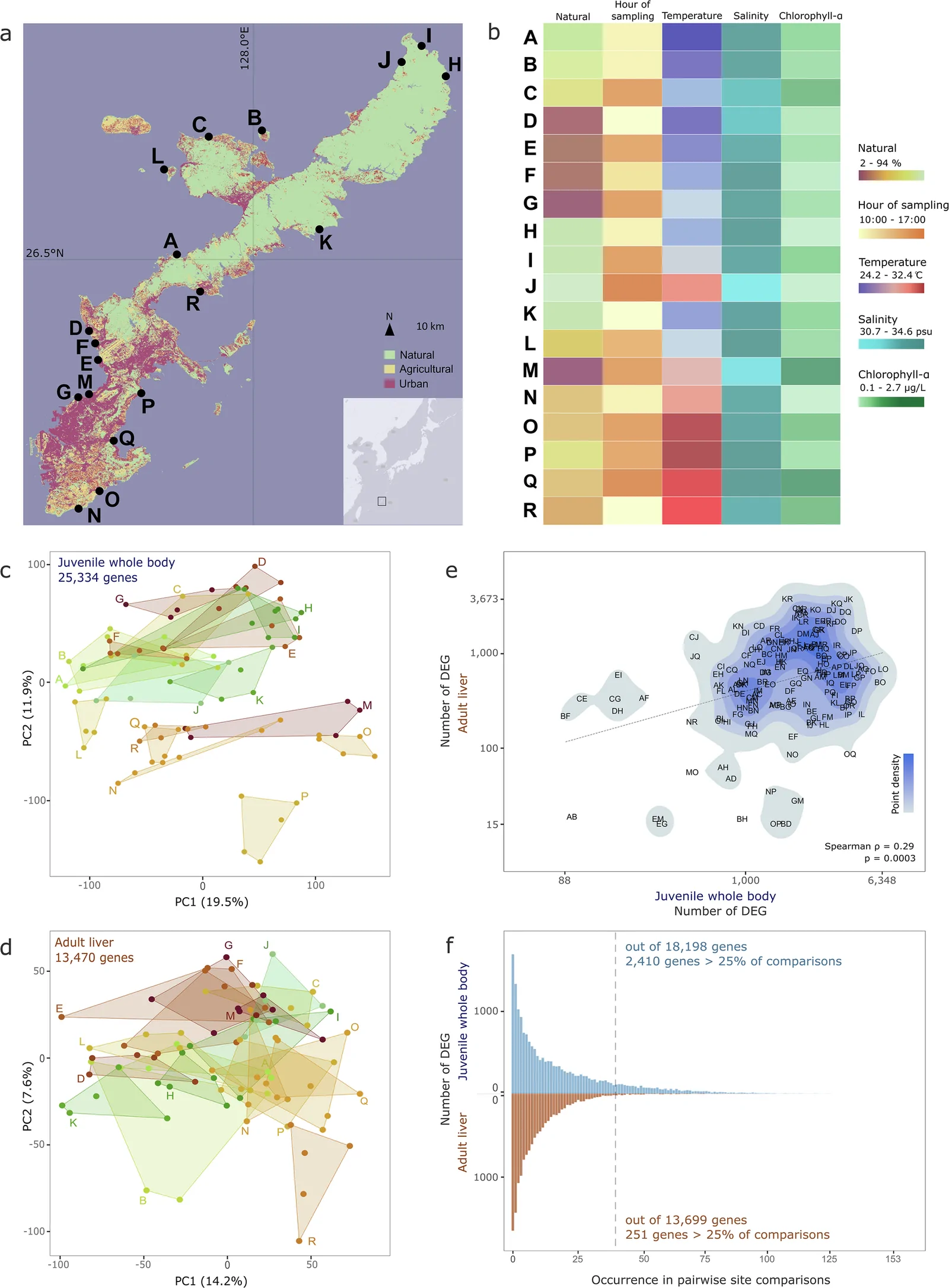
Environmental context and overview of the transcriptomic profiles from *Chrysiptera cyanea* juvenile whole-body and adult liver samples across 18 coastal sites in Okinawa, Japan. **a** Map of the eighteen field sites in Okinawa, overlaid on land-use data from JAXA (2023). Bottom right insert provides the geographical location of Okinawa. **b** Environmental parameters of each site: percentage of natural zones within a kilometre of each site, sampling time, temperature, salinity, and chlorophyll-α at each site (see full dataset in Supplementary Table 1). **c** Principal Component Analysis of the expression level of all genes from the transcriptomes of juvenile whole-body samples (*n* = 5 per site). Red-green colour-coding based on the proportion of natural zones within a kilometre of the site. **d** Principal Component Analysis of the expression level of all genes from the transcriptomes of adult liver samples (*n* = 4-5 per site), excluding 3,973 genes significantly differing in expression between males and females. Red-green colour-coding based on the proportion of natural zones within a kilometre of the site. **e** Number of differentially expressed genes (logarithm-10 scale) in juvenile whole-body and adult liver samples resulting from pairwise comparisons of 18 sites (absolute fold-change >1.5, *p* < 0.01). The density of points (one point per pairwise comparison) is represented in gradual grey-blue shading. f, Number of pairwise site comparisons where a differentially expressed gene occurs (as *n* = 18 sites were compared, a gene with a unique signature in all sites would occur in n(n-1)/2 = 153 pairwise site comparisons). A vertical dashed line corresponding to 39 pairwise comparisons (i.e. 25% of all comparisons) is used to illustrate the high site specificity of gene signatures in adult livers (251 genes in more than 39 pairwise comparisons) compared to whole-body juveniles (2410 genes).

To determine whether coastal habitats influence gene expression, we characterised the transcriptomic profiles of juvenile whole-body and adult liver samples of *C. cyanea* across 18 sites. The distribution of this blue damselfish species across varied environmental conditions on the subtropical island of Okinawa, Japan, allowed us to span a gradient from relatively pristine northern reefs to heavily urbanised southern coastlines (Fig. 1a,b).

We first checked that transcriptomic profiles were not confounded by genomic variation; variant calling using the juvenile whole-body transcriptomes retrieved no significant population structure across Okinawa (Supplementary Fig. 1). Dimensional reduction with Principal Component Analysis (PCA) highlighted that, although transcriptomic profiles were modulated by fish size or sex (Supplementary Fig. 2), samples from the same sites clustered closely (significant effect of site on PC1/2, PERMANOVA, F(17, 72) = 13.51, R^2^ = 0.76, *p* = 0.001, for juvenile whole-body; F(17, 71) = 8.57, R^2^ = 0.67, *p* = 0.001, for adult livers). This suggested a coherent and site-associated transcriptomic signal rich in information (Fig. 1c,d). Local environmental conditions exerted a consistent influence on molecular phenotypes across life stages: the number of differentially expressed genes in juveniles and adults for the same site pairs was correlated (Fig. 1e). Juveniles exhibited broad, frequently differentially expressed genes, with 2410 genes identified in >25% of the pairwise site comparisons (significantly exceeding null distribution, two-sample Kolmogorov-Smirnov test, D = 0.537, *p* < 10^-15^; Fig. 1f, Supplementary Fig. 3). While adults showed more site-specific, fine-tuned patterns in their liver, a key organ for energy metabolism and detoxification, 251 genes were nonetheless identified in >25% of comparisons (also significantly higher than null distribution, *D* = 0.52, *p* < 10^-15^). These developmental stage-specific patterns suggest a transition from early transcriptional plasticity in juveniles to more specialised, long-term molecular programs in adults.

### Radar plots reveal multidimensional transcriptomic signatures of local conditions

**Fig. 2:**
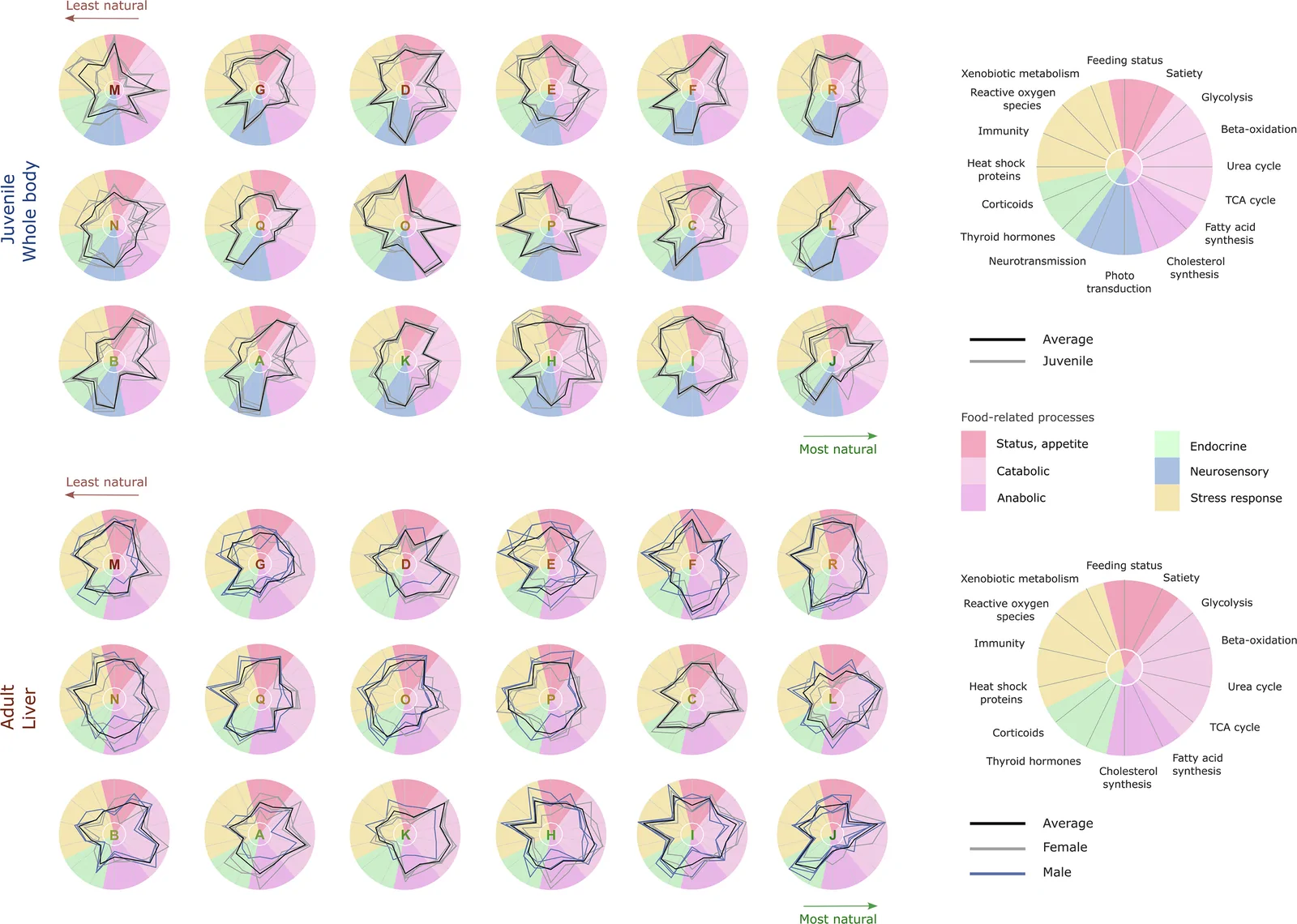
Radar plots summarising biological process-level profiles of *Chrysiptera cyanea* juvenile whole-body and adult liver transcriptomes across energy metabolism, endocrine regulation, neurosensory, and stress response. One radar plot per site (*n* = 18) and per sample type (juvenile whole-body or adult liver, *n* = 4-5) is presented. Radar plots are ordered by increasing percentage of natural zones within 1 km of the site, as reflected by the red (less natural)-green (more natural) colour coding of the site name at the centre of each radar plot. Black lines represent the average values of the samples for each site; individual variability is indicated in grey (juveniles, females) or blue (males). For each biological process, the top PC dimensions representing at least 50% of the variance in gene expression were retained. The first principal component of the average coordinates from each site across the retained PC dimensions was used to define a main axis of site-wise variance, summarising the dominant gradient of site-wise variation for each biological process. Each sample was projected onto this axis in multidimensional PC space. The projected values were min-max normalised (0 to 1). Values close to 0 reflect lower scaled gene expression for that process (cf. Supplementary Table 3 for correlations). Biological processes considered were: satiety signalling, glycolysis, beta-oxidation, ornithine-urea cycle, tricarboxylic acid (TCA) cycle, fatty acid synthesis, cholesterol synthesis, phototransduction (juvenile only), neurotransmission (juvenile only), thyroid hormone signalling pathway, hypothalamic-pituitary-interrenal (HPI) axis and steroidogenesis (corticoids), heat shock proteins, immunity, reactive oxygen species, and xenobiotic metabolism. Sample-specific feeding status (derived from Fig. 3) is indicated for context.

Landscape transcriptomes capture integrated organismal responses to local conditions, but the high dimensionality of these datasets—spanning functional gene groups, multiple sites, and environmental drivers—can complicate ecological interpretation. To address this, we developed a summarisation framework and leveraged curated gene sets for energy metabolism, neurotransmission, endocrine regulation, and stress response^19^, which are processes expected to vary in *C. cyanea* in response to human activities^26,27^. Building on dimensional reduction approaches^44^, we summarised the expression profile of each gene set to a single value representative of each sample compared to all others (cf. methods, Supplementary Fig. 4). Lastly, we visualised these values on radar plots (Fig. 2). Unlike heatmaps or PCAs, these simultaneously display the status of individual fish in terms of distinct biological processes, enabling the detection of co-occurring mechanisms and physiological trade-offs across sites and life stages.

Consistent radar profiles among fish from similar environments underscored the robustness of this approach, with intra-site variability reflecting size or sex differences. Juveniles from sites with comparable water temperature and urbanisation level (A, B, L, as well as N, R) exhibited closely aligned profiles, as did adults from the most natural sites (H, I) and most urbanised sites (E, G, M; see Fig. 2, Supplementary Fig. 5a,b). Genes related to a single, strong environmental driver directly relayed local characteristics, such as heat-shock markers in fish exposed to >31.9 °C in two sites (O and P), and the bimodal phototransduction signature in juveniles depending on morning or afternoon sample collection. These examples underscore the capacity of radar plots to extract patterns of interest and obtain diagnoses about the status of organisms in response to local conditions.

Transcriptomes are complex datasets, mixing genes that can be snapshots of stress with those conveying messages about long-term conditions. The comparison of juvenile whole-body and adult liver profiles allowed us to discern timescales at which environmental factors affect gene expression. For instance, on the two most urban sites (G and M), adult liver transcriptomes showed similar radar profiles, whereas juveniles had marked differences in corticoids, neurotransmission, and energy metabolism (also see respective PCAs on Fig. 1c,d). We hypothesise that juveniles from site G benefited from its more open reef structure, with frequent water renewal removing waterborne stressors. In contrast, adults could have integrated chronic stress through prolonged exposure to local conditions, particularly in terms of immune response and neurotransmission. Landscape transcriptomes can thus reveal time-integrated differences across environments that may be deemed similar, thereby making the ‘invisible’ visible.

### Integrating experimental and field transcriptomic data uncovers nutritional constraints

**Fig. 3:**
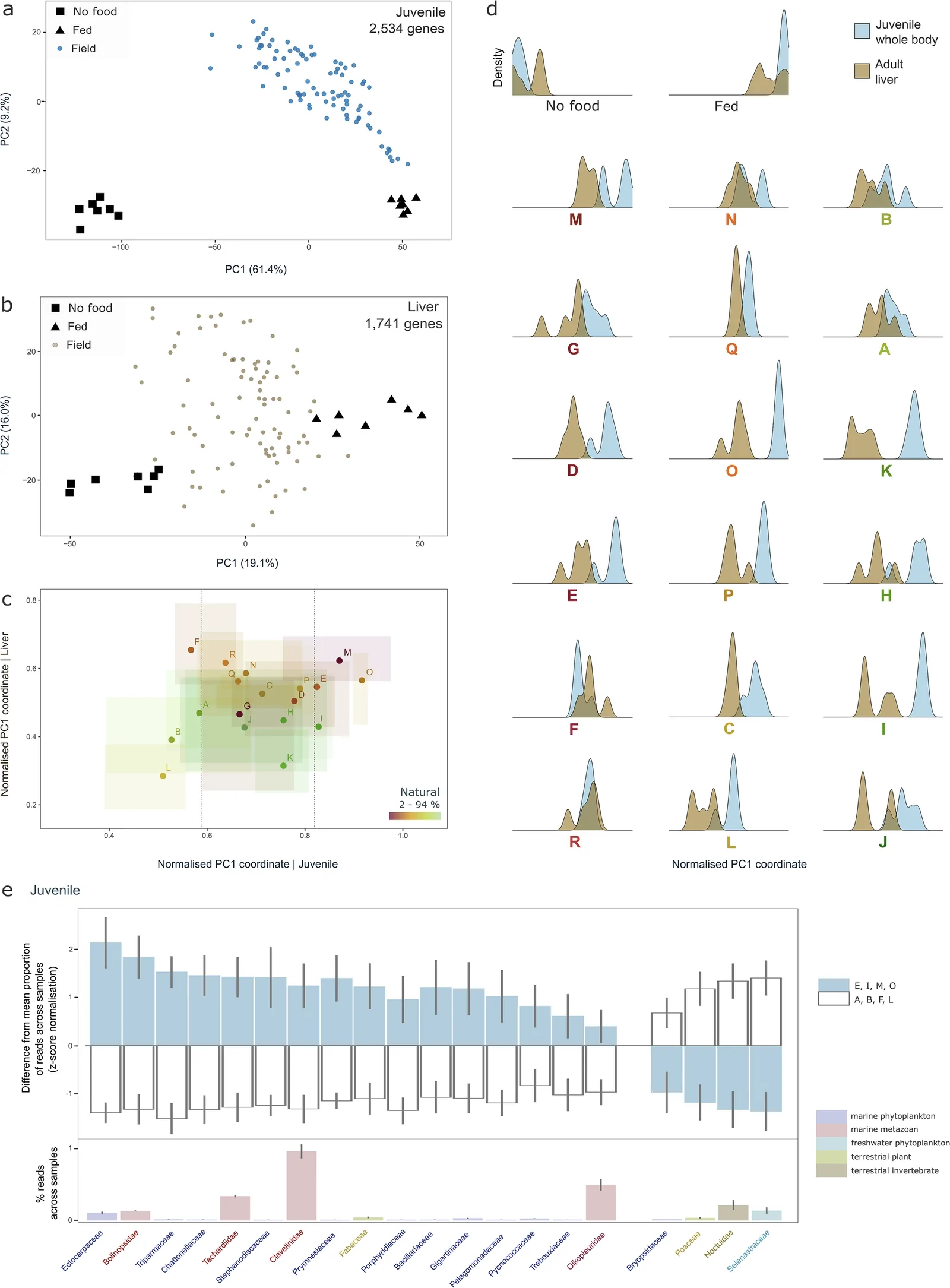
Derivation of feeding status in wild juvenile and adult *Chrysiptera cyanea* through an integration with experimental fasting-feeding transcriptomes. **a** PCA of 2534 genes in juvenile whole-body samples from field and fasting-feeding laboratory experiments. These genes represent the top 10% contributors to the PC1 variance of a preliminary PCA using experimental samples only (Supplementary Fig. 6d). Field samples (blue dots) are projected between fasted (black square) and fed (black triangles) experimental samples. **b** PCA of 1741 genes in adult liver samples from field and fasting-feeding laboratory experiments. These genes represent the top 10% contributors to the PC1 variance of a preliminary PCA using experimental samples only (Supplementary Fig. 6e). Field samples (brown dots) are projected between fasted (black square) and fed (black triangles) experimental samples. **c** Mean normalised PC1 coordinates of field samples (dots) and site-wise minimum-maximum ranges (rectangles). Colour coding corresponds to the percentage of natural zones within a kilometre of each site. Vertical dashed lines delimitate the four sites with lowest and highest PC1 values for juveniles (used in e). **d** Density spectra illustrating the variability in feeding status per site; sites presented based on the percentage of natural zones within a kilometre. **e** Validation of juvenile feeding status by metatranscriptomics. Proportion of taxonomically assigned non-host reads show significant deviations from the mean prevalence in sites with the highest (E, I, M, O) and lowest (A, B, F, L) predicted feeding status.

Radar plots revealed extensive variation in energy metabolism across the sites; to mechanistically link these phenotypes with controlled reference statuses, we conducted fasting-feeding experiments in juveniles and adults. Juveniles and adults were fasted or fed normally for 3 and 14 days, respectively, durations tailored to their physiological tolerance. Both life stages showed a clear separation between the transcriptomes of fasted and fed fish (cf. PCAs from Supplementary Fig. 6d,e). Genes driving this separation (top 10% of PC1 loadings) were enriched in processes central to energy balance, including cell cycle and division, DNA transcription, protein modification, lipid and carbohydrate metabolism, and glucose homeostasis, highlighting canonical pathways responsive to nutrition (Supplementary Fig. 7). Using these genes, we generated PCAs grouping the experimental and field samples (Fig. 3a,b), from which we extracted the feeding status of each fish using its PC1 coordinate as a proxy.

Strikingly, adults sampled near urban zones showed higher feeding status (linear mixed-effect model with site as a random effect, β = −0.066, 95% CI = [−0.105, −0.027], t(17.94) = − 3.56, *p* = 0.0023; Fig. 3c,d). In line with this, among diet-related genes with a modified expression in livers from urban zones, we found Insulin Receptor b *insrb* and Peroxisome Proliferator Activated Receptor Gamma Coactivator 1 Alpha *ppargc1a*, markers of insulin resistance and energy surplus.

Juveniles, in contrast, showed no significant association to the measured environmental parameters. However, 46 pairs of sites had significantly different PC1 coordinates, suggesting that site specificities did influence juvenile feeding status (Kruskal-Wallis χ^2^(17) = 65.7, *p* < 10^-6^ followed by Dunn’s post hoc test with Benjamini-Hochberg correction to identify significant pairwise differences, *p* < 0.05). To validate our PC1-based feeding proxy, we examined the taxonomic composition of non-host reads captured in juvenile whole-body transcriptomes. Sites where juveniles were predicted to be well fed showed higher representation of tunicates (*Clavelinidae, Oikopleuridae*), ctenophores (*Bolinopsidae*), brown algae (*Ectocarpaceae*), and red algae (*Gigartinaceae*; Fig. 3e). This qualitative signal reinforces the PC1 interpretation, illustrating that dietary parameters not easily measured in the field can influence transcriptomic signatures, and underscoring the value of complementary field and laboratory approaches.

Lastly, the mean PC1 coordinate was generally greater (higher feeding status) for juveniles than adults (Fig. 3d); the fasted treatment could have had a stronger impact on the juveniles over three days than on the adults over two weeks, leading to a more extreme response in the fasted juvenile group. Alternatively, Okinawa’s coastal zones may provide more adequate food to juveniles than to adults.

The feeding status of the fish was a lens to inform the analysis of the radar plots (Fig. 2) and enabled the detection of distinct strategies depending on developmental stage, environmental conditions, and food availability. Well-fed juveniles showed altered appetite-related radar signatures, reflecting a downregulation of satiety-signalling gene expression which can boost food intake and thus growth (Supplementary Fig. 8). Energy metabolism shifted from catabolic to anabolic processes with increasing feeding status: juveniles and adults with low-food status privileged glycolysis (e.g., juveniles from A, B, F, L; adults from A, B, C, H, I, J; Figs. 2,3); in some sites, β-oxidation, which can use fatty reserves to maintain energy homeostasis, was also upregulated (juveniles from B, L; adults from C, H, I, J). Inversely, well-fed fish showed high fatty acid and cholesterol synthesis (highest in O in juveniles, also in D, E, H, I, K, M, N, P; for adults, in F, N, R; Figs. 2,3). Overall, these results highlight that landscape transcriptome data provide a rich framework for linking experimental animal models with ecologically relevant physiological regulations and refining interpretations, here in terms of energy allocation strategies.

### Canonical pollution biomarkers do not capture human presence

As a last step in our assessment of environmental and human impacts on fish transcriptomes, we focused on common marker genes of pollution, including endocrine processes, xenobiotic metabolism (involved in the removal of chemical pollutants), neurotransmission, and immunity. These processes were expected to reflect the footprint of human activities; however, we did not detect clear associations of these processes with urbanisation (Fig. 2). While some links were found between genes involved in the immune response and human presence, most markers responded to other drivers (Supplementary Fig. 5c,d).

Numerous genes involved in endocrine signalling showed changes in expression levels correlated with temperature in both juvenile and adult transcriptomes, such as nuclear thyroid hormone receptors, consistent with previous observations in coral reef fish^45^. Low feeding status also influenced endocrine regulation in juveniles, notably through the upregulation of the expression of deiodinase *dio3b*, which can reduce thyroid-mediated gene transcription, and genes involved in the neuroendocrine cascade leading to cortisol release (*CRHr, pomc, fdx*, Supplementary Figs. 9, 10). Interestingly, *hsd11b1*, involved in the conversion of inactive cortisone to active cortisol, was upregulated in juveniles from the natural sites (H and I), suggesting an acute stress response linked to the strain of capture, potentially dampened by chronic stress elsewhere.

We also found feeding status, rather than urbanisation, to be linked to xenobiotic metabolism at both stages (Fig. 2, Supplementary Fig. 5c,d). This could be because many genes involved in the modification, conjugation, and excretion of compounds have a pleiotropic role and intervene in endogenous processes dependent on food-derived energy. Lastly, neurotransmission and immune defence mechanisms, which are energetically costly^46,47^, were suppressed under low food conditions in juveniles, even in urbanised environments (Fig. 2, Supplementary Fig. 5c,d); energetic constraints may override pollution-related signals.

### Identifying robust markers of human presence through sampling heterogeneity

By combining laboratory experiments with biological process and pathway-level visual summarisations, we uncovered subtle regulatory adjustments bridging local environmental conditions and physiological responses. In parallel, we identified strong effects of non-target factors—especially feeding status and temperature—on canonical biomarkers of pollution and stress (Fig. 2). This highlighted the need for context- and species-specific indicators.

**Fig. 4:**
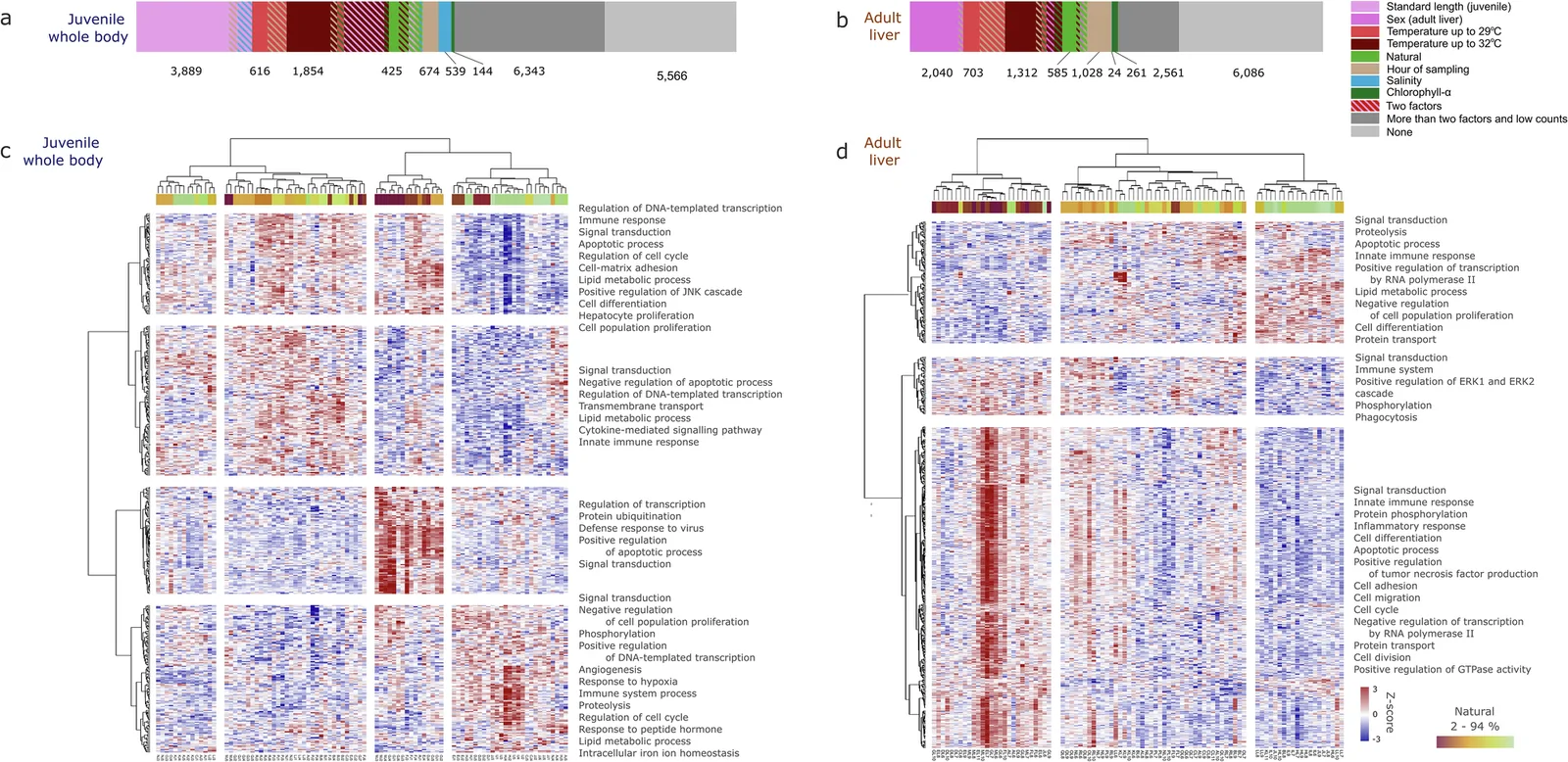
Disentangling the influence of environmental drivers and fish characteristics on gene expression in *Chrysiptera cyanea* juvenile whole-body and adult liver transcriptomes. **a** For juvenile whole-body transcriptomes, the bar plot indicates the number of genes with significant linear or quadratic associations with fish standard length or environmental parameters. Plain colours represent genes associated with a single parameter and hashed lines indicate genes associated with two factors. **b** For adult liver transcriptomes, the bar plot indicates the number of genes with significant differences, linear or quadratic associations with fish sex or environmental parameters. Plain colours represent genes associated with a single parameter and hashed lines indicate genes associated with two factors. **c** Heatmap of the 425 genes in juvenile whole-body transcriptomes showing significant associations exclusively with the percentage of natural zones within one kilometre of the site. Gene Ontology biological processes for the 4 main gene clusters, listed in order of prevalence within each cluster. **d** Heatmap of the 585 genes in adult liver transcriptomes showing significant associations exclusively with the percentage of natural zones within one kilometre of the site. Gene Ontology biological processes for the 3 main gene clusters, listed in order of prevalence within each cluster.

To obtain a gene-level view of the processes directly impacted by urbanisation in Okinawa’s *C. cyanea*, we leveraged our semi-randomised sampling approach and modelled gene expression against varied environmental factors (temperature, salinity, chlorophyll-α, sampling time) and fish traits (juveniles: size, adults: sex). Remarkably, more than 78% of the genes in juvenile whole-body transcriptomes and over 65% in adult livers were significantly associated linearly or quadratically with at least one parameter (Fig. 4a,b). The proportions of genes responding to each factor were significantly similar in juvenile whole-body and adult liver samples, suggesting strikingly parallel molecular programs across tissues and developmental stages (Pearson’s Chi-squared test, χ^2^(19) = 3548, *p* < 10^-15^). Temperature emerged as the strongest single driver, with 1,854 genes in juvenile whole-body and 1312 genes in adult livers responding exclusively to variations in temperature. Excluding the four warmest sites (29.6–32.4 °C) reduced the number of temperature-associated genes by almost two-thirds, underscoring a disproportionate influence of intense thermal stress (Fig. 4a,b). Sampling time (circadian effects), fish size, and sex also had important footprints on gene expression.

We identified 425 genes in juvenile whole-body and 585 genes in adult livers associated with human activities and no other tested parameter (list of genes provided on FigShare; for robustness testing, cf. Supplementary Fig. 11). These conveyed generalised stress with modifications in the expression level of genes involved in apoptosis, immunity, and signal transduction (genes such as interleukins, chemokines, caspases; processes including antigen processing and presentation, virus infection, T cell, toll-like, and NOD-like receptor signalling, chemokine and NF-kappa B signalling). Expression of genes involved in cell differentiation, tissue homeostasis, protein transport, proteolysis, and transcription decreased near urbanised sites, while genes linked to inflammation, carcinogenesis, reactive oxygen species, protein phosphorylation, and cell cycle regulation were upregulated (Fig. 4c,d). Because these genes did not respond linearly or quadratically to other environmental or organismal factors, they may represent diagnostic markers of human-related stress, conveying mechanisms used by fish to survive in urban environments.

## Discussion

While urbanisation effects on wild organisms at the population level are increasingly reported^48^, integrated responses to complex multi-stressor conditions remain largely unresolved. Here, landscape transcriptomics provides a molecular-based perspective on how anthropogenic pressures shape organismal status in urbanised coastal ecosystems. We observed that adult coral reef fish from Okinawa living on coastal sites close to human activities were well-fed, but showed elevated immune and inflammatory responses (Figs. 3,4d). This suggests that fish survival in urban zones depends on a trade-off: despite local stressors, increased food availability potentially attracts fish and could fuel stress responses^49,50^. Similar patterns have been reported in other field-based transcriptomic studies: in two bird species, immune response was upregulated and coupled with a lipid-rich diet in urban zones^26,27^. In contrast, immune and stress responses in juvenile fish were coupled with nutrition rather than with human presence, a tight link that persisted even in individuals from relatively pristine zones (Fig. 2, Supplementary Fig. 5c,d). This footprint of food intake on stress markers was also noted in the Elbe estuary, where juvenile fish immune response at the transcriptomic level was hypothesised to be suppressed under starvation^51^. Crosstalk between nutritional status and stress mechanisms may thus be conserved across taxa and life stages, with energy availability influencing responses to anthropogenic effects.

Disentangling these interacting factors required moving from single markers to an integrated genome-wide perspective across variable field conditions. At the molecular level, the comparison of gene expression profiles in organisms from multiple environments defined a composite baseline signature^12^, with deviations reflecting the effect of local parameters on biological processes^52^ (cf. Figure 2). Sampling *C. cyanea* across a large range of environmental contexts revealed intricate patterns of gene expression depending on local conditions, developmental stage, tissue, sex and size (Fig. 4a,b). The absence of clear human-related signal from most ‘classic’ pollution markers echoed results from other field-based approaches in urbanised systems^49^. This has important implications for environmental monitoring, as qPCR-based approaches based on genes selected a priori may capture confounding factors, such as food availability or temperature (Fig. 4a,b, Supplementary Fig. 5c,d).

The gene-environment atlas derived from *C. cyanea’*s landscape transcriptomes in Okinawa highlighted complex relationships and interacting effects between environmental drivers, and identified genes exclusively associated with human presence in juvenile whole-body and adult liver transcriptomes. These constitute reference resources to select genes for targeted molecular assays while excluding non-target effects. Adopting this methodology across field contexts would be of high value, as transcriptomic signatures provide higher specificity and sensitivity than single traits or markers under multi-stressor conditions^53^ and can be used to disentangle compensatory effects from irreversible consequences^13,49^. Our results support a transition in environmental monitoring from discrete measurements of local parameters and organismal traits to the precise detection of organismal stress, even at sublethal and low intensity levels^54,55^, and prediction of longer-term outcomes at the population level^13,20,56–58^.

Interpreting field-derived datasets requires comparing transcriptomic patterns to environmental parameters and laboratory evidence. However, bridging controlled experiments and heterogeneous field conditions remains a central challenge in environmental toxicology^21^. Our results show that laboratory-based studies can inform landscape transcriptomics, particularly for parameters that are difficult to measure in situ. Using a PC1-based feeding proxy, we observed that juvenile diet correlated with transcriptomic signatures (Fig. 3) and highlighted how feeding status influences gene expression patterns across different habitats. To enhance data interpretation, the footprint of different stressors from additional laboratory assays^59^ (e.g., for temperature, salinity, prevalent chemical compounds) or from publicly available databases (based on gene homology between related species) can be key resources. The latter approach has been adopted in human toxicology and medical research^60^ and is increasingly advocated for in the fish community^56,61^. Cross-comparability of transcriptomes from various studies and experimental contexts, accompanied by robust analytical methods, promises to uncover novel effects between factors^29,59^. This opens the door to large-scale meta-analyses combining laboratory, mesocosm, and field data.

Landscape transcriptomics is an emerging frontier in ecological and ecotoxicological research and stands to transform how we conceptualise organism-environment interactions. Our study of localised gene expression responses in a common fish species in Okinawa is a step towards a framework for environmental state awareness, or ‘environmental phenotyping’, using transcriptomes. With sufficient genomic resources and environmental information for baseline analyses, our methodology can be extended to sentinel species across ecosystems. Global monitoring depends on scalable, mechanistic, and generalisable approaches; our results show that transcriptomic data can bridge molecular physiology with environmental change at landscape scales.

## Methods

### Study location

The subtropical island of Okinawa, Japan, is the largest island of the Ryukyu archipelago. It has an area of ~1200 km² and a population of 1.38 million^62^. Most inhabitants live in the southern half of the island. The average ocean temperature is 25 °C due to the influence of the warm Kuroshio current, and the island is surrounded by a fringing and patch reef system, which has a length of ~380 km. Coral communities represent an area of 69.8 km^2^^62^.

Okinawa is an ideal location for this study, with coastal ecosystems heavily influenced by nearby human activities and a range of contrasting coastal ecosystems along different zones, from dense urban areas near the city of Naha in the south, to the Yanbaru forest in the north. The water quality in Okinawa is affected by human activities. Rivers in urban zones were found to have high ammonium and chemical compound concentrations, notably of pharmaceuticals, with compounds such as bezafibrate, caffeine, clarithromycin, and sulfamethoxazole found at higher levels than the 90^th^ percentile across 109 major Japanese rivers^63^. Endocrine disruptors such as nonylphenol, Bisphenol A, and per- and polyfluoroalkyl substances have been measured in rivers and reefs, with concentrations related to the density of human activities nearby^64,65^. Agricultural zones of Okinawa are linked with nitrite, nitrate, ammonium, and red soil runoff^66^. Human presence is also associated with habitat degradation and coastal hardening to protect shores from erosion and reclaim fringing reefs^67,68^.

### Environmental data collection

Eighteen sites were visited from May to July 2023 along the coastline of Okinawa (Supplementary Tables 1,2), during the reproductive season, which is when juvenile *C. cyanea* can be collected. On each site, using a multiparameter probe (RINKO-Profiler CTD logger), water temperature, salinity, dissolved oxygen, turbidity, and chlorophyll-α were measured.

Three replicate 25 m long x 4 m wide transects were performed to record the fish community. These transects were positioned parallel to the shore, ten meters apart, in at least one meter of water. Transects were not conducted at two sites, Uka (J) and Kayo (K), due to limited water depth or strong currents. Two passes were performed per transect; mobile, more visible fishes were recorded during the first pass and more cryptic fishes during the second pass^69^. All fish were identified to the species level. The full dataset is available in FigShare. The average fish density (number of fish per m²) and species richness (number of species per m²) were calculated. Additionally, the substrate cover on each site was assessed across the three transects using a point-intercept method, with the type of substrate (live coral, dead coral, macroalgae, seagrass, rubble, hard substrate, sand) recorded every meter.

Land use near each site was quantified from a publicly available dataset released in January 2023^70^. As Okinawa’s coastal zones are characterised by karstic geology with high permeability and rapid subterranean water flow, coastal zones are influenced by localised groundwater and surface outfluxes rather than solely by surface topography-defined watersheds. Therefore, we used the percentage of natural zones within 1 km radial buffer around the coordinates of each field site as a proxy for human presence. Spatial analysis was performed in QGIS 3.14^71^ by extracting the percentage of pixels belonging to natural zones (evergreen broad-leaved forests, evergreen needle-leaved forests, mangrove forests) and excluding pixels corresponding to water bodies. This proximity-based metric served as a proxy for the degree of exposure to human activities near each site.

Non-Metric Multidimensional Scaling (R package *vegan* 2.5–7^72^) was used to assess the relationship between the sites based on the environmental parameters and on the juvenile and adult fish communities (Supplementary Fig. 12). There were no significant correlations between the percentage of natural zones near each site and any of the other considered environmental parameters.

### Study species

The blue damselfish *C. cyanea* is a coral reef damselfish characterised by its bright blue pigmentation. It occurs on shallow ( < 10 m) subtropical and tropical reefs of the Indo-Pacific and can most generally be found around rubble and corals in subtidal reef flats and tide pools. It is one of the most abundant fish along the coastline of the island of Okinawa, Japan, and can be found from urban river mouths to isolated beaches^25^. *C. cyanea* is territorial and lives in small to large schools that typically consist of a few males and several females^73,74^. The species displays protogynous hermaphroditism, with females turning into males. In Okinawa, during the reproductive season (typically May-August^75,76^), large numbers of juveniles can be found in clusters with or without adults on shallow coastal reefs.

### Fish collection and sample preparation

Field sampling was conducted in accordance with standard ecological protocols and with approval from the Okinawa Institute of Science and Technology (FWA-2022-008). All efforts were made to minimise animal distress during handling. On 18 sites along the coast of Okinawa, 5 juvenile and 5 adult *C. cyanea* (4 on site B) were collected using hand nets and snorkelling or SCUBA diving. In total, 90 juveniles, 49 females, and 40 males were collected and brought back to shore in transparent plastic bags allowing water flow. The handling time between sampling and euthanasia varied between 5 and 20 min. The fish were euthanised in a 200 mg/L Tricaine Methanesulfonate (MS222) solution following the guidelines for animal use issued by the Animal Resources Section of OIST Graduate University. Juvenile fish were photographed and their whole bodies were directly placed in RNAlater for tissue preservation. Adult fish were photographed, weighed, and dissected on-site. The livers were preserved using RNAlater. All RNAlater-preserved samples were kept on ice for transport, placed at 4 °C for 24 h, and stored at −20 °C until extraction.

The average size of the juveniles was 9.6 ± 0.7 mm SL [range of 7.9–12.0 mm SL]. There was no significant difference in juvenile fish standard length across sites. Juvenile fish were not weighed due to field logistics. For adult fish, the average size of the females was 34.5 ± 5.3 mm SL and of the males was 43.8 ± 4.6 mm SL [23.5–56.0 mm SL]. The average weight of the females was 1.9 ± 0.8 g and of the males was 3.6 ± 1.2 g [0.9–7.5 g]. The Fulton’s body condition factor (weight of the fish in grams divided by the cube of total length in cm) of the females was 1.73 ± 0.31 and of the males was 1.66 ± 0.23 [1.14–2.60]. There was no significant difference in Fulton’s body condition factor across sites for females and males. The characteristics of each fish from the field are listed in Supplementary Data 1 and 2.

### Feeding experiment

Ethical approval for the feeding experiment was granted by the Okinawa Institute of Science and Technology (ACUP-2022-388, 2023-014). The effect of food availability on the transcriptomic profile of *C. cyanea* was assessed with two experiments, for juveniles and adults. The experiment lasted 3 days in juveniles and 14 days in adults due to differences in tolerance to fasting between the distinct developmental stages.

For juvenile fish, juveniles collected in Apogama, Okinawa, Japan (26.50003 N, 127.84261 E) were kept at the OIST Marine Science Station. After one night of acclimation, they were placed in two tanks with 14 juvenile fish per tank. Water input came from pumped seawater. Two treatments were used: fasted and fed (control). In one tank, the fish were fed standard amounts of food for three days (dry pellets twice per day, ASIN: B0BFJ9MY8P); in the other tank, the fish were not fed for three days and a mesh filter was used on the water inlet to remove particles and plankton. After three days, each fish was weighed, measured, euthanised with MS222, and dissected. The whole body of each individual was preserved in RNAlater. Seven fish per treatment (14 samples in total) were used for RNA sequencing (Supplementary Data 3). The average size of the sequenced juveniles at the end of the experiment was 10.5 ± 0.8 mm SL for the fasted group and 12.3 ± 1.2 mm SL for the fed group. The average weight was 33 ± 9 mg for the fasted group and 77 ± 22 mg for the fed group. Fulton’s condition index was 1.36 ± 0.20 for the fasted group and 2.00 ± 0.21 for the fed group.

For adult fish, female *C. cyanea* collected in Sesoko, Okinawa, Japan (26.65056 N, 127.8549 E), were kept at the OIST Marine Science Station. After an acclimation period, they were placed in two tanks with eight fish per tank. Water input came from pumped seawater. Two treatments were used: fasted and fed (control). In one tank, the fish were fed standard amounts of food for two weeks (dry pellets twice per day, ASIN number: B0BFJ9MY8P); in the other tank, the fish were not fed for two weeks. The average weight of the fish at the start of the experiment was 2.3 ± 0.7 g for the fasted group and 2.3 ± 0.9 g for the fed group. The average weight of the fish after one week was 2.1 ± 0.8 g for the fasted group and 2.4 ± 1.1 g for the fed group. After two weeks, each fish was weighed, measured, euthanised with MS222, and dissected. Their livers were preserved in RNAlater. Of these samples, seven per treatment (14 in total) were used for RNA sequencing (Supplementary Data 4). The average size of the sequenced fish after two weeks, at the end of the experiment, was 40.9 ± 5.3 mm SL for the fasted group and 40.7 ± 4.6 mm SL for the fed group. The average weight was 2.0 ± 0.7 g for the fasted group and 2.4 ± 0.8 g for the fed group. Fulton’s condition index was 1.70 ± 0.09 for the fasted group and 2.04 ± 0.22 for the fed group. The Hepatosomatic index (100 x liver weight over the total weight of the fish) was 0.80 ± 0.14 for the fasted group and 1.55 ± 0.27 for the fed group.

### RNA extraction, library preparation, sequencing, and data processing

RNA extractions were performed using the Maxwell® RSC simplyRNA tissue kits and an automated Maxwell® RSC instrument based on the manufacturer’s recommendations (Promega, Cat. No. AS2000, Wisconsin, USA). The concentration and quality of RNA were assessed with an Invitrogen Qubit Flex benchtop fluorometer and an Agilent 4200 TapeStation. Quality check information is provided in Supplementary Data 5 and 6. Library preparation was performed using the NEBNext Ultra II Directional RNA library prep kit for Illumina (New England BioLabs, USA). Sequencing was done on an Illumina NovaSeq6000 platform at the OIST Sequencing section. Paired-end reads (2 × 150 bp) were obtained. The average number of paired-end reads was 72.2 ± 6.5 million for the juvenile and adult field samples, 68.0 ± 6.0 million for feeding experiment juvenile whole-body samples, and 80.9 ± 9.3 million for feeding experiment adult liver samples.

Quality control of the RNA sequencing data was performed with FastQC v0.12^77^. Following quality control, data were processed by trimming the adapters used by Illumina on each transcript as well as removing low-quality sections with Trimmomatic 0.39^78^ (ILLUMINACLIP:TruSeq3-PE-2.fa:2:30:10:8:keepBothReads LEADING:3 TRAILING:3 SLIDINGWINDOW:4:15 MINLEN:36 HEADCROP:11). To reduce transcript-level redundancy and focus on gene regulatory shifts, we used a reference transcriptome filtered to only keep the longest isoform for each of the 28,173 annotated genes. This reference transcriptome is available on FigShare (doi: 10.6084/m9.figshare.27143571)^79,80^. Gene-level abundance was quantified using Kallisto 0.46.2^81^. The number and percentage of pseudo-aligned reads were 48.0 ± 4.9 (mean ± sd) million reads and 68.1 ± 3.8 % for field juvenile whole-body samples, 58.5 ± 7.1 million reads and 81.7 ± 7.1 % for field adult liver samples, 39.6 ± 4.0 million reads and 58.5 ± 3.5 % for feeding experiment juvenile whole-body samples, and 58.6 ± 6.7 million reads and 73.3 ± 1.7 % for feeding experiment adult liver samples.

### RNA-seq data analysis

All analyses were performed on the transcriptomes of juvenile whole-body and adult liver samples separately. All data analysis and figure generation scripts are available on FigShare. R 4.3.3^82^ was used. Plots were generated using ggplot2 3.5.1^83^, ggtern 3.5.0^84^, ggradar 0.2^85^, pheatmap 1.0.13^86^. Principal Component Analyses (PCA) were obtained with the prcomp function from stats 4.3.3^82^. Statistical tests included non-parametric Kruskal-Wallis Rank Sum (stats 4.3.3^82^), Kolmogorov-Smirnov (stats 4.3.3^82^), and linear mixed effect models (lme4 1.1-38^87^) with environmental variables, fish size or sex as fixed effects and site as a random effect. Model selection was based on dredging to minimise the AIC criterion using MuMIn 1.47.1^88^. GO terms (based on the zebrafish *Danio rerio* reference org.Dr.eg.db) were analysed semantically with GoSemSim 2.28.1^89^.

Using all field samples, gene counts were normalised with DESeq2 1.36.0^43^. Genes with more than 10 counts in at least two sites were kept. Following variance stabilising transformation (vst) with DESeq2 1.36.0^43^, the matrix of the gene expression level for each gene in the field samples was used as the basis for the analysis. A second vst-normalised matrix combining the field and feeding experiment samples together was also used, keeping genes with more than 10 normalised counts in at least two sites or feeding treatments.

As there are no reference or control conditions in the field, we focused on comparisons between each site. Using DESeq2 1.36.0^43^, the number of differentially expressed genes in pairwise site comparisons was estimated with thresholds of adjusted *p* < 0.01 and absolute fold change of 1.5 (logFC of 0.58). Since 18 sites were considered, 153 pairwise comparisons were performed. To evaluate whether genes were repeatedly differentially expressed (DE) across sites more frequently than expected by chance, we generated an empirical null distribution based on 1000 permutations where each pairwise site comparison column was independently shuffled without replacement, preserving the overall number of DEGs detected in each pairwise site comparison while randomising gene identity. For each permutation, gene recurrence counts were recalculated to establish the expected null distribution. We compared the empirical cumulative distribution of observed gene recurrences (Fig. 1e) against the permuted null distribution using a two-sample Kolmogorov–Smirnov test (ks.test in stats 4.3.3^82^). Tail significance was evaluated using empirical permutation *p*-values, defined as the proportion of the 1000 null iterations where the count of high-recurrence genes ( > 25% of comparisons, i.e., 39 comparisons) met or exceeded the observed count.

The genes responding to sex in the adult liver samples were derived using DESeq2 1.36.0^43^ with the same significance thresholds. Using DRomics 2.6-2^42^, the genes responding linearly or quadratically to various environmental factors (percentage of natural zones near each site, temperature, sampling time, salinity, chlorophyll-α) and to size (for juvenile fish) were obtained at a significance threshold of adjusted *p* < 0.01. We grouped together genes that have a linear and quadratic response to focus on which genes are sensitive to given parameters in a monotonic or biphasic manner. This methodology is based on comparing the expression level of the genes in the samples to each parameter separately and thus does not account for complex interactive effects between parameters. However, current transcriptome-wide modelling frameworks do not support exhaustive model selection across multiple interacting factors, and thus we elected to use DRomics 2.6-2^42^ to test for linear and quadratic effects with a robust statistical approach. We focused on the genes that responded uniquely to the percentage of natural zones near each site, and to no other parameter used in DESeq2 1.36.0^43^ (female vs. male effect in adult livers) or DRomics 2.6-2^42^ (all other parameters). For these genes, as an additional layer of verification, sensitivity analysis was performed by gradually excluding 1 to 6 sites from the models and determining whether the genes were still retrieved and thus showed robust association with the percentage of natural zones near each site. Two categories of genes emerged: those with robust association (low frequency of absence in models) and those dependent on the inclusion of particular sites (Supplementary Fig. 11).

An annotated genome is available for *C. cyanea* with gene names identified for 26,300 genes using BLAST^79^. Manually curated sets of genes corresponding to key biological processes were used (satiety, glycolysis, beta-oxidation, ornithine-urea cycle, tricarboxylic acid (TCA) cycle, fatty acid synthesis, cholesterol synthesis, phototransduction, neurotransmission, thyroid hormone signalling pathway, hypothalamic-pituitary-interrenal (HPI) axis and steroidogenesis, and reactive oxygen species)^19^. In addition, genes associated with immunity, heat shock proteins, and xenobiotic metabolism were identified for the analysis.

### Meta-transcriptomic analysis

To estimate diet in juvenile *C. cyanea*, Kaiju 1.10.0^90^ was used to taxonomically assign the juvenile whole-body reads that did not map to the reference *C. cyanea* transcriptome. These non-aligned reads were compared with 2462 reference NCBI protein sequences available for the following taxa: Artemia (NCBI ID: 6660), Chlorophyta (3041), Ctenophora (10197), Cyanobacteria (1117), Dinoflagellata (2864), Embrophyta (3193), Fungi (4751), Haptophyta (2830), Ochrophyta (2696291), Pancrustacea (197562), Rhodophyta (2763), Tunicata (7712). The proportion of reads in each sample corresponding to different taxonomic families was identified, and deviations from the mean were used to identify differences in diets across the sites.

### Feeding and field experiment analysis

Fasting-feeding experiments were used to derive the feeding status of the field fish. To do so, PCAs were performed on the expression level of all genes of the juvenile whole-body and adult livers following the feeding experiment. The top 10% of genes with highest loading along PC1 were identified. The expression level of these genes in both the feeding and field samples was used for secondary PCAs for the juvenile whole-body and adult liver samples. In these secondary PCAs, the field samples fell between the feeding samples along PC1. Thus, the PC1 coordinate of each sample was taken as an indicator or proxy of each sample’s feeding status. This yielded five datapoints per site and sample type (four for adult livers from site B).

### Radar plots

Radar plots were produced to summarise the gene expression profile of each sample for targeted biological processes. The methodology underlying the radar plots is based on the RadarOmics package^44^ (cf. sketch of the method, Supplementary Fig. 4). In detail, sets of key genes related to biological processes were identified—satiety, glycolysis, beta-oxidation, ornithine-urea cycle, TCA cycle, fatty acid synthesis, cholesterol synthesis, phototransduction, neurotransmission, thyroid hormone signalling pathway, HPI axis and steroidogenesis, heat shock proteins, immunity, reactive oxygen species, and xenobiotic metabolism—were used^19^. A Principal Component Analysis (PCA) was performed for each set of genes. The number of PC dimensions accounting for 50% or more of the variance was obtained; this threshold was used to capture a consistent proportion of the variance across the gene sets. Using these PC dimensions, the average coordinates of the samples from each site were calculated. The first principal component of the average site coordinates across the retained PC dimensions was used to define a projection axis representing the main axis of site-wise variance. Each sample was projected along this axis, yielding a segment bounded by the two most extreme samples. The projection of each sample along this axis was scaled to obtain one value per sample ranging from 0 to 1. The assignment of the values depended on the average expression in the two most extreme sites along the axis (sample with a value of 0 close to the most extreme site showing lower expression, and 1 close to the opposite most extreme site that had higher expression). For most biological processes screened to obtain the radar plots, there was a linear correlation between the calculated coordinate of each sample and the mean expression level of the genes within the set (Supplementary Table 3). The relationship between radar plot coordinates and gene expression was checked with heatmaps for biological processes of interest (Supplementary Figs. 8, 9, 10). The relationship between radar plot coordinates, environmental parameters, and intrinsic fish characteristics (standard length for juveniles, sex for adults) was tested using linear mixed-effect models with site as random effect (lme4 1.1–38^87^, Supplementary Fig. 5c,d). Similarity between sites was derived from Euclidean distances between radar plot coordinates (Supplementary Fig. 5a,b).

### Population genetics

For juvenile whole-body transcriptomes, in which a higher number of genes was expressed than in the adult liver samples and thus a larger proportion of the genome was captured, the raw sequencing reads were mapped to the reference genome using two-pass mapping using STAR 2.7.9a^91^ followed by data cleaning with Picard (AddOrReplaceReadGroups, MarkDuplicates). GATK 4.4.0.0^92^ was then used for variant calling following the pipeline from Genomics Core at NYU CGSB^93^. SNPs were filtered to exclude low-quality variants. Principal Component Analysis was performed using PLINK 1.9^94,95^, removing samples with over 25% of missing genotype data (11 samples out of 90) and SNPs with missing genotype rates higher than 25% (9,040 variants retained). Discriminant Analysis of Principal Components (DAPC) was performed in R using adegenet 2.1.10^96,97^. No significant population structure was found across five zones around the island – northwest, north, east, south, west (Supplementary Fig. 1).

### Reporting summary

Further information on research design is available in the Nature Portfolio Reporting Summary linked to this article.

## Supplementary information

Supplementary Information

Description of Additional Supplementary Files

Supplementary Data

Reporting Summary

Transparent Peer Review file

## Data Availability

Raw sequencing datasets generated in this study have been deposited in the NCBI BioProject repository under accession codes: PRJNA1168661 (field samples; https://www.ncbi.nlm.nih.gov/bioproject/PRJNA1168661/), PRJNA1271234 (juvenile feeding samples; https://www.ncbi.nlm.nih.gov/bioproject/PRJNA1271234/), PRJNA1168266 (adult feeding samples; https://www.ncbi.nlm.nih.gov/bioproject/PRJNA1168266/). Land use datasets are available from JAXA’s High-Resolution Land Use and Land Cover Map of Okinawa Island (2023) at https://www.eorc.jaxa.jp/ALOS/en/dataset/lulc/lulc_v2301_e.htm. All processed data and R scripts are publicly available on figshare, https://doi.org/10.6084/m9.figshare.27966972.

## Code availability

The files and R scripts generated in this study have been deposited in Figshare (https://doi.org/10.6084/m9.figshare.27966972).
