# Supplementary material for: Landscape transcriptomics reveals ecological constraints on fish under anthropogenic coastal change
Source: Nat Commun. 2026 Oct 9;17:10218. doi: 10.1038/s41467-026-77751-2 (PMC13652805; doi:10.1038/s41467-026-77751-2)
Supplement: Supplementary file 1 [file 41467_2026_77751_MOESM1_ESM.pdf]

Supplementary Table 1: Site name, coordinates, date and hour of sampling, percentage of urban, agricultural, and natural zones within a kilometre of each site, and environmental parameters (temperature, salinity, chlorophyll-α, turbidity, dissolved oxygen).

Site	Site name	Latitude (N)	Longitude (E)	Nearby features	Date	Hour of sampling	Urban (%)	Agricultural (%)	Natural (%)	Water temperature (ºC)	Salinity (psu)	Chlorophyll-α (µg/L)	Turbidity (FTU)	Dissolved Oxygen (%)
A	Seragaki	26.50514	127.8801	Village, forest	24/05/2023	11:00	21	10	69	24.2	34.5	0.3	0.7	101
B	Kouri	26.71363	128.0197	Agriculture	25/05/2023	11:00	5	39	56	24.7	34.4	0.2	0.8	107
C	Nakijin	26.70474	127.9302	Village, agriculture	25/05/2023	15:00	26	35	38	26.0	34.0	0.5	1.2	123
D	Toguchi	26.36946	127.7332	Village, agriculture, military installation	26/05/2023	10:00	22	70	8	24.6	33.9	0.2	0.1	102
E	Junkyard	26.33682	127.7444	City, industry, military installation	26/05/2023	14:30	42	45	13	25.1	34.3	0.2	0.2	103
F	Mizugama	26.36009	127.7398	City, large river	29/05/2023	12:00	61	25	14	25.7	34.5	0.1	0.1	120
G	Minatogawa	26.27478	127.7143	Industrial zone, military installation	29/05/2023	15:00	80	13	7	26.9	34.4	0.2	0.1	137
H	Adan	26.80111	128.3195	Forest	07/06/2023	11:00	3	11	86	25.8	34.5	0.1	0.0	110
I	Oku	26.85067	128.2821	Forest, pig farm	07/06/2023	15:00	1	13	86	27.1	34.4	0.3	0.8	148
J	Uka	26.83113	128.2459	Forest, main road	07/06/2023	17:00	5	0	94	28.6	30.7	0.1	0.0	162
K	Kayo	26.54939	128.1106	Forest, village	09/06/2023	10:00	3	14	83	25.5	34.5	0.3	1.4	101
L	Sesoko	26.65056	127.8549	Hotel, agriculture	09/06/2023	14:00	23	39	38	26.9	34.6	0.2	0.3	122
M	Ginowan	26.27643	127.727	City	12/06/2023	15:00	84	13	2	27.8	33.4	1.1	0.7	120
N	Oodo	26.08846	127.7102	Village, agriculture	11/07/2023	11:00	11	60	28	28.3	34.4	0.1	0.0	106
O	Hanashiro	26.11425	127.7444	Golf course	11/07/2023	15:00	23	48	29	32.0	34.4	0.4	0.6	166
P	Kuba	26.27971	127.8164	Village, agriculture, power plant	12/07/2023	14:30	43	21	35	32.4	34.6	0.2	0.4	140
Q	Agarihama	26.19888	127.7708	City	12/07/2023	16:30	55	17	28	30.4	34.5	2.7	36.2	125
R	Kin	26.44667	127.9136	City, military installation	14/07/2023	10:00	66	14	20	29.7	34.2	0.5	1.4	102

Supplementary Table 2: Substrate characteristics (percentage of live coral, dead coral, macroalgae, seagrass, rubble, sand, rock) and fish community description (density of juveniles and adults of all species, density of *Chrysiptera cyanea* juveniles and adults, number of species, and Shannon diversity index).

Site	Live coral (%)	Dead coral (%)	Macro-algae (%)	Seagrass (%)	Rubble (%)	Sand (%)	Rock (%)	Juvenile density (number of fish per 100 m^2)	Adult density (number of fish per 100 m^2)	Juvenile *C. cyanea* density (number of fish per 100 m^2)	Adult *C. cyanea* density (number of fish per 100 m^2)	Number of species (juveniles)	Number of species (adults)	Number of species (all stages)	Shannon index (juveniles)	Shannon index (adults)	Shannon index (all stages)
A	4	4	1	19	45	20	7	272	68	74	13	12	10	14	0.8	1.3	1.0
B	7	4	0	0	23	10	56	85	225	11	39	11	18	19	1.7	1.6	1.8
C	3	4	5	19	32	19	18	42	18	1	0	7	7	9	1.0	1.3	1.6
D	1	0	15	8	27	26	23	186	66	34	7	11	13	15	1.3	1.7	1.7
E	0	0	12	0	47	20	21	319	131	72	26	12	11	15	1.0	1.2	1.3
F	0	0	0	0	16	19	65	544	220	152	21	17	24	28	0.8	2.0	1.4
G	7	1	7	0	19	4	62	182	227	39	1	10	21	22	1.0	1.9	2.1
H	24	13	1	0	16	11	35	201	142	5	10	19	23	29	1.5	2.4	2.3
I	19	9	3	0	15	8	46	506	153	47	3	15	24	30	1.1	2.5	1.9
J	0	0	0	0	0	0	100	N/A	N/A	N/A	N/A	N/A	N/A	N/A	N/A	N/A	N/A
K	0	0	12	0	0	68	20	N/A	N/A	N/A	N/A	N/A	N/A	N/A	N/A	N/A	N/A
L	4	1	24	1	24	31	15	168	92	27	2	10	19	20	1.3	2.4	2.2
M	0	0	67	0	16	17	0	164	113	41	13	10	6	9	0.8	0.7	1.1

Site	Live coral (%)	Dead coral (%)	Macro-algae (%)	Seagrass (%)	Rubble (%)	Sand (%)	Rock (%)	Juvenile density (number of fish per 100 m^2)	Adult density (number of fish per 100 m^2)	Juvenile C. cyanea density (number of fish per 100 m^2)	Adult C. cyanea density (number of fish per 100 m^2)	Number of species (juveniles)	Number of species (adults)	Number of species (all stages)	Shannon index (juveniles)	Shannon index (adults)	Shannon index (all stages)
N	3	12	4	0	0	48	33	332	187	52	20	17	25	31	1.6	2.3	2.1
O	15	4	16	0	13	7	45	315	354	71	60	21	26	32	1.3	1.7	1.7
P	0	0	21	4	24	33	18	100	32	1	2	12	7	10	1.5	1.3	1.8
Q	0	0	0	0	32	20	48	84	78	1	18	6	9	9	1.1	1.3	1.6
R	17	1	11	0	17	39	15	477	371	38	18	34	19	38	2.2	1.7	2.2

Supplementary Table 3: For each biological process, Spearman correlation coefficient between the coordinate of each sample on the radar plots (Figure 2) and the mean expression level of the genes.

Juveniles		
Category	Number of genes	Correlation
Satiety	45	0.58
Glycolysis	63	0.46
Beta-oxidation	15	0.96
TCA cycle	33	0.95
Urea cycle	7	0.48
Fatty acid synthesis	46	0.19
Cholesterol biosynthesis	26	1
Corticoids	25	0.77
Thyroid hormones	27	0.83
Neurotransmission	127	0.97
Phototransduction	45	0.45
Heat shock proteins	36	0.95
Immunity	403	0.96
Reactive oxygen species	20	0.99
Xenobiotic metabolism	219	0.9

Liver		
Category	Number of genes	Correlation
Satiety	19	0.24
Glycolysis	56	0.27
Beta-oxidation	15	0.92
TCA cycle	31	0.86
Urea cycle	6	0.75
Fatty acid synthesis	39	0.97
Cholesterol biosynthesis	26	1
Corticoids	13	0.26
Thyroid hormones	16	0.92
Heat shock proteins	28	0.93
Immunity	273	0.96
Reactive oxygen species	19	0.84
Xenobiotic metabolism	195	0.96

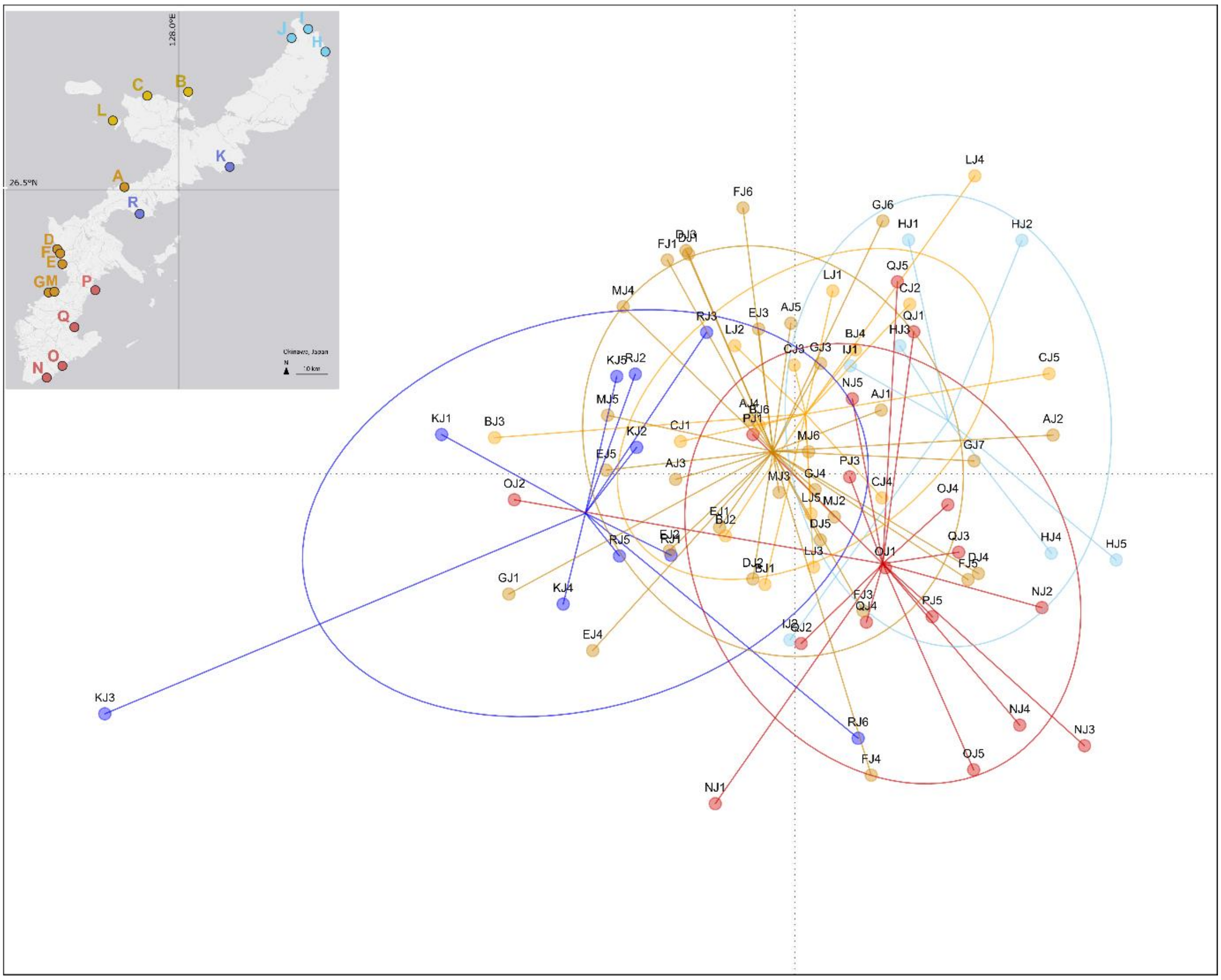

Supplementary Figure 1: First two discriminant functions of a Discriminant Analysis of Principal Components (DAPC) of *C. cyanea* juvenile whole body transcriptome SNPs (n = 90). Colours indicate 5 zones: northwest, north, east, south, west. The analysis is based on filtered SNPs (mind = 0.25, geno = 0.25) following variant calling using the Genome Analysis Toolkit (GATK) pipeline.

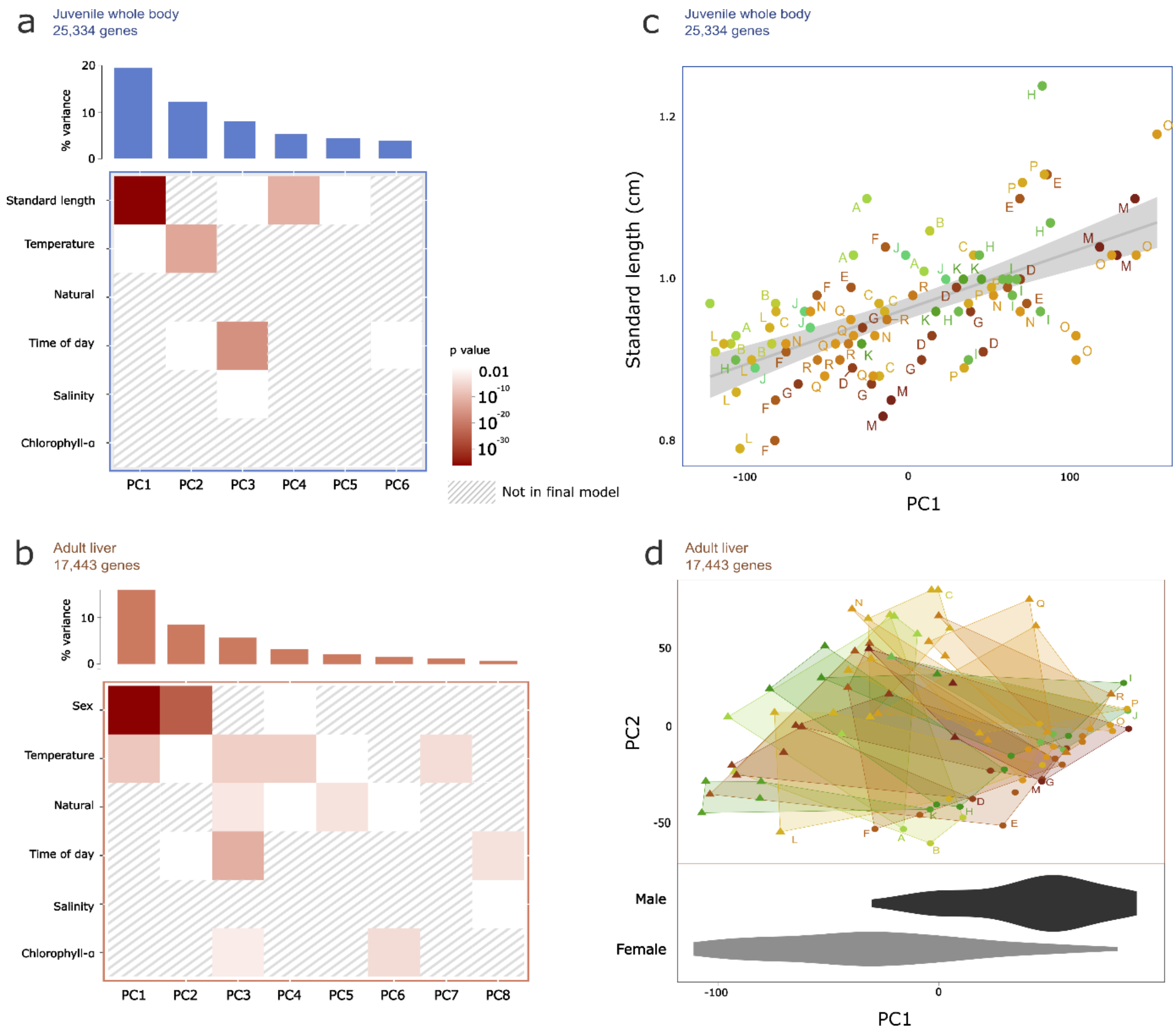

Supplementary Figure 2: Drivers of the overall data structure in two Principal Component Analyses (PCA) for juvenile whole body (n = 90) and adult liver (n = 89) samples separately. For juvenile whole body (**a**) and adult liver (**b**), bar charts of the percentage of contribution to the variance of the top PC dimensions, corresponding to over 50% of the total variance. Tile plots presenting the significance of the linear relationship between various environmental parameters and PC coordinates. Results are based on the best-supported linear-mixed effect models, with sampling site as a random effect. **c**, Relationship between PC1 (PCA with all genes with counts above 10 in at least two sites for juvenile samples) and the standard length of the juvenile fish. Green-yellow-red colours are used to represent the percentage of natural zones within a kilometre of each site with green: most natural; red: least). **d**, Principal Component Analysis of the expression of all genes with normalised counts (from DESeq2 1.36.0) above an average of 10 in at least two sites, for adult liver samples, *i.e.,* for 17,868 genes. Triangles correspond to female livers and circles to male livers. Sex was assigned based on the tail colour of the fish at sampling (females have transparent caudal fins while males have opaque blue caudal fins). Green-yellow-red colours are used to represent the percentage of natural zones within a kilometre of each site with green: most natural; red: least). The bottom panel is a violin plot indicating the distribution of the male and female samples along PC1. There is a significant difference in PC1 values depending on sex (Kruskal-Wallis rank sum test, $\chi^2 = 40.4$, df = 1, $p < 10^{-9}$).

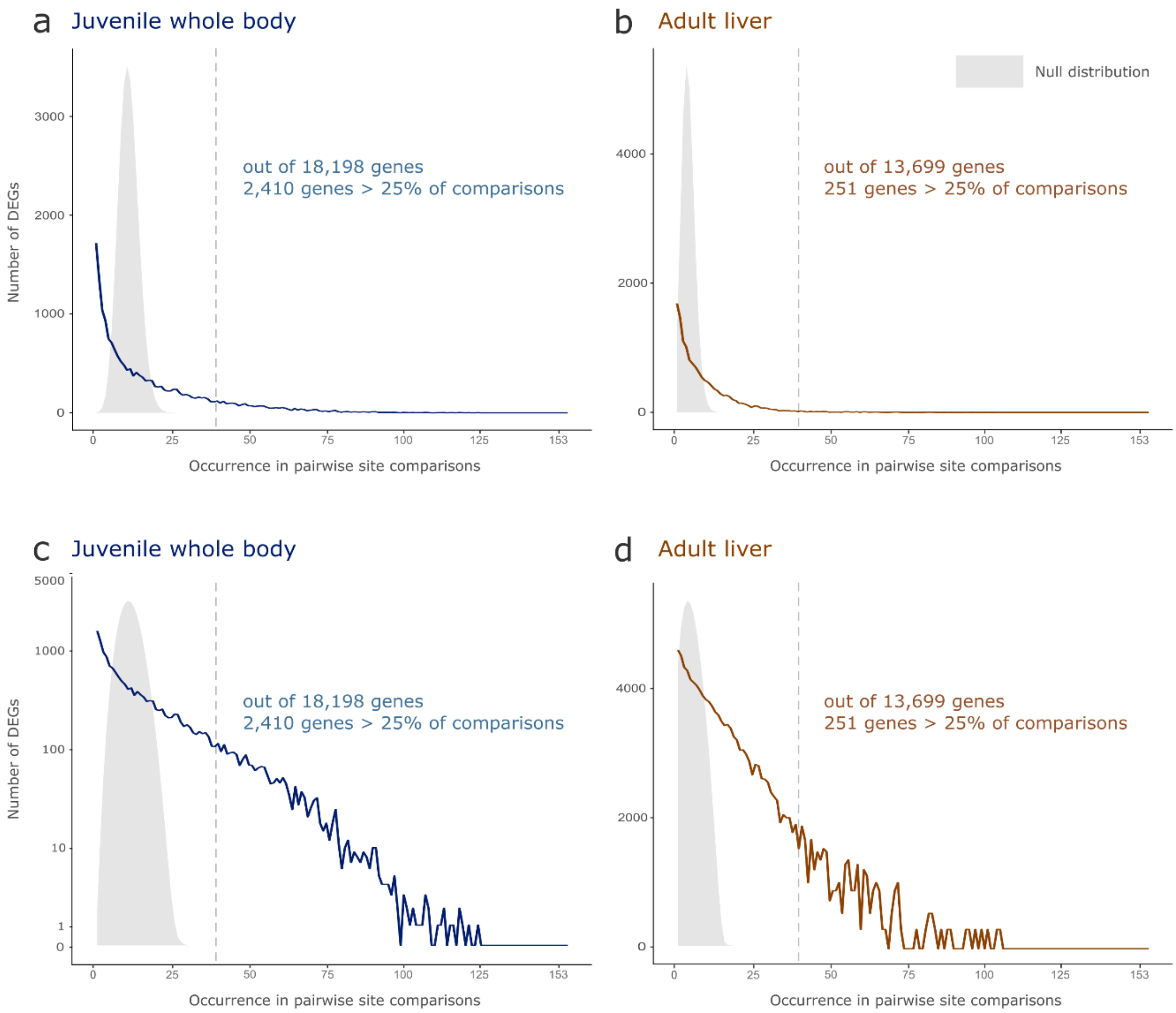

Supplementary Figure 3: Distribution of DEG occurrence across pairwise site comparisons compared with a null expectation. For juvenile whole body (**a** for untransformed DEG counts and **c** for log-transformed counts) and adult livers (**b** for untransformed DEG counts and **d** for log-transformed counts). Colour line: actual number of DEGs (y-axis) repeatedly retrieved in a given number of pairwise site comparisons (x-axis). Grey shading: null distribution derived from 1,000 permutations preserving the number of DEGs per pairwise comparison while randomising gene assignment. The vertical dashed line corresponds to 39 pairwise comparisons (i.e. 25% of all comparisons).

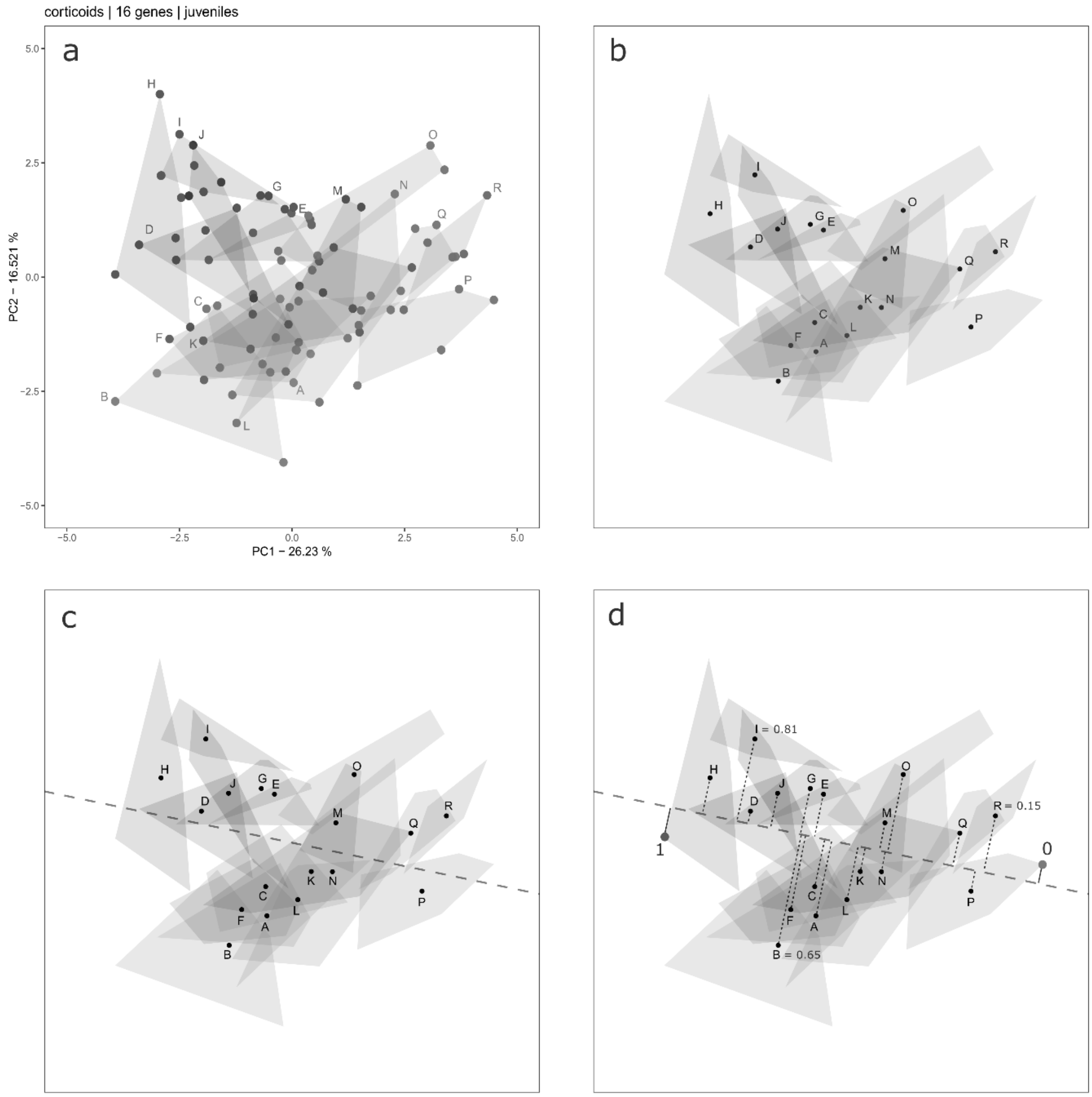

Supplementary Figure 4: Sketch of the method to obtain the radar plots presented in Fig. 4. Here, this is an example where the first two PC dimensions are used; the analysis includes all PC dimensions representing up to 50% of the total variance. **a**, First, the Principal Component Analysis, here based on the normalised expression level of 16 genes linked to corticoids, across 18 sites in Okinawa, with 5 samples per site, is obtained. **b**, The mean PC1/PC2 coordinate of the samples from each of the 18 sites is calculated. **c**, The direction of the main variance in the mean coordinate of the sites is then calculated, here presented as a dashed axis. **d**, The orthogonal projection of all mean site coordinates as well as the projection of each sample onto the main axis of site-wise variance is calculated. The two furthermost samples across the main variance axis are set to have values of 0 and 1. Based on this method, the orthogonal projection of all samples and sites was calculated for multiple gene sets. The results are then summarized as a radar plot in the main manuscript.

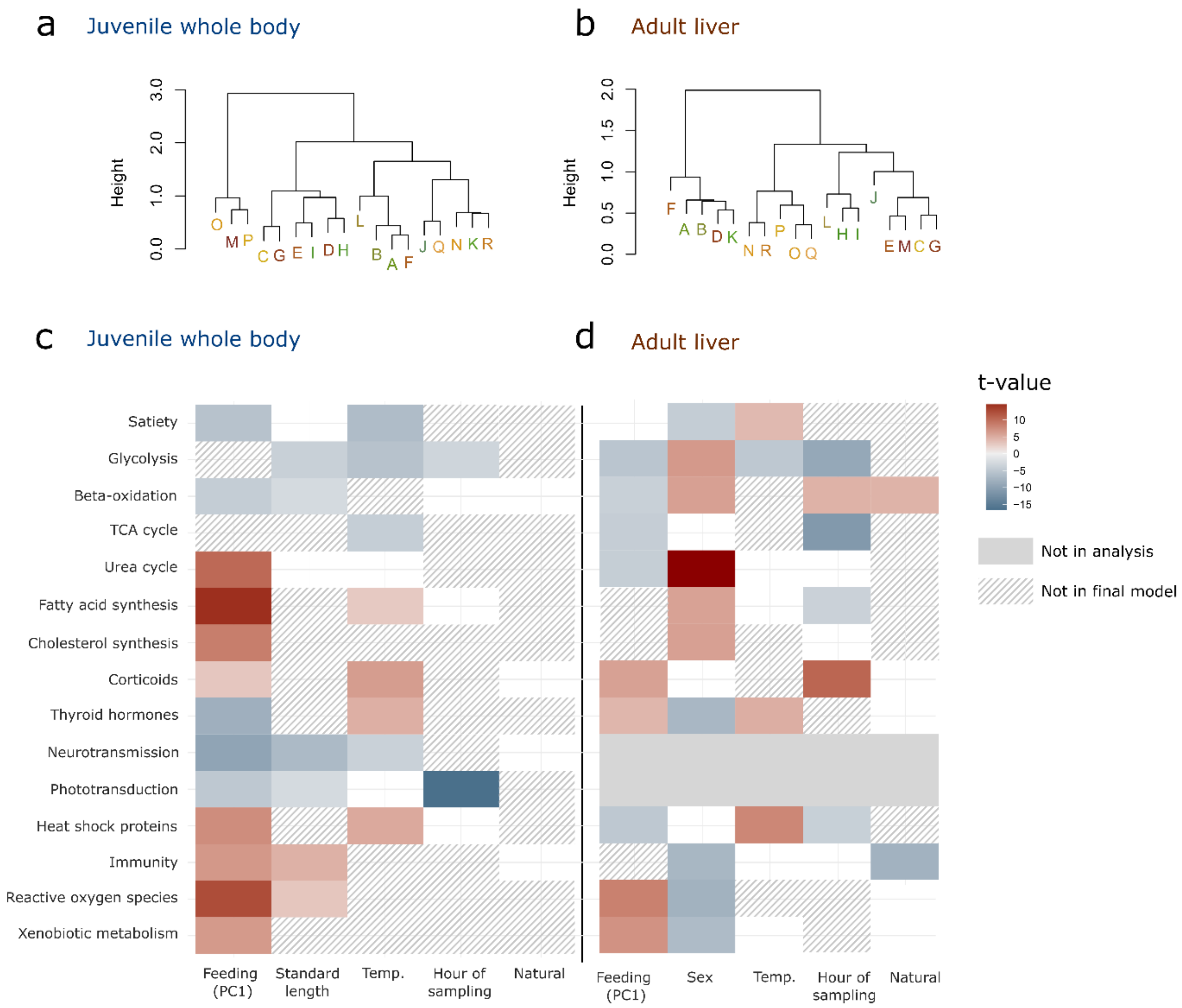

Supplementary Figure 5: Radar profile similarity across sites. Associations between radar plot coordinates, intrinsic fish traits, and environmental variables for each biological process reported on the radar plot. For **a**, juvenile whole body samples (n = 90) and **b**, adult liver samples (n = 89), Euclidean distance between radar plot coordinates at the site level (18 sites; from Figure 2 in the main text). Green-yellow-red colours are used to represent the percentage of natural zones within a kilometre of each site (green: most natural; red: least). For **c** and **d,** associations between radar plot coordinates at the sample level and environmental or intrinsic variables. Variables tested include the percentage of natural zones within a kilometre of the site, feeding status as derived from Fig. 3, standard length for juveniles, sex for adults, temperature, and hour of sampling. Results are based on the best-supported linear-mixed effect models, with sampling site as a random effect. Parameters not retained in the models are indicated with grey hashed tiles, while processes not considered for the liver are indicated with plain grey tiles. Positive t-values (dark red) indicate positive associations between the radar plot coordinate and the corresponding variable, whereas negative t-values (pale blue) indicate inverse associations. Sex (only relevant for adult livers) is presented with positive t-values (dark red) indicating higher radar plot coordinates in females, and negative values (pale blue) higher coordinates in males.

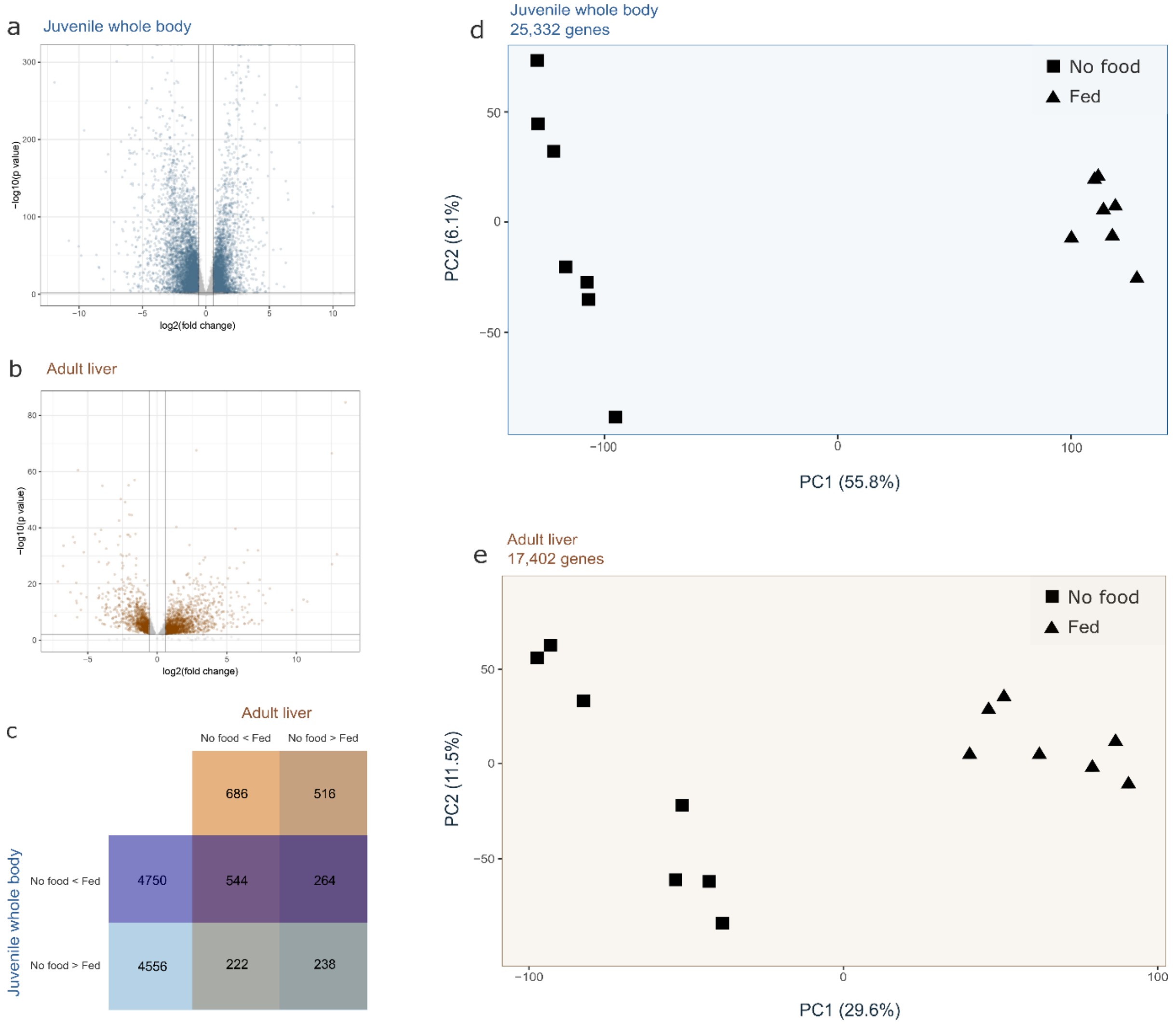

Supplementary Figure 6: Feeding experiment results for the juvenile whole body (n = 7 per treatment) and adult liver (n = 7 per treatment) samples. **a**, Volcano plot displaying significantly differently expressed genes in the whole body (absolute Log2 fold change > 0.58, $p < 0.01$) for the juvenile feeding experiment (no food *vs.* fed). **b**, Volcano plot displaying significantly differently expressed genes in the liver (absolute Log2 fold change > 0.58, $p < 0.01$) for the adult feeding experiment (no food *vs.* fed). **c**, Venn Diagram displaying the number of differentially expressed genes (absolute Log2 fold change > 0.58, adjusted $p < 0.01$) in the juvenile whole body and adult liver samples, with the genes in common shown based on the intersection of the different diagram cells. **d**, Principal Component Analysis of the gene expression level across all genes (with mean expression above 10 counts) for the juvenile feeding experiment samples. **e**, Principal Component Analysis of the gene expression level across all genes (with mean expression above 10 counts) for the liver feeding experiment samples.

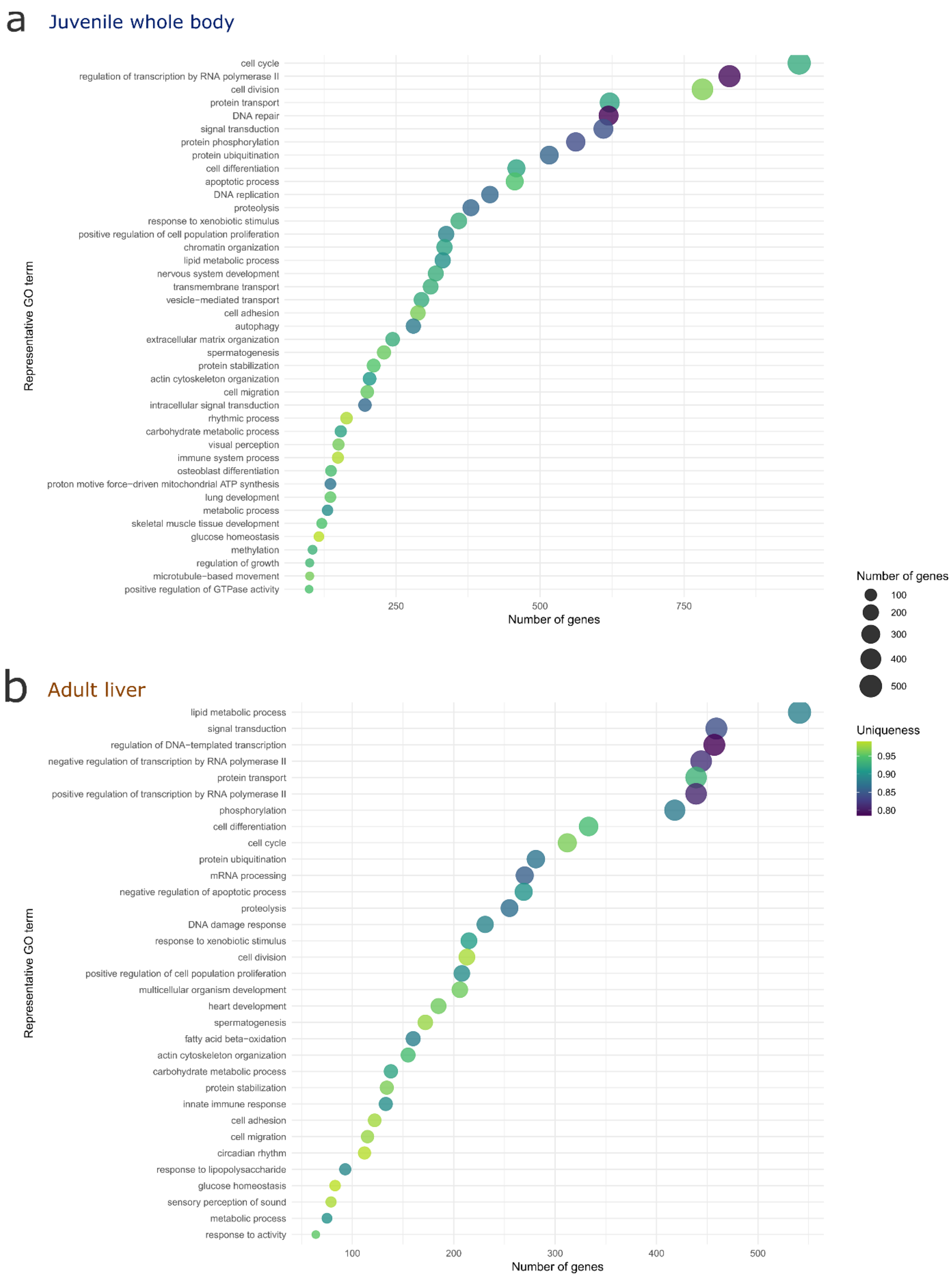

Supplementary Figure 7: Most common GO terms across the top 10 percent genes driving the loading of dimension 1 on the Principal Component Analysis (PCAs) of the feeding experiment samples for juveniles (a) and adult liver (b). The PCAs are presented as Fig. 3a and b. GO terms were trimmed based on semantic similarity at a threshold of 0.7 using GOSemSim 2.28.1. Uniqueness represents how semantically distinct a GO term is from all other high-frequency terms in the dataset.

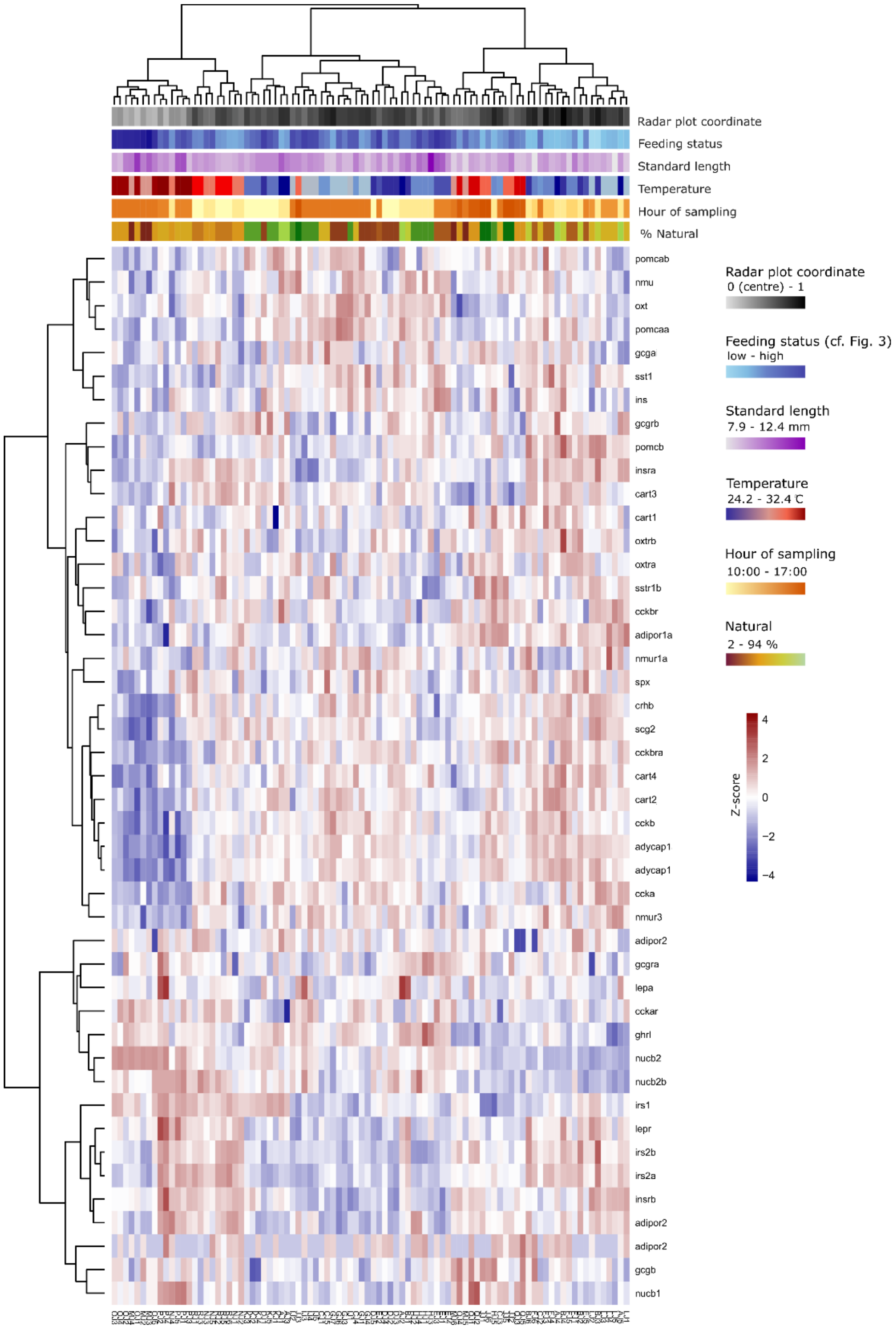

Supplementary Figure 8: Heatmap of the vsd-normalised expression level of 45 appetite-related genes in juvenile field samples (n = 90). Rows are clustered based on correlation using the Ward D2 method with pheatmap 1.0.13. Environmental (temperature, hour of sampling) and intrinsic fish variables (feeding status, standard length) are indicated above the heatmap. Increasingly dark colours indicate higher values for the radar plot coordinates, feeding status, standard length, and hour of sampling. Temperature is indicated from a blue (low temperature) to red (high temperature) scale.

a

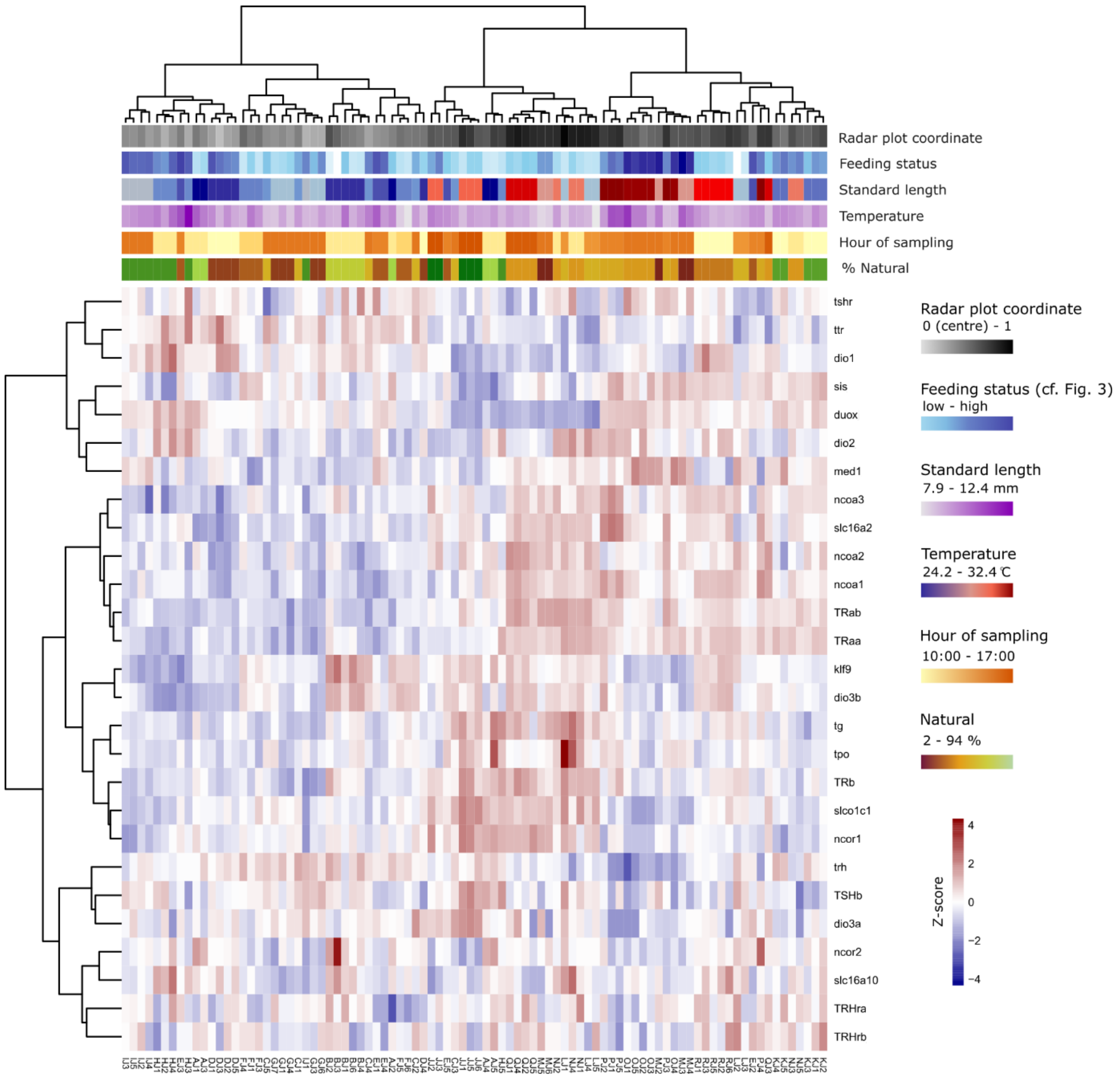

Supplementary Figure 9: Heatmap of the vsd-normalised expression level of 27 thyroid-related genes in juvenile field samples (n = 90). Rows are clustered based on correlation using the Ward D2 method with pheatmap 1.0.13. Environmental (temperature, hour of sampling) and intrinsic fish variables (feeding status, standard length) are indicated above the heatmap. Increasingly dark colours indicate higher values for the radar plot coordinates, feeding status, standard length, and hour of sampling. Temperature is indicated from a blue (low temperature) to red (high temperature) scale.

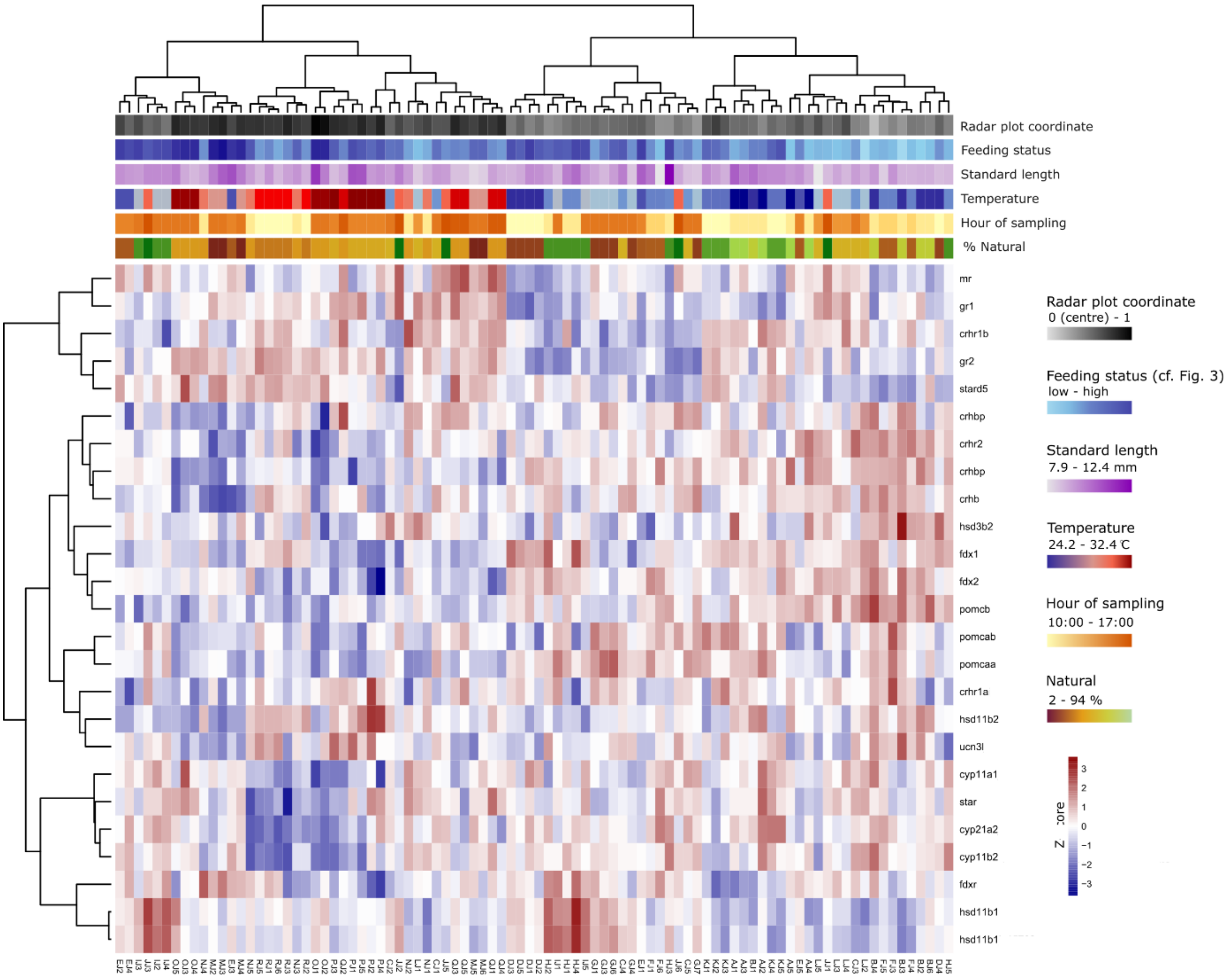

Supplementary Figure 10: Heatmap of the vsd-normalised expression level of 25 corticoid-related genes in juvenile field samples (n = 90). Rows are clustered based on correlation using the Ward D2 method with pheatmap 1.0.13. Environmental (temperature, hour of sampling) and intrinsic fish variables (feeding status, standard length) are indicated above the heatmap. Increasingly dark colours indicate higher values for the radar plot coordinates, feeding status, standard length, and hour of sampling. Temperature is indicated from a blue (low temperature) to red (high temperature) scale.

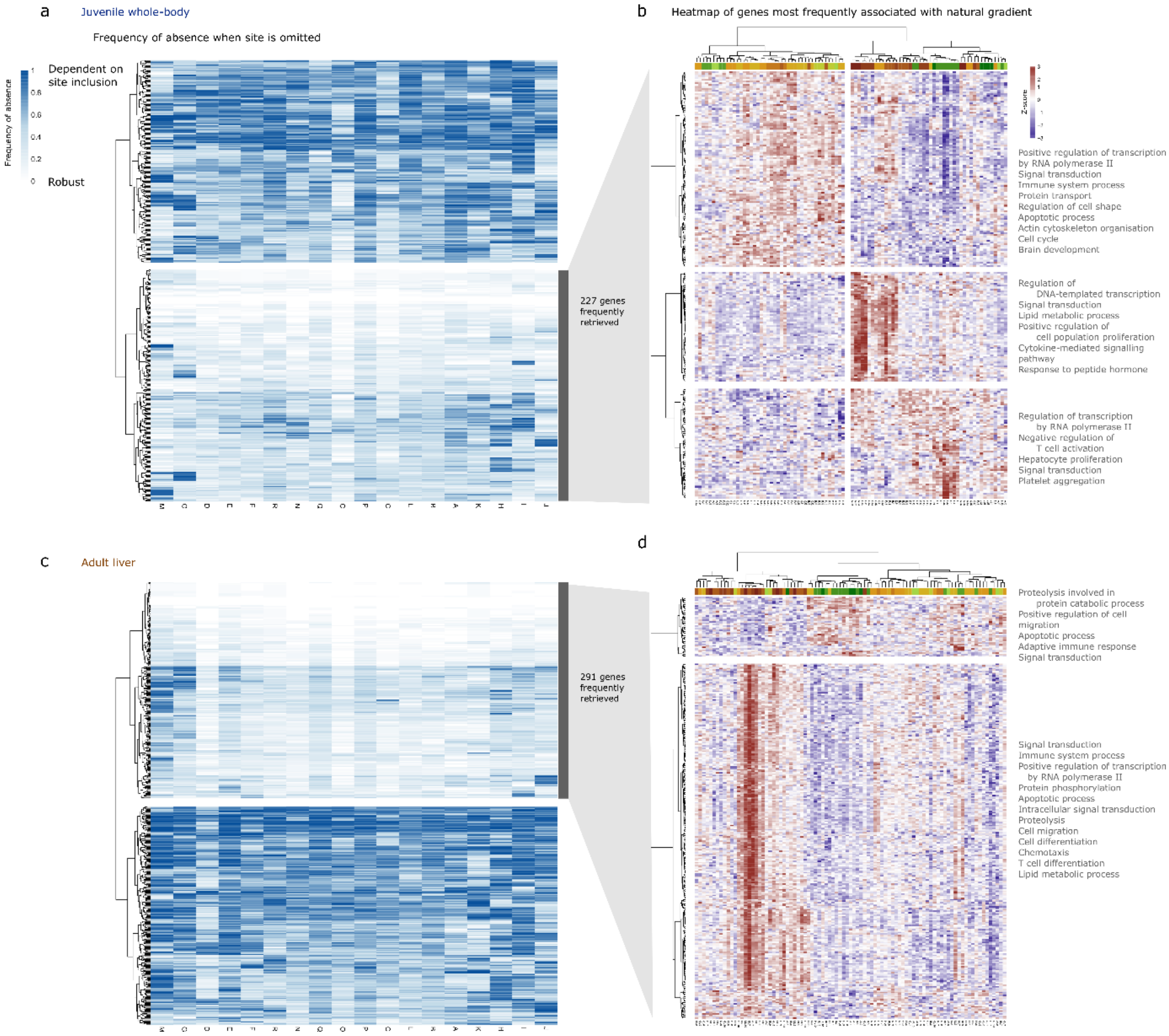

Supplementary Figure 11: Robustness of the list of genes showing linear or quadratic relationships with the percentage of natural zones near each site (18 sites in total) in juvenile whole body (n = 90) and adult liver (n = 89) transcriptomes. Linear and quadratic models were run 100 times with 1 to 6 sites removed per model. **a,** Heatmap of the frequency at which genes (as rows) were not retrieved when excluding a given site (as columns, 18 sites in total. Green-yellow-red colours are used as column labels to represent the percentage of natural zones within a kilometre of each site with green: most natural; red: least) for juvenile whole-body transcriptomes. Genes that were retrieved consistently have clearer colours. **b,** Heatmap of the 227 genes frequently retrieved; Gene Ontology biological processes corresponding to the 3 main gene clusters, presented in order of prevalence within each gene cluster. **c,** Heatmap of the frequency at which genes (as rows) were not retrieved when excluding a given site (as columns, 18 sites in total. Green-yellow-red colours are used as column labels to represent the percentage of natural zones within a kilometre of each site with green: most natural; red: least) for adult liver transcriptomes. Genes that were retrieved consistently have clearer colours. **d,** Heatmap of the 291 genes frequently retrieved; Gene Ontology biological processes corresponding to the 2 main gene clusters, presented in order of prevalence within each gene cluster.

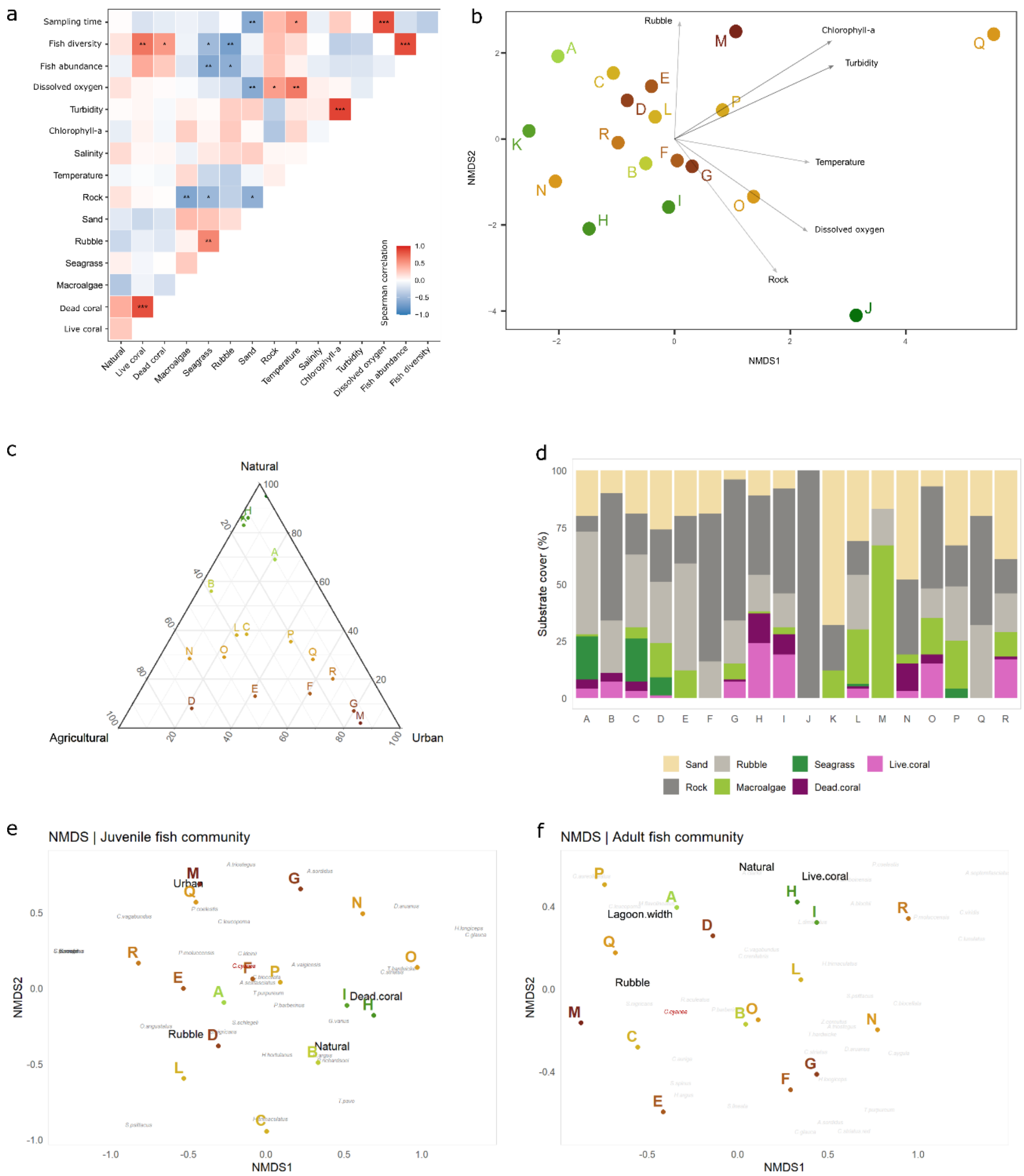

Supplementary Figure 12: Environmental characterisation of the 18 sites. Green-yellow-red colours are used to represent the percentage of natural zones within a kilometre of each site (green: most natural; red: least). a, Spearman correlation coefficient between the environmental variables. Significance is indicated as * ($p < 0.05$), ** ($p < 0.01$), *** ($p < 0.001$). **b,** NMDS analysis of the environmental characteristics of the 18 sites and contributing variables based on vegan 2.5-7 function envfit ($p < 0.05$). **c**, Ternary plot presenting the percentage of natural, agricultural, and urban zones within 1km of the 18 sites (excluding water). **d**, Substrate cover on three 25m transects performed parallel to shore near sampling area for 16 sites (maximum depth: 2m). **e**, NMDS analysis of the juvenile fish community structure and contributing environmental variables based on vegan 2.5-7 function envfit for 16 sites ($p < 0.1$). **f**, NMDS analysis of the adult fish community structure and contributing environmental variables based on vegan 2.5-7 function envfit for 16 sites ($p < 0.1$). Note that no fish community structure was recorded on sites J and K due to logistical constraints.
