# Supplementary material for: Landscape transcriptomics reveals ecological constraints on fish under anthropogenic coastal change
Source: Nat Commun. 2026 Oct 9;17:10218. doi: 10.1038/s41467-026-77751-2 (PMC13652805; doi:10.1038/s41467-026-77751-2)
Supplement: Supplementary file 2 [file 41467_2026_77751_MOESM2_ESM.pdf]

<u>Description of Additional Supplementary Files</u>

Supplementary Data 1: Sample description of the juveniles from the field.

Supplementary Data 2: Sample description of the adults from the field.

Supplementary Data 3: Sample description of the juveniles from the feeding experiment.

Supplementary Data 4: Sample description of the adults from the feeding experiment.

Supplementary Data 5: Quality check of the RNA integrity of the samples from the field.

Supplementary Data 6: Quality check of the RNA integrity of the juvenile whole body and adult liver samples from the feeding experiment.
