# Supplementary material for: Landscape transcriptomics reveals ecological constraints on fish under anthropogenic coastal change
Source: Nat Commun. 2026 Oct 9;17:10218. doi: 10.1038/s41467-026-77751-2 (PMC13652805; doi:10.1038/s41467-026-77751-2)
Supplement: Supplementary file 3 [file 41467_2026_77751_MOESM4_ESM.pdf]

nature portfolio

Corresponding author(s): Emma Gairin

Last updated by author(s): 9/01/2026

Reporting Summary

Nature Portfolio wishes to improve the reproducibility of the work that we publish. This form provides structure for consistency and transparency in reporting. For further information on Nature Portfolio policies, see our Editorial Policies and the Editorial Policy Checklist.

Statistics

For all statistical analyses, confirm that the following items are present in the figure legend, table legend, main text, or Methods section.

n/a	Confirmed	
☐	☒	The exact sample size (*n*) for each experimental group/condition, given as a discrete number and unit of measurement
☐	☒	A statement on whether measurements were taken from distinct samples or whether the same sample was measured repeatedly
☐	☒	The statistical test(s) used AND whether they are one- or two-sided<br>*Only common tests should be described solely by name; describe more complex techniques in the Methods section.*
☐	☒	A description of all covariates tested
☐	☒	A description of any assumptions or corrections, such as tests of normality and adjustment for multiple comparisons
☐	☒	A full description of the statistical parameters including central tendency (e.g. means) or other basic estimates (e.g. regression coefficient) AND variation (e.g. standard deviation) or associated estimates of uncertainty (e.g. confidence intervals)
☐	☒	For null hypothesis testing, the test statistic (e.g. F, t, r) with confidence intervals, effect sizes, degrees of freedom and P value noted<br>*Give P values as exact values whenever suitable.*
☒	☐	For Bayesian analysis, information on the choice of priors and Markov chain Monte Carlo settings
☐	☒	For hierarchical and complex designs, identification of the appropriate level for tests and full reporting of outcomes
☐	☒	Estimates of effect sizes (e.g. Cohen's *d*, Pearson's *r*), indicating how they were calculated

Our web collection on statistics for biologists contains articles on many of the points above.

Software and code

Policy information about availability of computer code

Data collection

Sequencing data used in the manuscript were generated using an Illumina SNovaSeq6000 platform at the OIST Sequencing section. Paired end reads (2 x 150 bp) were obtained.

Data analysis

Quality control of the RNA sequencing data was performed with FastQC. Following this quality check, the data was processed by trimming the adapters used by Illumina on each transcript as well as dropping low quality sections with Trimmomatic 0.39. The sequences were pseudo-paligned to the reference transcriptome for this species (consisting of the longest isoforms for each gene) and the number of reads per gene were quantified using Kallisto 0.46.2.
An annotated genome is available for Chrysiptera cyanea with 28,173 annotated genes, of which 26,300 have corresponding gene names identified using BLAST (cf. Gairin et al. 2024 Gigabyte).
For all subsequent data analysis, R 4.3.3 was used.

Plots were generated using ggplot2 3.5.1, ggtern 3.5.0, ggradar 0.2, pheatmap 1.0.12.
Principal Component Analyses (PCA) were obtained with the prcomp function from stats 4.3.3
Gene counts were normalised (vsd) using DESeq2 1.36.0.
GO terms were analysed semantically using GoSemSim 2.28.1.

Statistical tests included the non-parametric Kruskal-Wallis Rank Sum test (stats 4.3.3) and linear mixed effect models (lme4 1.1-38) with environmental variables, fish size or sex as fixed effects and site as a random effect, using the top parameters identified with the regsubset model selection function of leaps 3.2.

To estimate diet in juvenile C. cyanea, using Kaiju 1.10.0, juvenile whole-body reads that did not map to the reference C. cyanea

transcriptome were compared with 2,462 reference NCBI protein sequences available for the following taxa: Artemia (NCBI ID: 6660), Chlorophyta (3041), Ctenophora (10197), Cyanobacteria (1117), Dinoflagellata (2864), Embrophyta (3193), Fungi (4751), Haptophyta (2830), Ochrophyta (2696291), Pancrustacea (197562), Rhodophyta (2763), Tunicata (7712). The proportion of reads in each sample corresponding to different taxonomic families was identified.

For population genetics, the raw sequencing reads of juvenile whole-body samples were mapped to the reference genome using 2-pass mapping using STAR 2.7.9a followed by data cleaning with Picard (AddOrReplaceReadGroups, MarkDuplicates). GATK 4.4.0.0 was then used for variant calling following the pipeline from Genomics Core at NYU CGSB. SNPs were filtered to exclude low quality variants. Principal Component Analysis was performed using PLINK, removing samples with over 25% of missing genotype data (11 samples out of 90) and SNPs with missing genotype rates higher than 25% (9,040 variants retained). Discriminant Analysis of Principal Components (DAPC) was performed in R using adegenet 2.1.10.

To evaluate whether genes were repeatedly differentially expressed (DE) across sites more frequently than expected by chance, we generated an empirical null distribution based on 1,000 permutations where each pairwise site comparison column was independently shuffled without replacement, preserving the overall number of DEGs detected in each pairwise site comparison while randomising gene identity. For each permutation, gene recurrence counts were recalculated to establish the expected null distribution. We compared the empirical cumulative distribution of observed gene recurrences (Figure 1e) against the permuted null distribution using a two-sample Kolmogorov–Smirnov test (ks.test in stats 4.3.382). Tail significance was evaluated using empirical permutation p-values, defined as the proportion of the 1,000 null iterations where the count of high-recurrence genes (>25% of comparisons, i.e., 39 comparisons) met or exceeded the observed count.

For manuscripts utilizing custom algorithms or software that are central to the research but not yet described in published literature, software must be made available to editors and reviewers. We strongly encourage code deposition in a community repository (e.g. GitHub). See the Nature Portfolio guidelines for submitting code & software for further information.

Data

Policy information about availability of data

All manuscripts must include a data availability statement. This statement should provide the following information, where applicable:

- Accession codes, unique identifiers, or web links for publicly available datasets
- A description of any restrictions on data availability
- For clinical datasets or third party data, please ensure that the statement adheres to our policy

Raw sequencing datasets generated in this study have been deposited in the NCBI BioProject repository under accession codes: PRJNA1168661 (field samples; https://www.ncbi.nlm.nih.gov/bioproject/PRJNA1168661/), PRJNA1271234 (juvenile feeding samples; https://www.ncbi.nlm.nih.gov/bioproject/PRJNA1271234/), PRJNA1168266 (adult feeding samples; https://www.ncbi.nlm.nih.gov/bioproject/PRJNA1168266/). Land use datasets are available from JAXA's High-Resolution Land Use and Land Cover Map of Okinawa Island (2023) at https://www.eorc.jaxa.jp/ALOS/en/dataset/lulc/lulc_v2301_e.htm. All processed data and R scripts are publicly available on figshare, https://doi.org/10.6084/m9.figshare.27966972

Research involving human participants, their data, or biological material

Policy information about studies with human participants or human data. See also policy information about sex, gender (identity/presentation), and sexual orientation and race, ethnicity and racism.

Reporting on sex and gender	n/a
Reporting on race, ethnicity, or other socially relevant groupings	n/a
Population characteristics	n/a
Recruitment	n/a
Ethics oversight	n/a

Note that full information on the approval of the study protocol must also be provided in the manuscript.

Field-specific reporting

Please select the one below that is the best fit for your research. If you are not sure, read the appropriate sections before making your selection.

☐ Life sciences ☐ Behavioural & social sciences ☒ Ecological, evolutionary & environmental sciences

For a reference copy of the document with all sections, see nature.com/documents/nr-reporting-summary-flat.pdf

Ecological, evolutionary & environmental sciences study design

All studies must disclose on these points even when the disclosure is negative.

Study description	The objective of the study was to determine the impact of human presence and other environmental drivers on coastal fish.

Study description

Chrysiptera cyanea ranks among the most abundant fish species in shallow coastal zones of Okinawa. Juveniles (2-3 weeks post arrival on the reef) and adults (~ 1 year old, both females and males) were sampled in the field (90 juveniles, 89 adults, 5 per sites for each type apart from 4 adults on Site B due to logical issues, 18 sites in total).

The laboratory study is based on a feeding experiment with a fasted treatment and a control (normal food, size/weight remained stable throughout) following an acclimation period for fish that were caught in Okinawa, for both juveniles (3-day experiment) and adults (14-day experiment).

Research sample

Field samples: 179 Chrysiptera cyanea fish (90 juveniles 0.80-1.24cm, 89 adults 2.35-5.11cm SL). 4-5 fish were collected from 18 sites at each developmental stage.

For juveniles, the whole body for sequenced due to the small size of the samples (logistical field constraints for dissections) and in order to evaluate the impact of environmental conditions across all genes of the body. For adults, we focused on the metabolically active, detoxifying liver.
Focusing on 18 sites, with 4-5 fish per sites, allowed to maximise the number of sites while maintaining sufficient statistical power to assess the impact of each site on the fish transcriptomes.

For the feeding experiment, we obtained the transcriptomes of 7 fasted juveniles, 7 fed juveniles, 7 fasted adults, 7 fed adults. For adults, we used only the liver tissue. 7 samples per treatment provided sufficient statistical power for analyses.

Sampling strategy

On 18 sites along the coast of Okinawa, 5 juvenile and 5 adult C. cyanea (4 on site B) were collected using hand nets and snorkelling or SCUBA diving. In total, 90 juveniles, 49 females, and 40 males were collected. A minimum of 4 fish per site was collected to ensure sufficient site-specific statistical power. This is based on common practices for fish transcriptomics. 18 sites were used to encapsulate a broad range of variable combinations and disentangle the contribution of various fish-specific and environmental parameters to gene expression differences. For the experimental data, 7 samples were used to minimise individual variability impacts on the detection of feeding effects on gene expression.

The fish were euthanised in a 200mg/L Tricaine Methanesulfonate (MS222) solution following the guidelines for animal use issued by the Animal Resources Section of OIST Graduate University. Juvenile fish were photographed and directly placed in RNAlater for tissue preservation. Adult fish were photographed, weighed, and dissected on-site. The livers were preserved using RNAlater.
For the laboratory experiment, after the treatment ended, each fish was weighed, measured, euthanised with MS222, and dissected. The whole body of each individual was preserved in RNAlater for juveniles and livers for adults. For juveniles, we used 14 fish per treatment during the experiment and selected 7 fish for sequencing based on RNA extraction quality. For adults we used 8 fish per treatment during the experiment and selected 7 fish for sequencing based on RNA extraction quality.

Data collection

Samples were collected by Emma Gairin, Camille Sautereau, Zoe Chamot, Jann Zwahlen, Hiroki Takamiyagi using SCUBA diving and snorkelling in Okinawa, Japan.

Samples were dissected and preserved by Saori Miura.

On each site, using a multiparameter probe (RINKO-Profiler CTD logger), water temperature, salinity, dissolved oxygen, turbidity, and chlorophyll-α were measured. Emma Gairin, Camille Sautereau, Zoe Chamot, Jann Zwahlen, Hiroki Takamiyagi collected this dataset.

Three replicate 25m long x 4m wide transects were performed to record the fish community. These transects were positioned parallel to the shore, ten meters apart, in at least one meter of water. Transects could not be conducted at two sites, Uka (J) and Kayo (K), due to limited water depth or strong currents. Two passes were performed per transect; mobile, more visible fishes were recorded during the first pass and more cryptic fishes during the second pass. All fish were identified to the species level. The average fish density (number of fish per m^2) and species richness (number of species per m^2) were calculated. Additionally, the substrate cover on each site was assessed across the three transects using a point-intercept method, with the type of substrate (live coral, dead coral, macroalgae, seagrass, rubble, hard substrate, sand) recorded every meter. Emma Gairin counted the fish.

Land use in the watersheds near each site was assessed based on a publicly available dataset released in January 2023 from JAXA. The percentage of urban, agricultural, and natural zones within a radius of 1km of the sites (excluding water) was calculated for each site.

Timing and spatial scale

One time sampling of 18 sites all around the main island of Okinawa, Japan during the summer of 2023 (24 May - 14 July), which is the reproduction period of Chrysiptera cyanea and thus allowed to collect juveniles upon their return to the reef, following a 2-3 week open ocean dispersal period as larvae. 18 sites were targeted to maximise the number of combinations of environmental parameters and capture a gradient of human presence in Okinawa.

For the feeding experiment, juveniles were acclimated overnight (to have similar developmental stages as the field samples) and the treatment lasted 3 days as this was the duration that allowed to detect a sub-lethal effect of fasting (experiment dates: 31 July - 2 August 2024). For adults, fish were acclimated over a month and the fasting experiment lasted 2 weeks (23 November 2022-6 December 2022).

Data exclusions

No excluded data.

Reproducibility

In terms of sequencing data, sequencing depth and sample quality may lead to different gene expression profiles if a similar experiment was repeated.

For field samples, we used 5 fish per location were collected to assess intrinsic variability. If sampled again, fish on each site may show different results at a different time, depending on which genes are examined (due to effects of season, water temperature, weather, etc, as explored in the manuscript).

7 fish were sampled for the laboratory experiment each for juveniles and adults for each treatment to assess variability within each treatment. Seasonal, development, and reproduction cycles (for adults) may lead to differences in gene expression profiles if a similar experiment was repeated, although the overall signal of food availability would remain consistent.

All details about the experiment are provided for reproducibility.
All data and codes used in the study are made available on NCBI and Figshare so that the results can be reproduced.

Randomization

For field sampling, 4-5 adult fish and 5 juvenile fish were collected at random near shore.
For laboratory experiments, fish were weighed and split into groups to obtain homogenous weight distributions across the fish in each treatment at the start of the experiment.

Blinding

n/a

Did the study involve field work? ☒ Yes ☐ No

Field work, collection and transport

Field conditions

Fieldwork was conducted along the shore of mainland Okinawa in depths of 0-2 meters and at a distance <50 meters from shore. No rainfall occurred within 3 days of sampling. Temperature of the water ranged between 24.2 and 32.4 oC. All information is provided in Table S1.

Location

Site Site name Latitude (N) Longitude (E)
A Seragaki 26.50514 127.8801
B Kouri 26.71363 128.0197
C Nakijin 26.70474 127.9302
D Toguchi 26.36946 127.7332
E Junkyard 26.33682 127.7444
F Mizugama 26.36009 127.7398
G Minatogawa 26.27478 127.7143
H Adan 26.80111 128.3195
I Oku 26.85067 128.2821
J Uka 26.83113 128.2459
K Kayo 26.54939 128.1106
L Sesoko 26.65056 127.8549
M Ginowan 26.27643 127.727
N Oodo 26.08846 127.7102
O Hanashiro 26.11425 127.7444
P Kuba 26.27971 127.8164
Q Agarihama 26.19888 127.7708
R Kin 26.44667 127.9136

Access & import/export

We communicated with local fisheries association and coastguards for fieldwork on all locations.

Disturbance

No disturbance was caused by the fieldwork on the ecosystem.

Reporting for specific materials, systems and methods

We require information from authors about some types of materials, experimental systems and methods used in many studies. Here, indicate whether each material, system or method listed is relevant to your study. If you are not sure if a list item applies to your research, read the appropriate section before selecting a response.

Materials & experimental systems

n/a	Involved in the study
☒	☐ Antibodies
☒	☐ Eukaryotic cell lines
☒	☐ Palaeontology and archaeology
☐	☒ Animals and other organisms
☒	☐ Clinical data
☒	☐ Dual use research of concern
☒	☐ Plants

Methods

n/a	Involved in the study
☒	☐ ChIP-seq
☒	☐ Flow cytometry
☒	☐ MRI-based neuroimaging

Animals and other research organisms

Policy information about studies involving animals; ARRIVE guidelines recommended for reporting animal research, and Sex and Gender in Research

Laboratory animals	Chrysiptera cyanea, collected from Okinawa, Japan and maintained at the OIST Marine Science Station.<br>Juvenile (< 2 months) and adult females (> 6 months).
Wild animals	In shallow coastal zones of Okinawa, juvenile and adult C. cyanea were collected using hand nets and snorkelling or SCUBA diving. In the field the fish were collected by SCUBA diving using hand nets, transported to shore in wide 15L buckets, and euthanised using MS222 (200mg/L) following standard practices and the guidelines for animal use issued by the Animal Resources Section of OIST Graduate University.
Reporting on sex	90 juveniles, 49 females, and 40 males were collected. Analysis was done carefully to detect the effect of sex on adult liver transcriptomes.
Field-collected samples	Juveniles collected in Apogama, Okinawa, Japan (26.50003 N, 127.84261 E), on 30 July 2024 were kept at the OIST Marine Science Station. After one night of acclimation, juveniles were placed in two tanks with 14 juvenile fish per tank and a three-day experiment began (fasting vs. feeding, July 31-August 2nd 2024).<br><br>For adults, female C. cyanea collected in Sesoko, Okinawa, Japan (26.65056 N, 127.8549 E), on 30 September 2022 were kept at the OIST Marine Science Station. After over 50 days of acclimation, they were placed in two tanks with eight fish per tank for the 14-day experiment (23 November - 6 December).<br><br>For group allocation of the experiments, each fish was weighed before the start of the experiment and distributed across the treatments to ensure that there was no statistical difference in weight distribution prior to the experiment.<br><br>All feeding experiment fish were maintained at the Okinawa Institute of Science and Technology Marine Science Station in standard 30L tanks with constant seawater input (pumped from 10m depth near the station). Photoperiod was 6AM-6PM.<br><br>Following a 3-day (juvenile) and 14-day (adults) feeding experiment, the fish were euthanised in a 200mg/L Tricaine Methanesulfonate (MS222) solution following the guidelines for animal use issued by the Animal Resources Section of OIST Graduate University. Throughout the acclimation and experiment, water input came from pumped seawater (filtered with a fine mesh for the fasted treatment to minimise plankton abundance in the tank).
Ethics oversight	The guidelines for animal use issued by the Animal Resources Section of OIST Graduate University were followed for animal handling in the field. Ethical approval for the laboratory experiments was granted by the Okinawa Institute of Science and Technology (ACUP-2022-388, ACUP-2023-014).<br>Field sampling was conducted in accordance with standard ecological protocols and with approval from the Okinawa Institute of Science and Technology (FWA-2022-008). All efforts were made to minimise animal distress during handling. The fish were euthanised in a 200mg/L Tricaine Methanesulfonate (MS222) solution following the guidelines for animal use issued by the Animal Resources Section of OIST Graduate University.

Note that full information on the approval of the study protocol must also be provided in the manuscript.

Plants

Seed stocks	n/a
Novel plant genotypes	n/a
Authentication	n/a
