# Supplementary material for: Landscape transcriptomics reveals ecological constraints on fish under anthropogenic coastal change
Source: Nat Commun. 2026 Oct 9;17:10218. doi: 10.1038/s41467-026-77751-2 (PMC13652805; doi:10.1038/s41467-026-77751-2)
Supplement: Supplementary file 4 [file 41467_2026_77751_MOESM5_ESM.pdf]

nature portfolio

# Peer Review File

## Landscape transcriptomics reveals ecological constraints on fish under anthropogenic coastal change

Corresponding Author: Dr Emma Gairin

**This file contains all reviewer reports in order by version, followed by all author rebuttals in order by version.**

Version 1:

Reviewer comments:

Reviewer #1

(Remarks to the Author)
This was an ambitious and well-done study that truly shows the potential of landscape transcriptomics to inform how wild organisms respond to complex sets of environmental stressors. It uses a nice combination of field-collected samples across environmental gradients, lab-based controlled experiments, and data mining of the literature to come to the interesting conclusion that damselfish near urban centers are highly stressed but overfed, which the authors compare colorfully to living on a “fast-food diet”. In addition, the development of radar plots as a visualization and quantification tool for assessing stressors at different sites has great potential as a practical conservation and management tool. I found enjoyable to read which is often a feat for transcriptomics papers.

I have a couple areas for improvement I’d like to highlight.
First, while I strongly agree that the radar plots represent an advance in leveraging transcriptomes to identify environmental stressors, how they were created wasn’t well described and I found the limited descriptions in the paper and methods confusing (and I like to think that I am quite adept at multivariate statistics and linear algebra). In fact, it was the supplemental figure that best illustrated what was going on. I’d suggest including some aspects of that figure in the main paper and also revisiting the wording. For example, consider clarifying that the axis of maximum variance is in fact a multivariate axis of maximum variance (if I’m understanding correctly). Also importantly so that others can actually utilize this contribution, how is that step done? For example, in the supplementary figure, it says “The direction of the main variance in the mean coordinate of the sites is then calculated, here presented as a dashed axis,” but this is rather vague. Are you running a new PCA on your (reduced number of) PCs? Or is it some kind of factor rotation? This detail is very important for future adoption.
Second, I can’t figure out at all how WGCNA was utilized in this study. Was it another analysis that just wasn’t featured in the main text? I do commend you on using linear models in the supplement and not relying on the standard and very limited correlation table approach many other people still use.
Third, I was unclear on why a secondary PCA had to be done for the analyses comparing experimental food deprivation to wild-caught samples. If you know the loadings then you could just apply those to the wild-caught gene count matrix (it’s simple matrix algebra).

More minor or specific comments:
Abstract
Lines 32-34: “We uncover a striking molecular signature of urbanization: fish from human-impacted sites exhibit elevated expression of genes involved in inflammation and immune responses.” I’d delete this sentence. It is repetitive with the sentences that follow and I found it made this part of the abstract confusing.

Main
Lines 68-70: “after a period of larval dispersal in the open ocean, different species may prefer specific habitat features – such as higher nutrient availability or stable temperatures – when settling on a reef.” I don’t see how this fits here, consider deleting or moving. I found it distracting.
Lines 110-112: “As only one organ was considered for adults, the number of genes and range of physiological responses expressed in the samples was lower than for juvenile whole-body samples.” I'm not sure this had to be the case, however. Often whole body or tissue sampling is expected to obscure tissue-level or cell-level differences, respectively.

Line 130: You could statistically test whether there was clustering by sites as seems apparent from the figure (e.g., PERMANOVA).
Line 132: Did the quadratic model also have a linear component?
Line 142-144: I am having trouble picturing what is actually being compared to get this statistic?
Line 146: What does "multiple biological levels" refer to? Cells, tissues, organs? What is this statement based on?
Line 155: "do not respond to other environmental or organismal factors" clarify that this statement is based on your data.

Figure 1
a: Density here refers to density of points in the figure?
c: I don't understand the axes here? The x axis is the pairwise comparisons... 18 sites = 153 pairwise comparisons. The y axis is the number of DEGs per comparison? And then it is ordered by that value? So the number of genes in the text part of the figure is generated elsewhere? Because the DEGs in each column are not necessarily all different?

Figure 3c: I would instead order by % natural area as I think it will highlight your main point more.

Methods
Line 540-541, lines 544, line 579: Some information on handling times between being caught and the tissue being put in RNAlater would be warranted. Also information on how long samples were at room temperature, 4C, and 20C (or whatever).
Line 590: I'd flip "quality and concentration" since the Qubit was for concentration and the TapeStation was for quality.
Line 594: Somewhere there should be the (average and sd) total number of reads per sample.

(Remarks on code availability)

Reviewer #2

(Remarks to the Author)
Review of Manuscript: Decoding the constraints acting on a coastal fish using landscape transcriptomics

General Assessment (Major revisions)

This manuscript presents an ambitious and potentially high-impact study combining (i) field sampling across a coastal urbanisation gradient (18 sites; juveniles and adults; whole-body vs liver) with (ii) controlled feeding experiments, and (iii) transcriptomic analyses aimed at extracting interpretable signatures of environmental change. The dataset is unusually rich for a field transcriptomics study, and several elements are particularly promising: the life-stage/tissue contrast, the semi-randomised sampling logic to cope with multi-factor heterogeneity, and the attempt to translate complex multi-gene responses into interpretable summaries (e.g., radar-plot framework).

However, major revisions are needed before the manuscript is suitable for publication. As currently framed, the study reads like two narratives that are not yet fully integrated: (i) a landscape transcriptomics proof-of-concept linking gene expression to "human activities," and (ii) a feeding experiment used to interpret field transcriptomes via a "fast-food" narrative. This weakens conceptual focus and makes several conclusions harder to evaluate.

In my view, the strongest and most testable contribution of the dataset is the hypothesis that urban-influenced habitats provide increased (and/or altered) food resources that incentivise habitat use despite suboptimal conditions, resulting in a trade-off between energetic status and chronic physiological stress detectable in transcriptomic signatures. I therefore recommend a major refocus and re-organisation of the manuscript around this single central hypothesis. If the authors adopt this reframing, the title and abstract should also be revised to foreground the food–stress trade-off and the field–experiment integration rather than a broad "constraints/landscape transcriptomics" framing.

Key priorities for revision (high-level):
1. Unify the manuscript around one explicit, hypothesis-driven story (urban resource subsidy vs chronic stress trade-off).
2. Define and visualise the environmental/urban gradient transparently (and test confounding/collinearity).
3. Tighten interpretation of the feeding experiment (what is demonstrated vs what is inferred).
4. Strengthen methods transparency and reproducibility for land-cover linkage and DE/omics workflows.

Major Comments

1. Conceptual focus: unify the manuscript around a single, hypothesis-driven story
Essential to address for publication: At present, the objectives oscillate between "testing landscape transcriptomics as a monitoring tool" and "testing an urban resource–stress trade-off." The latter appears to be the most novel and compelling contribution, and the one the data can most directly support if framed carefully.

Actionable suggestions:
• State the primary hypothesis explicitly (urban resource subsidy → higher feeding/energetic status, coupled with chronic stress signatures), with clear, testable predictions for juveniles vs adults (and tissue-specific expectations).
• Restructure the Results to follow a single causal/interpretive logic: environmental gradient → fish status/phenotype (feeding/stress proxies) → transcriptomic patterns → experimental anchoring.

• If the manuscript is reframed this way, revise title/abstract/Introduction accordingly (and avoid presenting the work primarily as a general “landscape transcriptomics proof-of-concept”).
Optional but would strengthen: Briefly position this within the broader urban ecology/urban evolution trade-off framing (resource subsidies vs suboptimal conditions/chronic stress), as it aligns strongly with the story the manuscript develops.

2. Establish a clear “urban stress” gradient and use the radar framework as the bridge
Essential to address for publication: A major reason the story feels fragmented is not the lack of analyses, but the order in which they are presented. The reader does not see an early, quantitative bridge between: (i) environmental/urban variables, (ii) fish status (feeding condition / stress proxies), and (iii) genome-wide expression patterns. Your radar-plot framework is well-suited to serve as this bridge, but it currently appears later than would be optimal for guiding interpretation. In particular, the radar-plot summary is a strong candidate for this integrative ‘bridge’ and would be most effective if introduced immediately after the site-gradient definition.

Actionable suggestions:
• Define the environmental gradient transparently first. Add a multivariate characterisation/ordination of sites based on the full measured environmental dataset (not only land cover), and use this to define a defensible “urban/environmental” axis (even if composite).
• Test confounding explicitly. Report whether land cover covaries with temperature, salinity, chlorophyll-α, sampling time, etc., and show how this is addressed (model structure, sensitivity/supplementary analyses).
• Move the “bridge” analysis earlier and make it a signpost. Introduce fish “status” metrics (feeding-status proxy; stress-related signatures) and the radar-plot summaries early in the Results, mapped onto the environmental gradient. This will help the reader understand the proposed “urbanisation → feeding/stress axis” before the more detailed gene-by-gene or pathway-level transcriptomic interpretation.

Would strengthen (but consistent with your current approach): Keep the radar framework, but state explicitly what it is doing: a hypothesis-guided diagnostic summary to integrate signals (rather than a purely exploratory visual). Then refer back to it consistently when interpreting subsequent transcriptomic patterns (e.g., “sites with high feeding index but elevated stress radar scores show…”).

3. Feeding experiment interpretation: tighten what is demonstrated vs hypothesised
Essential to address for publication: The feeding experiment is valuable and is a key strength of the paper. However, some manuscript language implies stronger mechanistic specificity (e.g., “fast-food / high-lipid” diets) than is directly shown by the feeding manipulation and field data.
Actionable suggestions:

• Clearly state what the feeding experiment manipulates (fed vs unfed; duration; diet type), and what it can and cannot infer about “excess,” “diet quality,” or “high-lipid” urban diets.
• If the “fast-food/high-lipid” framing is retained, provide supporting evidence/analyses (diet composition proxies, stomach contents, lipid metrics, stable isotopes, prey-field differences, or clearly justified external datasets). Otherwise, temper wording so the conclusion is framed as a resource subsidy (higher feeding status) paired with stress/inflammation signatures, without specifying diet quality/composition.
4. Definition of “human activities” signal and confounding with other drivers
Essential to address for publication: The manuscript reports “genes uniquely linked to human activities,” yet temperature, salinity, sampling time, and other variables strongly influence gene expression. Without a transparent operational definition and clear handling of collinearity, attribution to “human activities” is difficult to evaluate.

Actionable suggestions:
• Provide a clear operational definition for “uniquely linked to human activities” (model form, thresholds, multiple testing, and assignment rules).
• Show diagnostics: correlation among predictors, sensitivity to influential sites (not only temperature extremes), and robustness to alternative definitions of the “urban” axis.
• Ensure site-level environmental structure is carried consistently into downstream analyses and figure colouring/grouping.

5. Methods clarity and reproducibility: strengthen key details
Essential to address for publication: Several methodological components are central to the conclusions but are currently underspecified or potentially confusing.
Actionable suggestions:

• Land cover linkage: Clarify what “land cover within 1 km” means for a marine sampling point (buffer definition, spatial workflow/software), and justify why this is the appropriate terrestrial linkage (vs catchment-based influence/discharge).
• Use of additional field variables: Community/substrate/turbidity/fish density appear collected but their role in the main models is unclear. If they were tested and excluded, say so (and why). If not used, streamline or clarify their purpose.
• DE workflow clarity: Explicitly describe the DESeq2 contrast strategy (pairwise site contrasts vs reference-site strategy), whether “$p < 0.01$” refers to adjusted p-values (FDR) or raw p-values, and how the many tests are interpreted/aggregated.
• Reference genome vs transcriptome ambiguity: Specify exactly what “reference transcriptome” is (de novo assembly vs transcript models from the genome), including accession/version and how the Kallisto index was built. Because Kallisto yields transcript-level estimates, explicitly describe how transcript abundances were aggregated to gene-level counts for DESeq2 (e.g., tximport and rules for isoforms). Also, describing Kallisto as “aligned” is misleading; revise terminology.
• QC reporting: Provide explicit RNA integrity and QC summaries (TapeStation DIN/RIN-equivalent values, read depth per

sample, mapping rates already given, and any filtering thresholds).

Optional but would strengthen: The population genetics (RNA-seq SNP calling; DAPC) is potentially useful to rule out population structure confounding, but it currently feels disconnected. Add a short statement in Results/Discussion with the take-home message and point to the Extended Data figure, or move it fully to Supplementary Methods.

Detailed Comments (by line)

Framing, literature, and hypothesis clarity

(Lines 26–27) The citations motivating multi-stressor frameworks (e.g., Gissi; Pirotta) feel somewhat dated given how quickly this area is moving. Please consider updating the Introduction with more recent syntheses and/or field-oriented multi-stressor frameworks (e.g., https://doi.org/10.1002/ece3.71959 and additional recent literature), so the manuscript is anchored in the current state of the field.

(Lines 34–36; 40–42) The Introduction/Discussion would benefit from explicitly framing the work within an urban ecology / urban evolution trade-off perspective (organisms exploiting urban resource subsidies while tolerating suboptimal conditions and chronic stress). This framing is closely aligned with your later results/interpretation (feeding status vs stress trade-offs). A useful entry point is the "urban evolution" framework paper (DOI: 10.1126/science.aam8327).

(Line 48) When discussing the conceptual shift from single-stressor laboratory exposures to multi-stressor real-world contexts (and how omics can help bridge these), it would strengthen the narrative to cite foundational perspectives alongside omics-era discussion. Suggested references: 10.1016/j.aquatox.2003.11.011; 10.1016/j.cbd.2016.06.010.

(Lines 62–63) Please strengthen contextualisation of environmental/ecological transcriptomics in fish, as this is central to the manuscript's positioning. Suggested citations: 10.1242/jeb.148833; 10.1139/facets-2022-0177; 10.1016/j.cbpb.2021.110629.

(Line 64) The aim is clear here, but the manuscript's main "take-home" shifts later. Please clarify early what the primary hypothesis is (monitoring proof-of-concept vs urban resource–stress trade-off) and keep the narrative consistent throughout.

(Line 92) A few statements read like results or interpretations appearing in the Introduction. Please keep the Introduction focused on background, rationale, and hypotheses, and relocate result-like statements to the Results/Discussion sections.

Differential expression strategy and definition of "urban signal"

(Lines 97–98) The hypothesis is currently presented very broadly ("gene expression shifts along the gradient"), but later the manuscript interprets patterns in specific functional directions stress/inflammation; food/energetics). Consider sharpening the hypothesis and predictions up front, ideally grounded in prior environmental transcriptomics studies across anthropogenic environmental gradients (e.g., 10.1016/j.scitotenv.2024.173656; 10.1016/j.envpol.2024.124400).

Relatedly, clarify how any targeted gene sets/panels are defined to avoid the perception of post hoc selection (clearly distinguish hypothesis-driven panels from exploratory outputs).

(Lines 104–108) The DESeq2 field-comparison approach needs a clearer explanation. Please explicitly state: (i) the design formula used, (ii) whether all pairwise site contrasts were performed or a reference-site framework was used, (iii) how many contrasts/tests were run, (iv) whether the stated threshold ("$p < 0.01$") is adjusted p-values (FDR) or raw p-values, and (v) how these outputs are used downstream (e.g., descriptive "transcriptomic distance," frequent-DE genes, site grouping). As written, the workflow is difficult to reproduce and the large DEG counts may be misinterpreted without these details.

Environmental gradient, confounding, and "human activities" attribution

(Lines 136–138) Because temperature has a strong footprint, please explicitly address whether "urbanisation" covaries with temperature (and/or salinity, chlorophyll-α). If more urbanised sites are systematically warmer (etc.), show how this is handled analytically.

(Lines 140–146) When identifying "425 genes (juveniles) / 585 genes (adult liver) uniquely linked to human activities," please define "uniquely linked" operationally (models used, thresholds, handling of collinearity, and assignment rule). Without this, the strength of the attribution to human influence is hard to evaluate.

(Lines 146–150) I strongly recommend adding an environmental ordination/clustering (PCA/NMDS or similar) using the full environmental dataset. This would allow the reader to see whether "urbanisation" is a composite of correlated stressors and would better justify downstream grouping/colouring/interpretation.

Integration of field + experiment: strengthen the causal story

(Lines 174–178) The manuscript would benefit from explicitly stating (as hypothesis/prediction) whether the central aim is (i) "gene expression varies along land cover/human footprint," or (ii) "urban habitats provide increased food resources and fish trade off energetic gains against chronic stress." The current integration can read as a post hoc linkage between two strong

datasets.

(Lines 190–196) The strongest contribution, in my view, is the integration of field transcriptomes with a controlled feeding experiment to interpret field patterns in terms of energetic state and stress trade-offs. I recommend foregrounding this contribution throughout (title, objectives, Results structure), rather than presenting the work primarily as a general landscape transcriptomics demonstration.

(Lines 197–205) The metatranscriptomic diet inference is only presented for juveniles. Please clarify early why this is restricted to juveniles (whole-body data), and strengthen interpretation by addressing: (i) do these diet signatures vary systematically along the urban gradient, and (ii) what is the plausible mechanism of food increase in urban settings (direct subsidies vs nutrient-driven shifts in prey biomass/community composition)? Even if adults cannot be analysed similarly, avoid overgeneralising.

(Lines 235–239) This analysis appears to be the “missing link” that could make the manuscript’s logic much clearer. I suggest introducing (or moving) this earlier, immediately after defining the environmental/urban gradient—so fish condition/feeding status and stress signals are mapped onto the gradient before the untargeted transcriptome interpretation. This re-organisation would substantially improve readability and strengthen the causal narrative.

Discussion scope

(Lines 323–327) The Discussion expands into pollution/toxicant framing, but chemical pollution is not directly measured or presented (as currently visible to the reader). I suggest narrowing the Discussion to what is supported by the measured variables and analyses, unless external pollution datasets are integrated explicitly and transparently. A tighter scope focused on the urban food–stress trade-off would increase clarity and impact.

(Lines 340–346) The manuscript currently reads as two separate experiments connected by interpretation rather than by a single pre-stated hypothesis. A major revision should aim to unify the narrative: define the urban gradient, demonstrate its relationship with fish status/stress, interpret transcriptome patterns along that axis, and use the feeding experiment to anchor mechanistic interpretation.

Methods transparency and reproducibility (additional specifics)

(Lines 506–507) Fish community data are mentioned but their analytical role is unclear. Please specify how community metrics were used (or, if not used, clarify why they were collected and where they are reported).

(Lines 517–518) Land-cover methodology needs to be reproducible and conceptually appropriate for marine sampling points. Please clarify software/workflow, spatial unit (buffer), and how terrestrial land cover is linked to each marine site (shoreline buffer vs catchment influence).
(Lines 519–521) Several measured variables (substrate, fish density, turbidity, diversity metrics) do not appear in the key models. Clarify whether they were tested/excluded and provide a concise overview of variable selection.

(Line 573) The feeding experiment contrasts “fed (normal food amounts)” vs “fasted,” which supports interpretation in terms of feeding status/energetic state. It does not directly test “excess” or “low-quality/high-lipid” urban diets unless diet composition/quality is measured. Please separate what is demonstrated from what is hypothesised, and temper wording accordingly if needed.

(Lines 596–598) Please report standard RNA quality metrics (e.g., TapeStation DIN/RIN-equivalent values) and key QC statistics, not only general statements about checks performed.

(Line 599) Please clarify what “reference transcriptome” was used, provide accession/version, and describe gene-level aggregation from Kallisto outputs for DESeq2. Also replace “aligned” with accurate terminology for Kallisto-based quantification.

(Lines 678–690) If population structure analyses are included to rule out confounding, state the purpose and take-home message in the main text (with a pointer to the Extended Data figure), or move to Supplementary Methods to avoid disconnection.

Conclusion and Recommendation

This manuscript presents a substantial field and experimental effort, and the authors have assembled a rich, multi-level dataset with clear potential for a strong contribution. In particular, the combination of landscape transcriptomics with controlled feeding experiments is innovative, and the effort to translate multi-gene signals into interpretable summaries is promising.

That said, the manuscript currently requires major revision before it can be considered for publication. The central limitation is not the amount of data, but the conceptual focus and integration: the field and experimental components read as partially disconnected, and several key interpretations (especially regarding “human activities” attribution and diet-quality mechanisms) are not yet supported with sufficient analytical transparency and framing. Strengthening the definition of the environmental/urban gradient, clarifying how “human-activity–linked” signals are derived, tightening claims to what is directly

demonstrated, and reorganising around a single hypothesis-driven narrative would substantially improve interpretability.

I therefore recommend major revisions. With a clearer hypothesis, a coherent analytical flow (environmental gradient → fish status/stress → transcriptome patterns → experimental anchoring), and strengthened methods transparency, I believe this study could become a compelling and well-supported manuscript.

Disclaimer: I used a large language model (LLM) to assist in drafting and editing this peer-review report (improving structure, clarity, and wording). The scientific assessment, interpretation, and final recommendations are my own, and I take full responsibility for the content of this report.

(Remarks on code availability)

Version 2:

Reviewer comments:

Reviewer #1

(Remarks to the Author)
I previously reviewed this manuscript and I think the authors did an excellent job responding to my suggestions as well as those of the other reviewer. The dramatic reorganization they did in response to the other reviewer really improves the overall impact of the paper. It feels more hypothesis driven rather than technique driven now.

I have some feedback, but my purpose is to give constructive criticism. I want to emphasize that overall I think this is an important contribution that goes well beyond prior work on urbanization effects by leveraging transcriptomics in creative ways as a window into physiological effects of anthropogenic stressors on wild free-living fish across a large geographic range (along the coast of the island of Okinawa). Not only is it an important test case, but the results have important implications for our understanding of anthropogenic effects on animals in the short and possibly long term. The paper is very well written (often challenging with this kind of data), leverages a combination of field-based, experimental, and literature mining data to form conclusions. The results highlight the complex trade-offs that come with environments closer to human disturbance – fish appear well fed but suffer other stress effects according to their transcriptomes.

Some main points of feedback:
1. With the new reorganization, the radar plots come early, and while I do think this is the right place for them, the fact that these are presented as qualitative analyses gave the paper a bit of a story-telling vibe to me. I think the authors do a good job of highlighting certain sites as examples to underline their points, but I wonder if there is a quantitative method that could be developed to judge these comparisons. For example, perhaps something borrowed from geometric morphometrics to ask how similar two radar plots are in shape? Presumably a null distribution would be a circle traversing the middle of the radar plot… could that fact be leveraged somehow?
2. The authors did a good job in their revision of better explaining how these radar plots are constructed. This is a bit of a quibble, but when they say that “The first eigenvector of the covariance matrix of the average site coordinates was used to define a main axis of site-wise variance across the retained PC dimensions.” this is the same as the PC1 from a PCA of the average site coordinates.
3. I am still not a fan of the WGCNA analysis. It is done on a very small subset of curated genes and the authors of WGCNA have made it very clear back when they developed the software that they do not think that’s an appropriate use. Also I’m not sure what it gains. From what I can see in the Extended Figure 7, the modules are difficult to interpret (span multiple categories). I would suggest instead taking each category of genes, taking the PC1 of the expression of genes within each and using those PC1 scores instead of scores from the eigengenes of constructed modules. Further exploration of the overlap in expression between these categories of genes could be done, for example with a correlation matrix.
4. The Discussion still feels a little lopsided, emphasizing landscape transcriptomics more relative to the implications of the results themselves.

More minor points (line number refer to the version without tracked changes):
Line 110: Why is variant calling just with juveniles, and is it based on the transcriptomes or other genomic data?
Figure 1f: I was curious what the null distribution for this would look like – it could be derived through simulation by permuting the existing data.
Line 256: I think I’d still refer to them as “radar plots” instead of “radars”
Line 280: Be careful with words like “modulate” that imply a cause-and-effect in a paper where the data are correlational.
Line 318: Change “to” to “with”
Line 352: Insert “but” before “with”
Line 354: Why say “partially buffers”? I thought these fish were well-fed but more stressed?
Line 392: There is a study in bumble bees that might be appropriate to cite here: DOI: 10.1111/mec.17626
Figure 2 legend: “The first eigenvector of the average coordinates from each site” should say “first eigenvector of the covariance matrix of the average coordinates” according to your methods.
Supplemental Table 10: I missed what this was for?
Extended Data Figure 2: Minor typo – there are 8 PCs for adult liver and 6 for juvenile bodies, not 6 for each. Also can you clarify – is each column a single linear model?

In conclusion, I commend the authors on an excellent study and a very well written paper that was an enjoyable and interesting read.

(Remarks on code availability)

Reviewer #2

(Remarks to the Author)
I thank the authors for the thorough and thoughtful revision. The manuscript has improved substantially, and I appreciate the clear effort invested in addressing the conceptual, analytical, and methodological points raised in the first review.

In particular, the revised version is now much more coherent and hypothesis-driven. The manuscript successfully clarifies the central narrative and improves the integration between the landscape transcriptomics component and the feeding experiment. The strengthened definition and visualisation of the environmental/urban gradient, together with clearer treatment of potential confounding among environmental drivers, greatly improve interpretability and make the main conclusions more robust. I also commend the improved methodological transparency in the omics workflow and land-cover linkage, which strengthens reproducibility and reader confidence.

Overall, the revision has addressed my major concerns, and the manuscript now reads as a mature, well-supported contribution. I congratulate the authors on the quality of the work and the care taken in the revision.

Recommendation: I support publication of this manuscript in its current form (acceptance).

(Remarks on code availability)

Version 3:

Reviewer comments:

Reviewer #1

(Remarks to the Author)
I thank the authors for addressing my concerns. I highly recommend the publication of this manuscript. I congratulate the authors on a job very well done!

I do have a couple minor questions/comments regarding the Extended Data Figures that I hope will be useful.
Extended Data Fig. 2: Could you further clarify whether all variables in the table were included in models for each PC? In other words, was the same model run for each PC? Or could blanks mean that the variable was not included because of BIC criteria?
Extended Data Fig. 3: The y-axis of c&d is log-scale, but the plotted variable is still the number of DEGs. So the y-axis title should still be simply “Number of DEGs”. Also in the title of the caption, should “with” be “within”?
Extended Data Fig. 5: Why is site M missing from part b? Also, a similar question as before; were all variables in this chart included or do blanks mean they weren't?
Extended Data Fig. 12: Missing text on x axis.

(Remarks on code availability)

**Open Access** This Peer Review File is licensed under a Creative Commons Attribution 4.0 International License, which permits use, sharing, adaptation, distribution and reproduction in any medium or format, as long as you give appropriate credit to the original author(s) and the source, provide a link to the Creative Commons license, and indicate if changes were made.
In cases where reviewers are anonymous, credit should be given to 'Anonymous Referee' and the source.
The images or other third party material in this Peer Review File are included in the article's Creative Commons license, unless indicated otherwise in a credit line to the material. If material is not included in the article's Creative Commons license and your intended use is not permitted by statutory regulation or exceeds the permitted use, you will need to obtain permission directly from the copyright holder.
To view a copy of this license, visit https://creativecommons.org/licenses/by/4.0/

**Reviewer #1**

This was an ambitious and well-done study that truly shows the potential of landscape transcriptomics to inform how wild organisms respond to complex sets of environmental stressors. It uses a nice combination of field-collected samples across environmental gradients, lab-based controlled experiments, and data mining of the literature to come to the interesting conclusion that damselfish near urban centers are highly stressed but overfed, which the authors compare colorfully to living on a "fast-food diet". In addition, the development of radar plots as a visualization and quantification tool for assessing stressors at different sites has great potential as a practical conservation and management tool. I found enjoyable to read which is often a feat for transcriptomics papers.
**Answer: We sincerely thank the reviewer for this generous and encouraging assessment of our work. We greatly appreciate the recognition of the ambition of the study, the integration of field sampling, controlled experiments, and literature-based interpretation, as well as the positive feedback on the conceptual framing and readability of the manuscript. In response to the detailed comments provided, we have carefully revised the text to clarify our conclusions, moderate certain interpretations, and further strengthen the integration between datasets. We have also refined the presentation of the radar-plot framework to better highlight its analytical rigour and potential as a conservation and management tool. We hope that the revised version remains enjoyable to read while offering a clearer and more robust contribution.**

I have a couple areas for improvement I'd like to highlight.

First, while I strongly agree that the radar plots represent an advance in leveraging transcriptomes to identify environmental stressors, how they were created wasn't well described and I found the limited descriptions in the paper and methods confusing (and I like to think that I am quite adept at multivariate statistics and linear algebra). In fact, it was the supplemental figure that best illustrated what was going on. I'd suggest including some aspects of that figure in the main paper and also revisiting the wording. For example, consider clarifying that the axis of maximum variance is in fact a multivariate axis of maximum variance (if I'm understanding correctly). Also importantly so that others can actually utilize this contribution, how is that step done? For example, in the supplementary figure, it says "The direction of the main variance in the mean coordinate of the sites is then calculated, here presented as a dashed axis," but this is rather vague. Are you running a new PCA on your (reduced number of) PCs? Or is it some kind of factor rotation? This detail is very important for future adoption.
**Answer: We re-phrased the methods section as follows (L 867-870): "*A Principal Component Analysis (PCA) was performed for each set of genes. The number of PC dimensions accounting for 50% or more of the variance was obtained; this threshold was used to capture a consistent proportion of the variance across the gene sets. Using these PC dimensions, the average coordinates of the samples from each site were calculated. The first eigenvector of the covariance matrix of the average site coordinates was used to define a main axis of site-wise variance across the retained PC dimensions. Each sample was projected along this axis, yielding a segment bounded by the two most extreme samples. The projection of each sample along this axis was scaled to obtain one value per sample ranging from 0 to 1. The assignment of the values depended on the average expression in the two most extreme sites along the axis (sample with a value of 0 close to the most extreme site showing lower expression, and 1 close to the opposite most extreme site that had higher expression).*"**

**To directly answer the reviewer, we are not running a new PCA on the reduced PCs and do not apply factor rotation.**
**In line with the reviewer's suggestion, we thought it was important to provide more details on the radar plot methodology and this is why, since submitting this manuscript, we developed an R package and have written an accompanying article going through the steps and illustrated with case**

**studies. Please find the preprint at [https://doi.org/10.64898/2026.01.14.699593](https://doi.org/10.64898/2026.01.14.699593) and underlying code at [https://github.com/Emma-Gairin/RadarOmics](https://github.com/Emma-Gairin/RadarOmics)**
**We now added a reference to this preprint in the materials and methods. Note however that this package is not necessary to produce the analyses using the methods described here and the code accompanying the manuscript.**

Second, I can't figure out at all how WGCNA was utilized in this study. Was it another analysis that just wasn't featured in the main text? I do commend you on using linear models in the supplement and not relying on the standard and very limited correlation table approach many other people still use.
**Answer: WGCNA was used for Extended Data Fig. 7 as part of a hypothesis-driven clustering approach to group together genes that are typical markers of stress (immune response, xenobiotic metabolism, heat shock, etc.) based on shared responses across all samples. The first component of the expression of these clusters across the samples was analysed with linear mixed-effect models to detect the contribution of different fish characteristics or environmental parameters.**

Third, I was unclear on why a secondary PCA had to be done for the analyses comparing experimental food deprivation to wild-caught samples. If you know the loadings then you could just apply those to the wild-caught gene count matrix (it's simple matrix algebra).
**Answer: This comment appears to assume that the secondary PCA was obtained by projecting wild-caught samples using the gene loadings derived from the experimental dataset. This is not what we did here and we apologize for not having been clear enough on this point. Instead, we used the experimental dataset to select the genes with highest loadings on PC1. A new PCA was then performed on all samples using this subset of genes. In other words, the gene loadings from the experimental PCA were not applied or propagated into the secondary PCA analysis. We clarified our method section (L 850-856): "*Fasting-feeding experiments were used to derive the feeding status of the field fish. To do so, PCAs were performed on the expression level of all genes of the juvenile whole-body and adult livers following the feeding experiment. The top 10% of genes with highest loading along PC1 were identified. The expression level of these genes in both the feeding and field samples was used for secondary PCAs for the juvenile whole-body and adult liver samples. In these secondary PCAs, the field samples fell between the feeding samples along PC1. Thus, the PC1 coordinate of each sample was taken as an indicator or proxy of each sample's feeding status*"**

More minor or specific comments:

Abstract
Lines 32-34: "We uncover a striking molecular signature of urbanization: fish from human-impacted sites exhibit elevated expression of genes involved in inflammation and immune responses." I'd delete this sentence. It is repetitive with the sentences that follow and I found it made this part of the abstract confusing.
**Answer: The abstract was modified to match the narrative reframing suggested by reviewer #2. This sentence was therefore deleted.**

Main
Lines 68-70: "after a period of larval dispersal in the open ocean, different species may prefer specific habitat features – such as higher nutrient availability or stable temperatures – when settling on a reef." I don't see how this fits here, consider deleting or moving. I found it distracting.
**Answer: As suggested, this sentence was removed.**

Lines 110-112: "As only one organ was considered for adults, the number of genes and range of physiological responses expressed in the samples was lower than for juvenile whole-body samples."

I'm not sure this had to be the case, however. Often whole body or tissue sampling is expected to obscure tissue-level or cell-level differences, respectively.
**Answer: We removed this sentence as requested. The total number of genes expressed in the liver samples is much lower than in the juvenile whole-body samples, but we agree that the physiological responses may be clearer in the focused liver transcriptomes.**

Line 130: You could statistically test whether there was clustering by sites as seems apparent from the figure (e.g., PERMANOVA).
**Answer: To take into account this comment, we added the following precisions (L 115-116): "*samples from the same sites clustered closely (significant signature of site on PC1/2, PERMANOVA, $R^2$ = 0.76, p = 0.001 for juveniles, $R^2$ = 0.67, p = 0.001 for adult livers)*."**

Line 132: Did the quadratic model also have a linear component?
**Answer: In Dromics, for each gene, both a simple linear and a model that includes both a linear and quadratic term are used. The best fitting model is used to determine if the gene displays a monotonic or biphasic response. In Fig. 4 we grouped together genes that have a linear and quadratic response to focus, at a higher level, on which genes are sensitive to given parameters. We added more precision to the methods section (L 804-807), "*Using DRomics 2.6-2, the genes responding linearly or quadratically to various environmental factors (percentage of natural zones near each site, temperature, sampling time, salinity, chlorophyll-α) and to size (for juvenile fish) were obtained at a significance threshold of adjusted p < 0.01. We grouped together genes that have a linear and quadratic response to focus on which genes are sensitive to given parameters in a monotonic or biphasic manner*."**

Line 142-144: I am having trouble picturing what is actually being compared to get this statistic?
**Answer: We apologize for the lack of clarity. We tried to improve the phrasing (L 310-313): "*The proportion of genes responding to each factor was significantly similar in juvenile whole-body and adult liver samples, suggesting strikingly parallel molecular programs across tissues and developmental stages (Pearson's Chi-squared test, $\chi^2$=3548, df=19, p<$10^{-15}$)*."**

Line 146: What does "multiple biological levels" refer to? Cells, tissues, organs? What is this statement based on?
**Answer: We removed this part of the sentence and now state (L 321-322) "*These conveyed generalised stress with modifications of the expression level of genes*".**

Line 155: "do not respond to other environmental or organismal factors" clarify that this statement is based on your data.
**Answer: We apologize for the lack of clarity. We added a clause referring to the statistical model used to conclude that the genes can be used as markers of stress (L 328-331): "*Because these genes did not respond linearly or quadratically to other environmental or organismal factors, they may represent diagnostic markers of human-related stress and convey mechanisms used by fish to survive in urban environments*."**

Figure 1
b: Density here refers to density of points in the figure?
**Answer: Sorry for the lack of precision. Yes, it is the density of points. The figure legend of panel e (previously panel b) was updated to reflect this on the Figure itself and in the text legend (L 142-143): "*The density of points (one point per pairwise comparison) is represented in gradual grey-blue shading*"**

c: I don't understand the axes here? The x axis is the pairwise comparisons... 18 sites = 153 pairwise

comparisons. The y axis is the number of DEGs per comparison? And then it is ordered by that value? So the number of genes in the text part of the figure is generated elsewhere? Because the DEGs in each column are not necessarily all different?

**Answer: We effectively realized this was not clear. Based on this comment, we clarified the figure legend and caption of panel f (previously panel c). In particular, we modified the title of the axes to indicate that x is the number of occurrences of a given DEG in the pairwise site comparisons (if the gene has a very site-specific behaviour and is significantly different between all sites, it would appear 153 times) and y is the number of DEGs with a given occurrence. The DEGs in each column are distinct (the sum of all columns equates to the total number of genes in juvenile or liver samples).**

**This graph aims to show that most genes differ in expression only in a few pairwise comparisons.**

**The number of genes presented in the text corresponds to panel e ("88 to 6,348 genes significantly differed in expression level in pairwise site comparisons (*e.g.,* site A *vs.* B) for juvenile whole-body samples, and 15 to 3,763 for adult liver samples") or panel f ("(2,410 genes in >25% of the pairwise site comparisons while adults—which have resided on their sites for months to years—showed site-specific profiles (251 genes retrieved in >25% comparisons)"). This number is the sum of the number of DEGs present in more than 39 pairwise comparisons (153 / 4 = 38.25) which corresponds to the size of the columns to the right of the dashed line. We updated the legend of panel f to improve clarity (L 145-150): "*Number of pairwise site comparisons where a differentially expressed gene occurs (as n=18 sites were compared, a gene with a unique signature in all sites would occur in n(n-1)/2=153 pairwise site comparisons). A vertical dashed line corresponding to 39 pairwise comparisons (i.e. 25% of all comparisons) is used to illustrate the high site specificity of gene signatures in adult livers (251 genes in more than 39 pairwise comparisons) compared to whole-body juveniles (2,410 genes).*"**

Figure 3c: I would instead order by % natural area as I think it will highlight your main point more.

**Answer: This is a good idea, thank you. We reordered the spectral plots by % of natural area (from least to most natural) on Figure 3 and updated the legend of panel d accordingly (L 253): "*sites presented based on the percentage of natural zones within a kilometre*".**

Methods

Line 540-541, lines 544, line 579: Some information on handling times between being caught and the tissue being put in RNAlater would be warranted. Also, information on how long samples were at room temperature, 4C, and 20C (or whatever).

**Answer: To take into account this comment, which we agree with, we now state (L 699-702): "*In total, 90 juveniles, 49 females, and 40 males were collected and brought back to shore in transparent plastic bags allowing water flow. The handling time between sampling and euthanasia varied between 5 and 20 minutes.*" and *"All RNAlater-preserved samples were kept on ice for transport, placed at 4°C for 24 hours, and stored at -20°C until extraction."* (L706-708).**

Line 590: I'd flip "quality and concentration" since the Qubit was for concentration and the TapeStation was for quality.

**Answer: This is correct, and we inverted the two words (L 759).**

Line 594: Somewhere there should be the (average and sd) total number of reads per sample.

**Answer: The reviewer is correct. We now provide the number of reads per sample in the materials and methods (L 764-767): "*The average number of paired-end reads was 72.2 ± 6.5 million for the juvenile and adult field samples, 68.0 ± 6.0 million for feeding experiment juvenile whole-body samples, and 80.9 ± 9.3 million for feeding experiment adult liver samples*.".**

**Reviewer #2**
Review of Manuscript: Decoding the constraints acting on a coastal fish using landscape transcriptomics

General Assessment (Major revisions)
This manuscript presents an ambitious and potentially high-impact study combining (i) field sampling across a coastal urbanisation gradient (18 sites; juveniles and adults; whole-body vs liver) with (ii) controlled feeding experiments, and (iii) transcriptomic analyses aimed at extracting interpretable signatures of environmental change. The dataset is unusually rich for a field transcriptomics study, and several elements are particularly promising: the life-stage/tissue contrast, the semi-randomised sampling logic to cope with multi-factor heterogeneity, and the attempt to translate complex multi-gene responses into interpretable summaries (e.g., radar-plot framework).
However, major revisions are needed before the manuscript is suitable for publication. As currently framed, the study reads like two narratives that are not yet fully integrated: (i) a landscape transcriptomics proof-of-concept linking gene expression to "human activities," and (ii) a feeding experiment used to interpret field transcriptomes via a "fast-food" narrative. This weakens conceptual focus and makes several conclusions harder to evaluate.
In my view, the strongest and most testable contribution of the dataset is the hypothesis that urban-influenced habitats provide increased (and/or altered) food resources that incentivise habitat use despite suboptimal conditions, resulting in a trade-off between energetic status and chronic physiological stress detectable in transcriptomic signatures. I therefore recommend a major refocus and re-organisation of the manuscript around this single central hypothesis. If the authors adopt this reframing, the title and abstract should also be revised to foreground the food–stress trade-off and the field–experiment integration rather than a broad "constraints/landscape transcriptomics" framing.
**Answer: We sincerely thank the reviewer for this constructive and encouraging evaluation. Following this recommendation, we have thoroughly reframed the manuscript around a single central hypothesis: that urban-influenced habitats provide altered or increased food resources that may promote habitat use despite suboptimal conditions, leading to a trade-off between energetic status and chronic physiological stress detectable in transcriptomic signatures. The narrative has been reorganized to better integrate field data and feeding experiments within this unified framework, and the title and abstract have been revised accordingly. We have also carefully addressed all technical and conceptual comments, clarified methods, refined interpretations, and adjusted claims where necessary. We hope that this revised version is now clearer, more coherent, and meets the reviewer's expectations. Below we provide detailed answers to each point.**

Key priorities for revision (high-level):
1. Unify the manuscript around one explicit, hypothesis-driven story (urban resource subsidy vs chronic stress trade-off).
2. Define and visualise the environmental/urban gradient transparently (and test confounding/collinearity).
3. Tighten interpretation of the feeding experiment (what is demonstrated vs what is inferred).
4. Strengthen methods transparency and reproducibility for land-cover linkage and DE/omics workflows.
**Answer: Thank you for these constructive comments. This was effectively an ongoing discussion in the lab on how to present these results. Therefore, we addressed each actionable suggestion provided by the reviewer below and we have extensively reorganized the manuscript.**

Major Comments

1. *Conceptual focus: unify the manuscript around a single, hypothesis-driven story*

Essential to address for publication: At present, the objectives oscillate between "testing landscape transcriptomics as a monitoring tool" and "testing an urban resource–stress trade-off." The latter appears to be the most novel and compelling contribution, and the one the data can most directly support if framed carefully.
**Answer: We have done our best to revise the manuscript and make this second narrative more obvious.**

Actionable suggestions:

• State the primary hypothesis explicitly (urban resource subsidy → higher feeding/energetic status, coupled with chronic stress signatures), with clear, testable predictions for juveniles vs adults (and tissue-specific expectations).
**Answer: We now state (L 71-76): *"We hypothesised that juvenile and adult C. cyanea integrate multiple and interacting environmental factors into coordinated shifts in gene expression, and that these can reveal physiological trade-offs involved in urban life that remain invisible to classical ecological monitoring. Specifically, we predicted that human activities would lead to a signature of chronic stress and altered energy metabolism, a pattern reported in terrestrial urban wildlife*".**

• Restructure the Results to follow a single causal/interpretive logic: environmental gradient → fish status/phenotype (feeding/stress proxies) → transcriptomic patterns → experimental anchoring.
**Answer: We modified the order of the results. We now start from the environmental characteristics and general overview of the transcriptomes, then go over the radar plots showing general patterns across processes, link these patterns with feeding status, and end with a discussion of trade-offs and biomarkers.**
**To support this restructuring, we inverted the order of the figures (Fig. 4 is now Fig. 2 and inversely).**

• If the manuscript is reframed this way, revise title/abstract/Introduction accordingly (and avoid presenting the work primarily as a general "landscape transcriptomics proof-of-concept").
**Answer: We modified the title, abstract, introduction, and discussion to match the new narrative framing of the results. We really hope that the reviewer will like this new narrative.**

Optional but would strengthen: Briefly position this within the broader urban ecology/urban evolution trade-off framing (resource subsidies vs suboptimal conditions/chronic stress), as it aligns strongly with the story the manuscript develops.
**Answer: Thank you for suggesting a reframing of the manuscript and anchoring in broader urban ecology concepts, as well as suggesting key reviews and studies to support this. We tried to reinforce the ecological conceptual framing of the paper through the hypothesis statement (L 71-76): "*We hypothesised that juvenile and adult C. cyanea integrate multiple and interacting environmental factors into coordinated shifts in gene expression, and that these can reveal physiological trade-offs involved in urban life that remain invisible to classical ecological monitoring. Specifically, we predicted that human activities would lead to a signature of chronic stress and altered energy metabolism, a pattern reported in terrestrial urban wildlife*.", as well as in the discussion (L 354-356) "*This suggests that fish survival in urban zones depends on a trade-off; increased food availability in urban zones potentially attracts fish and partially buffers physiological stress.",* (L 356-358) *"similar patterns have been reported in other landscape transcriptomic studies: in two bird species, immune response was upregulated and coupled with a lipid-rich diet in urban zones",* (L 361-364) "*The footprint of food intake on stress markers was also noted in the Elbe estuary, where juvenile fish immune response was hypothesised to be suppressed under starvation*. This *potential commonality in transcriptome signatures in response to human activities and energy availability must be explored in more ecosystems and taxa*."**

*2. Establish a clear "urban stress" gradient and use the radar framework as the bridge*

Essential to address for publication: A major reason the story feels fragmented is not the lack of analyses, but the order in which they are presented. The reader does not see an early, quantitative bridge between: (i) environmental/urban variables, (ii) fish status (feeding condition / stress proxies), and (iii) genome-wide expression patterns. Your radar-plot framework is well-suited to serve as this bridge, but it currently appears later than would be optimal for guiding interpretation. In particular, the radar-plot summary is a strong candidate for this integrative 'bridge' and would be most effective if introduced immediately after the site-gradient definition.

**Answer: We have done our best to revise the manuscript following these suggestions.**

Actionable suggestions:

• Define the environmental gradient transparently first. Add a multivariate characterisation/ordination of sites based on the full measured environmental dataset (not only land cover), and use this to define a defensible "urban/environmental" axis (even if composite).

**Answer: We defined the urban/natural axis on the basis of the percentage of natural zones near each site. This is because Okinawa has very permeable karstic geology: land use closest to the site is bound to have a very strong effect on marine organisms. Using the % natural zone near each site is an effective, objective, and robust index of how humans are influencing local zones.**

**The definition of a more complex urban/environmental axis would be dependent on what environmental parameters we include and how we define them (for instance, grouping live and dead coral together or separately, considering all the macroalgae species even though *C. cyanea* may favour some specific ones, applying the same weight to different metrics) and this carries a risk of subjectivity. The % of natural zone near each site is a much more neutral (not to say objective) measure. In addition, we did not measure all the parameters affecting the fish in the field, and for instance we did not quantify chemical pollutants, light pollution, boat sound pollution, etc. mostly for logistical reasons.**

**In summary, we framed the study around the percentage of human activities near each site as it can be expected to capture a broad bundle of stressors influencing local marine organisms. Indeed, there is no single way to define a defensible urban/environmental axis; some variables hold more or less influence over fish gene expression (cf. Fig. 4); we might not have captured the full set of relevant parameters.**

**Importantly, the full environmental dataset is provided in the supplementary materials for transparency and so that researchers interested in reusing our data for specific questions have access to a comprehensive characterisation of each site.**

**We have added an NMDS grouping the abiotic parameters in addition to the two NDMS for the juvenile and adult fish populations (Extended Data Fig. 10).**

• Test confounding explicitly. Report whether land cover covaries with temperature, salinity, chlorophyll-α, sampling time, etc., and show how this is addressed (model structure, sensitivity/supplementary analyses).

**Answer: We added a correlation matrix between land cover and the environmental parameters in the supplementary materials (Extended Data Fig. 10), which is a complement to Fig. 1b and the Supplementary Tables. This correlation matrix highlights that there are no significant correlations between the percentage of natural zones near each site and any of the other measured parameters. We have indicated this in the manuscript (L 681-684): "*Non-Metric Multidimensional Scaling was used to assess the relationship between the sites based on the environmental parameters and on the juvenile and adult fish communities (Extended Data Fig. 10). There were no significant***

***correlations between the percentage of natural zones near each site and any of the other considered environmental parameters."***

**In addition, we evaluated the response of metrics to environmental parameters in three instances: to inform what influences feeding status (Fig. 3), gene module behaviour across the samples (Extended Data Fig. 7), or individual genes (Fig. 4).**

**For Fig. 3 and Extended Data Fig. 7, we used model dredging to choose the combination of parameters with smallest AIC criterion value.**

**For Fig. 4, we ran linear/quadratic models between the expression of each gene and each environmental / intrinsic fish parameter separately, then summarised the results to extract which genes respond to which parameter. We selected this approach because it best captured the patterns that we noticed in our dataset upon close inspection, notably quadratic effects on temperature on gene expression.**
**We recognise that this methodology does not account for more complex interactive effects between parameters. There is currently no single solution to detecting complex non-linear effects of parameters on whole transcriptomes, which is an issue recognised in the literature. We now mention this in the introduction (L 87-92)** "***An obstacle for ecological applications of transcriptomics is that statistical modelling of complex and context-dependent effects and interactions between factors requires careful design, can suffer from overparameterisation, and risks downplaying regulatory interactions between genes themselves. Although analytical frameworks exist, few simultaneously account for non-linear multi-stressor gradients and maintain sufficient transparency required to identify meaningful patterns in field transcriptomes."***

**We chose to use Dromics as it can detect both linear and quadratic relationships, which are common patterns in ecotoxicology. We clarified the Methods and added a sensitivity analysis (L 804-821) "*Using DRomics 2.6-225, the genes responding linearly or quadratically to various environmental factors (percentage of natural zones near each site, temperature, sampling time, salinity, chlorophyll-α) and to size (for juvenile fish) were obtained at a significance threshold of adjusted p < 0.01. We grouped together genes that have a linear and quadratic response to focus on which genes are sensitive to given parameters in a monotonic or biphasic manner. This methodology is based on comparing the expression level of the genes in the samples to each parameter separately and thus does not account for complex interactive effects between parameters. However, current transcriptome-wide modelling frameworks do not currently support exhaustive model selection across multiple interacting factors, and thus we elected to use Dromics to test for linear and quadratic effects with a robust statistical framework. We focused on the genes that responded uniquely to the percentage of natural zones near each site, and to no other parameter used in DESeq2 1.36.0 (female vs. male effect in adult livers) or Dromics 2.6-2 (all other parameters). For these genes, as an additional layer of verification, sensitivity analysis was performed by gradually excluding 1 to 6 sites from the models and determining whether the genes were still retrieved and thus showed robust association with the percentage of natural zones near each site. Two types of genes emerged, those with robust association (low frequency of absence in models) and those dependent on the inclusion of particular sites (refer to Extended Data Fig. 11)".***

• Move the "bridge" analysis earlier and make it a signpost. Introduce fish "status" metrics (feeding-status proxy; stress-related signatures) and the radar-plot summaries early in the Results, mapped onto the environmental gradient. This will help the reader understand the proposed "urbanisation → feeding/stress axis" before the more detailed gene-by-gene or pathway-level transcriptomic interpretation.

**Answer: As suggested, we inverted the order of the results to match the suggested structure. Note however that as feeding status is an important aspect of the radar plots, we kept it in the radars (formerly Figure 4, now now Figure 2), although feeding status is only formally defined in Figure 3. This is indicated in the legend of Fig.2 (L 203-204), "*Sample-specific feeding status (derived from Fig. 3) is indicated for context.*"**

- Would strengthen (but consistent with your current approach): Keep the radar framework, but state explicitly what it is doing: a hypothesis-guided diagnostic summary to integrate signals (rather than a purely exploratory visual). Then refer back to it consistently when interpreting subsequent transcriptomic patterns (e.g., "sites with high feeding index but elevated stress radar scores show…").

**Answer: As suggested, we added direct mentions to the radar plot as a diagnostic summary (L 159-162) "*We visualised these values on radar plots (Fig. 2). Unlike heatmaps or PCAs, these simultaneously display the status of individual fish in terms of distinct biological processes, enabling the detection of co-occurring mechanisms and physiological trade-offs across sites and life stages*." and refer to the radars more explicitly when discussing patterns (L 260-261) "*well-fed juveniles showed altered appetite-related radar signatures, reflecting a downregulation of satiety-signalling gene expression".***

*3. Feeding experiment interpretation: tighten what is demonstrated vs hypothesised Essential to address for publication: The feeding experiment is valuable and is a key strength of the paper. However, some manuscript language implies stronger mechanistic specificity (e.g., "fast-food / high-lipid" diets) than is directly shown by the feeding manipulation and field data.*

Actionable suggestions:

• Clearly state what the feeding experiment manipulates (fed vs unfed; duration; diet type), and what it can and cannot infer about "excess," "diet quality," or "high-lipid" urban diets.

**Answer: We now state explicitly "*we conducted fasting-feeding experiments*" (L 208-209). In the methods section, we provide details about the type of diet and duration of the experiment, for instance for juveniles (L 727-730): *"Two treatments were used: fasted and fed (control). In one tank, the fish were fed standard amounts of food for three days (dry pellets twice per day, ASIN number: B0BFJ9MY8P); in the other tank, the fish were not fed for three days and a mesh filter was used on the water inlet to remove particles and plankton*."**

• If the "fast-food/high-lipid" framing is retained, provide supporting evidence/analyses (diet composition proxies, stomach contents, lipid metrics, stable isotopes, prey-field differences, or clearly justified external datasets). Otherwise, temper wording so the conclusion is framed as a resource subsidy (higher feeding status) paired with stress/inflammation signatures, without specifying diet quality/composition.

**Answer: As suggested, we tempered our wording to focus on the elevated feeding status and similarity of the urban fish with the well-fed experimental fish without overreaching about the mechanistic specificity of the experiment and removed the "high lipid" mentions throughout the manuscript.**

*4. Definition of "human activities" signal and confounding with other drivers*

Essential to address for publication: The manuscript reports "genes uniquely linked to human activities," yet temperature, salinity, sampling time, and other variables strongly influence gene expression. Without a transparent operational definition and clear handling of collinearity, attribution to "human activities" is difficult to evaluate.

Actionable suggestions:

• Provide a clear operational definition for "uniquely linked to human activities" (model form, thresholds, multiple testing, and assignment rules).
**Answer: The reviewer is entirely correct. We tempered our wording in the results (L 320): "*associated to human activities and no other tested parameter*".**

**To fully take this comment into account, we also added a clause referring to the approach used to conclude that the genes can be used as markers of stress (L 328-331): "*Because these genes did not respond linearly or quadratically to other environmental or organismal factors, they may represent diagnostic markers of human-related stress, conveying mechanisms used by fish to survive in urban environments*".**

**The models are described in the methods section – we used the package Dromics 2-6.2 (Larras et al. 2018) to derive linear and quadratic models between each the expression of each gene and individual parameters, one at a time. The false discovery rate threshold was adjusted p < 0.01. We compared the list of genes responding to each parameter to see which genes "uniquely" responded to one of the tested parameters, which genes responded to two parameters, and which genes responded to more than two, as presented on Fig. 4.**

• Show diagnostics: correlation among predictors, sensitivity to influential sites (not only temperature extremes), and robustness to alternative definitions of the "urban" axis.
**Answer: We added a correlation matrix between land cover and the environmental parameters in the supplementary materials, which is a complement to Fig. 1b and the Supplementary Tables. We evaluated the response of metrics to environmental parameters in three instances: to inform what influences feeding status (Fig. 3), gene module behaviour across the samples (Extended Data Fig. 7), or individual genes (Fig. 4).**
**For Fig. 3 and Extended Data Fig. 7, we used model dredging to choose the combination of parameters with smallest AIC criterion value.**
**For Fig. 4, we ran linear/quadratic models between the expression of each gene and each environmental / intrinsic fish parameter separately, then summarised the results to extract which genes respond to which parameter. We selected this approach because it best captured the patterns that we noticed in our dataset upon close inspection, notably quadratic effects on temperature on gene expression.**

**We did not test robustness to alternative definitions of the urban axis, as we elected to streamline the definition to the percentage of natural zones near each site to avoid subjectivity. Indeed, the definition of alternative urban/environmental axis would be dependent on what environmental parameters are included and how they are defined. However, to test the robustness of our approach and since the focus of the paper is the link between urbanisation and fish gene expression, for both juveniles and adults, we conducted a sensitivity analyses centred around the genes responding to the natural-urban gradient by running 100 models excluding 1 to 6 sites from the linear/quadratic models. This revealed two categories of genes: those whose association depended on the inclusion of particular sites (particularly the most natural and most urban ones) and a robust subset (approximately half of all genes for the juveniles with 227 genes and adult livers with 291 genes) that was repeatedly recovered. As the heatmaps resulting from this additional testing and associated GO terms were very similar to those retrieved with the full list of genes, we kept the analysis with the full list of genes in the main text (Fig. 4) and added a supplementary figure (Extended Data Fig. 11).**
**This is described in the methods (L 814-822) "*We focused on the genes that responded uniquely to the percentage of natural zones near each site, and to no other parameter used in DESeq2 1.36. 0***

***(female vs. male effect in adult livers) or Dromics 2.6-2 (all other parameters). For these genes, as an additional layer of verification, sensitivity analysis was performed by gradually excluding 1 to 6 sites from the models and determining whether the genes were still retrieved and thus showed robust association with the percentage of natural zones near each site. Two categories of genes emerged, those with robust association (low frequency of absence in models) and those dependent on the inclusion of particular sites (refer to Extended Data Fig. 11)*.".**

• Ensure site-level environmental structure is carried consistently into downstream analyses and figure colouring/grouping.

**Answer: We have carefully checked that all figures use the same environmental structure logic and colouring/grouping pattern based on a gradient from natural (green) to urban (red).**

*5. Methods clarity and reproducibility: strengthen key details*

Essential to address for publication: Several methodological components are central to the conclusions but are currently underspecified or potentially confusing.

Actionable suggestions:

• Land cover linkage: Clarify what "land cover within 1 km" means for a marine sampling point (buffer definition, spatial workflow/software), and justify why this is the appropriate terrestrial linkage (vs catchment-based influence/discharge).

**Answer: To take into accound the reviewer's comment, we updated the methods section as follows (L 671-680): "*Land use near each site was quantified from a publicly available dataset released in January 2023. As Okinawa's coastal zones are characterised by karstic geology with high permeability and rapid subterranean water flow, coastal zones are influenced by localised groundwater and surface outfluxes rather than solely by surface-topography defined watersheds. Therefore, we derived human presence from the percentage of natural zones within 1km radial buffer around the coordinates of each field site. Spatial analysis was performed in QGIS by extracting the percentage of pixels belonging to natural zones (evergreen broad-leaved forests, evergreen needle-leaved forests, mangrove forests) and excluding pixels corresponding to water bodies. This proximity-based metric served as a proxy for the degree of exposure to human activities occurring near each site.*"**

• Use of additional field variables: Community/substrate/turbidity/fish density appear collected but their role in the main models is unclear. If they were tested and excluded, say so (and why). If not used, streamline or clarify their purpose.

**Answer: We agree that a transparent variable selection process is essential. We collected fish community and substrate data to provide an exhaustive ecological context for each site. However, in our downstream analyses relating gene expression to environmental conditions, we prioritised parameters with established physiological links to gene regulation (temperature, salinity, circadian rhythm) and those related to our primary hypotheses (natural-urban gradient, feeding status). As juvenile C. cyanea are planktivorous and adult C. cyanea rely on plankton and algal turf, we selected chlorophyll-a, a proxy of primary productivity. Since turbidity and chlorophyll-a were highly correlated (Extended Data Fig. 10a) we retained chlorophyll-a to avoid model overparameterisation. Lastly, we tested for relationships between C. cyanea density, community composition, and our derived feeding status from Fig. 3. Since we did not find significant associations, we did not include fish community composition data in statistical models. For transparency, we provide community composition and substrate data in the supplementary materials (Table S1, S2, Extended Data Fig. 10) and on FigShare (10.6084/m9.figshare.27966972). Researchers interested in reusing our data for specific questions will therefore have access to a comprehensive characterisation of each site.**

• DE workflow clarity: Explicitly describe the DESeq2 contrast strategy (pairwise site contrasts vs reference-site strategy), whether “p < 0.01” refers to adjusted p-values (FDR) or raw p-values, and how the many tests are interpreted/aggregated.

**Answer: We updated the methods section to state (L 799-806): “*Using DESeq2 1.36.0, the number of differentially expressed genes in pairwise site comparisons was estimated at the threshold of adjusted p < 0.01 and absolute fold change of 1.5 (logFC of 0.58). Since 18 sites were considered, 153 pairwise comparisons were performed. The genes responding to sex in the adult liver samples were derived with the same thresholds. Using DRomics 2.6-2, the genes responding linearly or quadratically to various environmental factors (percentage of natural zones near each site, temperature, sampling time, salinity, chlorophyll-α) as well as size for juvenile fish were obtained at a significance threshold of adjusted p < 0.01.*”.**

• Reference genome vs transcriptome ambiguity: Specify exactly what “reference transcriptome” is (*de novo* assembly vs transcript models from the genome), including accession/version and how the Kallisto index was built. Because Kallisto yields transcript-level estimates, explicitly describe how transcript abundances were aggregated to gene-level counts for DESeq2 (e.g., tximport and rules for isoforms). Also, describing Kallisto as “aligned” is misleading; revise terminology.

**Answer: As the focus of this study is gene regulation rather than alternative splicing and isoform switching, the reference transcriptome used in this study consists of a gene-level transcript set with the longest isoforms for each gene, as described in Gairin et al. (2024) Gigabyte, 10.46471/gigabyte.144 This reference was derived from transcripts annotated on the reference genome (it is not a *de novo* transcriptome assembly). A kallisto index was built from the longest isoform reference transcriptome. The pseudoalignment was thus performed at the gene level and no aggregation of transcript-level abundances (e.g., via tximport) was required prior to the analyses based on DESeq2. We revised the terminology and methodology to address this comment (L 772-777): “*To reduce transcript-level redundancy and focus on gene regulatory shifts, we used a reference transcriptome filtered to only keep the longest isoform for each of the 28,173 annotated genes. This reference transcriptome is available on FigShare (doi: 10.6084/m9.figshare.27143571)18,19. Gene-level abundance was quantified using Kallisto 0.46.218. The number and percentage of pseudo-aligned reads were […].*”**

• QC reporting: Provide explicit RNA integrity and QC summaries (TapeStation DIN/RIN-equivalent values, read depth per sample, mapping rates already given, and any filtering thresholds).

**Answer: We added the quality check information about the RNA samples in Tables S7, 8, 9. We now provide the number of reads per sample in the materials and methods (L 764-767): “*The average number of paired-end reads was 72.2 ± 6.5 million for the juvenile and adult field samples, 68.0 ± 6.0 million for feeding experiment juvenile whole-body samples, and 80.9 ± 9.3 million for feeding experiment adult liver samples.*”.**

**We also provide more details about trimming parameters (L 771-772) “*ILLUMINACLIP:TruSeq3-PE-2.fa:2:30:10:8:keepBothReads LEADING:3 TRAILING:3 SLIDINGWINDOW:4:15 MINLEN:36 HEADCROP:11*”.**

**Finally we also the number and percentage of pseudo-aligned reads (L 776-779): “*The number and percentage of pseudo-aligned reads were 48.0 ± 4.9 (mean ± sd) million reads and 68.1 ± 3.8 % for field juvenile whole-body samples, 58.5 ± 7.1 million reads and 81.7 ± 7.1 % for field adult liver samples, 39.6 ± 4.0 million reads and 58.5 ± 3.5 % for feeding experiment juvenile whole-body samples, and 58.6 ± 6.7 million reads and 73.3 ± 1.7 % for feeding experiment adult liver samples.*”.**

Optional but would strengthen: The population genetics (RNA-seq SNP calling; DAPC) is potentially useful to rule out population structure confounding, but it currently feels disconnected. Add a short statement in Results/Discussion with the take-home message and point to the Extended Data figure, or move it fully to Supplementary Methods.

**Answer: We now address this point in the first paragraph of the result (L 111-112): "*We first checked that the transcriptomic profiles were not confounded by genomic variation; variant calling retrieved no significant population structure across Okinawa (Extended Data Fig. 1).*"**

Detailed Comments (by line)

*Framing, literature, and hypothesis clarity*

(Lines 26–27) The citations motivating multi-stressor frameworks (e.g., Gissi; Pirotta) feel somewhat dated given how quickly this area is moving. Please consider updating the Introduction with more recent syntheses and/or field-oriented multi-stressor frameworks (e.g., https://doi.org/10.1002/ece3.71959 and additional recent literature), so the manuscript is anchored in the current state of the field.

**Answer: Thank you for the literature suggestions; we have reinforced the conceptual framing of our introduction with recent syntheses and reviews/studies based on tailored analytical frameworks for multi-stressor approaches for *ecology and global change biology (L 77-80): "In the field, gene expression is influenced by numerous factors, and these can have non-linear effects; sampling across stressor gradients rather than categorical factors is increasingly advocated for[29–31]. However, explicitly accounting for all factors affecting organisms would lead to a "combinatorial explosion" [9,32]. To address this, we adopted a semi-randomised fractional and multifactorial sampling strategy[32–34]".***

**We also addressed limitations in current analytical frameworks for landscape transcriptomics (L 87-92): "*An obstacle for ecological applications of transcriptomics is that statistical modelling of complex and context-dependent effects and interactions between factors requires careful design[29,35,36] (references include the suggested work by Madge Pimentel et al. 2025, https://doi.org/10.1002/ece3.71959), can suffer from overparameterisation[37,38], and risks downplaying regulatory interactions between genes themselves[39,40]."***

(Lines 34–36; 40–42) The Introduction/Discussion would benefit from explicitly framing the work within an urban ecology / urban evolution trade-off perspective (organisms exploiting urban resource subsidies while tolerating suboptimal conditions and chronic stress). This framing is closely aligned with your later results/interpretation (feeding status vs stress trade-offs). A useful entry point is the "urban evolution" framework paper (DOI: 10.1126/science.aam8327).

**Answer: Thank you for the suggestion to reframe our manuscript around this urban ecology perspective. Accordingly, we have centred the revised introduction around urban concepts, starting from the first sentence (L 41): "*Human activities are altering natural habitats, creating novel types of ecosystems exposed to complex combinations of environmental stressors[1,2] (references include Johnson and Munshi-South, 2017, 10.1126/science.aam8327*).", and extensively modified the framing of the objectives (L 69): "*To explore how urbanised environments reconfigure gene expression in coastal fish*", hypotheses (L 71-76): "*We hypothesised that juvenile and adult C. cyanea integrate multiple and interacting environmental factors into coordinated shifts in gene expression, and that these can reveal physiological trade-offs involved in urban life that remain invisible to classical ecological monitoring. Specifically, we predicted that human activities would lead to a signature of chronic stress and altered energy metabolism, a pattern reported in terrestrial urban wildlife*". We also modified the structure of the results to fit this suggestion.**

**Our discussion was also reinforced to discuss the trade-offs involved in life near urban zones, particularly the first paragraph (L 351-364) "Landscape transcriptomics provides a molecular-based perspective on how anthropogenic pressures shape organismal status and ecological outcomes in urbanised coastal ecosystems. We observed that adult fish from sites with higher human presence were well-fed, with elevated immune and inflammatory responses (Fig. 3,4b). This suggests that fish survival in urban zones depends on a trade-off; increased food availability potentially attracts fish and partially buffers physiological stress. Similar patterns have been reported in other field-based transcriptomic studies: in two bird species, immune response was upregulated and coupled with a**

**lipid-rich diet in urban zones. In juvenile C. cyanea, there was a tight coupling between nutrition and stress response, notably in terms of immunity and xenobiotic metabolism, and this link persisted even in individuals from relatively pristine zones (Fig. 2, Extended Data Fig. 7). The footprint of food intake on stress markers was also noted in the Elbe estuary, where juvenile fish immune response was hypothesised to be suppressed under starvation. This potential commonality in transcriptome signatures in response to human activities and energy availability should be explored in more ecosystems and taxa."**

(Line 48) When discussing the conceptual shift from single-stressor laboratory exposures to multi-stressor real-world contexts (and how omics can help bridge these), it would strengthen the narrative to cite foundational perspectives alongside omics-era discussion. Suggested references: 10.1016/j.aquatox.2003.11.011; 10.1016/j.cbd.2016.06.010.

**Answer: Thank you for this advice. We have reinforced our review of the advantages of complex multi-stressor contexts combined with transcriptomics approaches with the suggested perspectives and references (L 55-66) *"With the rise of high-throughput sequencing, transcriptomics has become a central approach for ecological and ecotoxicological studies in the laboratory but also in the field* (references include suggested works by Snape et al. 2004, 10.1016/j.aquatox.2003.11.011, Martyniuk and Simmons 2016, 10.1016/j.cbd.2016.06.010)*. […] Unlike targeted approaches, transcriptomics provides unbiased and hypothesis-free gene expression data. Importantly, changes in gene expression can precede physiological effects, offering mechanistic insight and serving as early warning signals for later-stage outcomes such as disease, reduced fitness, or population declines. Moreover, by focusing on conserved genes and pathways, transcriptomic responses can be compared across taxa, facilitating the development of generalisable frameworks for understanding organismal resilience under anthropogenic stress. Building on these advantages, landscape transcriptomics—the study of how genome-wide expression patterns reflect dynamic, landscape-scale environmental drivers—has emerged as a comprehensive approach to monitor environmental change in wild organisms."***

(Lines 62–63) Please strengthen contextualisation of environmental/ecological transcriptomics in fish, as this is central to the manuscript's positioning. Suggested citations: 10.1242/jeb.148833; 10.1139/facets-2022-0177; 10.1016/j.cbpb.2021.110629.

**Answer: Thank you for this advice. We have reinforced the positioning of fish environmental and ecological studies with omics approaches (L 52-55): "*In aquatic ecosystems, fish are a common study taxon*[12,13] *(including the suggested references, Connon et al. 2018 <u>10.1242/jeb.148833</u> and Jeffries et al. 2017, <u>10.1016/j.cbpb.2021.110629</u>) and their physiology is well-characterised in model species such as zebrafish*[14–17]*. Extensive genomic resources and homology across teleost genes enable detailed molecular studies even in non-model species*[18,19]*."***

(Line 64) The aim is clear here, but the manuscript's main "take-home" shifts later. Please clarify early what the primary hypothesis is (monitoring proof-of-concept vs urban resource–stress trade-off) and keep the narrative consistent throughout.

**Answer: We sharpened the hypotheses in the introduction (L 71-76) "*We hypothesised that juvenile and adult C. cyanea integrate multiple and interacting environmental factors into coordinated shifts in gene expression, and that these can reveal physiological trade-offs involved in urban life that remain invisible to classical ecological monitoring. Specifically, we predicted that human activities would lead to a signature of chronic stress and altered energy metabolism, a pattern reported in terrestrial urban wildlife*" and followed a tighter narrative throughout the revised manuscript.**

(Line 92) A few statements read like results or interpretations appearing in the Introduction. Please keep the Introduction focused on background, rationale, and hypotheses, and relocate result-like statements to the Results/Discussion sections.

**Answer: We revised the introduction to ensure it focuses on hypotheses and objectives and relocated result-like statements to the appropriate sections of the manuscript. See the revised manuscript as the text has been heavily modified.**

*Differential expression strategy and definition of "urban signal"*

(Lines 97–98) The hypothesis is currently presented very broadly ("gene expression shifts along the gradient"), but later the manuscript interprets patterns in specific functional directions stress/inflammation; food/energetics). Consider sharpening the hypothesis and predictions up front, ideally grounded in prior environmental transcriptomics studies across anthropogenic environmental gradients (e.g., 10.1016/j.scitotenv.2024.173656; 10.1016/j.envpol.2024.124400). Relatedly, clarify how any targeted gene sets/panels are defined to avoid the perception of post hoc selection (clearly distinguish hypothesis-driven panels from exploratory outputs).

**Answer: We sharpened the hypotheses in the introduction (L 71-76) "*We hypothesised that juvenile and adult C. cyanea integrate multiple and interacting environmental factors into coordinated shifts in gene expression, and that these can reveal physiological trade-offs involved in urban life that remain invisible to classical ecological monitoring. Specifically, we predicted that human activities would lead to a signature of chronic stress and altered energy metabolism, a pattern reported in terrestrial urban wildlife."* and reinforced the discussion.**

**We added context to ground the choice of the gene sets (L 154-156): "*for energy metabolism, neurotransmission, endocrine regulation, and stress response, which are processes expected to vary in C. cyanea in response to human activities*[22,23]".**

(Lines 104–108) The DESeq2 field-comparison approach needs a clearer explanation. Please explicitly state: (i) the design formula used, (ii) whether all pairwise site contrasts were performed or a reference-site framework was used, (iii) how many contrasts/tests were run, (iv) whether the stated threshold ("$p < 0.01$") is adjusted p-values (FDR) or raw p-values, and (v) how these outputs are used downstream (e.g., descriptive "transcriptomic distance," frequent-DE genes, site grouping). As written, the workflow is difficult to reproduce and the large DEG counts may be misinterpreted without these details.

**Answer: As requested, we updated the methods section to state (L 800-807): "*Using DESeq2 1.36.0, the number of differentially expressed genes in pairwise site comparisons was estimated with thresholds of adjusted p < 0.01 and absolute fold change of 1.5 (logFC of 0.58). Since 18 sites were considered, 153 pairwise comparisons were performed. The genes responding to sex in the adult liver samples was derived using DESeq2 1.36.0 with the same significance thresholds. Using DRomics 2.6-2*[25]*, the genes responding linearly or quadratically to various environmental factors (percentage of natural zones near each site, temperature, sampling time, salinity, chlorophyll-α) and to size (for juvenile fish) were obtained at a significance threshold of adjusted p < 0.01.".* As there was no reference site, we state (L 799-800): "*As there are no reference or control conditions in the field, we focused on comparisons between each site.*"**

*Environmental gradient, confounding, and "human activities" attribution*

(Lines 136–138) Because temperature has a strong footprint, please explicitly address whether "urbanisation" covaries with temperature (and/or salinity, chlorophyll-α). If more urbanised sites are systematically warmer (etc.), show how this is handled analytically.

**Answer: We added a correlation matrix in the supplementary materials (Extended Data Fig. 10) to highlight that our sampling strategy covered a large number of combinations and allowed to avoid covariation between our main target variable, the natural gradient, and the other environmental parameters. In particular, the correlation coefficient between the % of natural zones and temperature is -0.16 ($p = 0.83$). We added the following precision in the methods (L 683-684): "*There were no significant correlations between the percentage of natural zones near each site and any of the other considered environmental parameters.*".**

(Lines 140–146) When identifying "425 genes (juveniles) / 585 genes (adult liver) uniquely linked to human activities," please define "uniquely linked" operationally (models used, thresholds, handling of collinearity, and assignment rule). Without this, the strength of the attribution to human influence is hard to evaluate.

**Answer: As discussed above, we tempered our wording in the results (L 319-320): "*associated to human activities and no other tested parameter*". For Fig. 4, we ran linear/quadratic models between the expression of each gene and each environmental / intrinsic fish parameter separately, then summarised the results to extract which genes respond to which parameter. We selected this approach because it best captured the patterns that we noticed in our dataset upon close inspection, notably quadratic effects on temperature on gene expression. We recognise that this methodology does not account for more complex interactive effects between parameters. There is currently no single solution to detecting complex non-linear effects of parameters on whole transcriptomes, which is an issue recognised in the literature. We now mention this in the introduction (L 87-92) "*An obstacle for ecological applications of transcriptomics is that statistical modelling of complex and context-dependent effects and interactions between factors requires careful design, may suffer from overparameterisation, and risks downplaying regulatory interactions between genes themselves. Although analytical frameworks exist, few simultaneously account for non-linear multi-stressor gradients and maintain sufficient transparency to identify meaningful patterns in field transcriptomes.*"**

**To test the robustness of our approach, we conducted a sensitivity analysis centred around the genes responding to the natural-urban gradient by running 100 models excluding 1 to 6 sites from the linear/quadratic models. This revealed two categories of genes: those whose association depended on the inclusion of particular sites (particularly the most natural and most urban ones) and a robust subset (approximately half of all genes for the juveniles with 227 genes and adult livers with 291 genes) that was repeatedly recovered. As the heatmaps resulting from this additional testing and associated GO terms were very similar to those retrieved with the full list of genes, we kept the analysis with the full list of genes in the main text (Fig. 4) and added a supplementary figure (Extended Data Fig. 11). We describe our approach (L 816-822), "*For these genes, as an additional layer of verification, sensitivity analysis was performed by gradually excluding 1 to 6 sites from the models and determining whether the genes were still retrieved and thus showed robust association with the percentage of natural zones near each site. Two categories of genes emerged, those with robust association (low frequency of absence in models) and those dependent on the inclusion of particular sites (refer to Extended Data Fig. 11).*" and now refer to this analysis in our results (L 319-321) "*We identified 425 genes in juvenile whole-body and 585 genes in adult livers associated to human activities and no other tested parameter (list of genes provided on FigShare; for robustness testing, cf. Extended Data Fig. 11).*".**

(Lines 146–150) I strongly recommend adding an environmental ordination/clustering (PCA/NMDS or similar) using the full environmental dataset. This would allow the reader to see whether "urbanisation" is a composite of correlated stressors and would better justify downstream grouping/colouring/interpretation.

**Answer: We present environmental characteristics in the Supplementary Materials (Tables S1, 2, Extended Data Fig. 10). Note however that the urbanisation axis is not a composite mix of parameters that we measured; instead, we defined the urbanisation/human presence axis as an index of nearby land use as described in the methods (L 671-680) "*Land use near each site was quantified from a publicly available dataset released in January 2023. As Okinawa's coastal zones are characterised by karstic geology with high permeability and rapid subterranean water flow, coastal zones are influenced by localised groundwater and surface outfluxes rather than solely by surface-topography defined watersheds. Therefore, we derived human presence from the*"**

***percentage of natural zones within 1km radial buffer around the coordinates of each field site. Spatial analysis was performed in QGIS by extracting the percentage of pixels belonging to natural zones (evergreen broad-leaved forests, evergreen needle-leaved forests, mangrove forests) and excluding pixels corresponding to water bodies. This proximity-based metric served as a proxy for the degree of exposure to human activities near each site.".***

*Integration of field + experiment: strengthen the causal story*

(Lines 174–178) The manuscript would benefit from explicitly stating (as hypothesis/prediction) whether the central aim is (i) "gene expression varies along land cover/human footprint," or (ii) "urban habitats provide increased food resources and fish trade off energetic gains against chronic stress." The current integration can read as a post hoc linkage between two strong datasets.

**Answer: As discussed above and to follow the reviewer suggestion, we reframed the manuscript and explicitly worded hypotheses and results to coherently connect the two datasets (see response to various comments from the reviewer).**

(Lines 190–196) The strongest contribution, in my view, is the integration of field transcriptomes with a controlled feeding experiment to interpret field patterns in terms of energetic state and stress trade-offs. I recommend foregrounding this contribution throughout (title, objectives, Results structure), rather than presenting the work primarily as a general landscape transcriptomics demonstration.

**Answer: We fully agree and we now emphasise the integration of experimental and field data and its value for data interpretation through a reframing of the introduction, results, and discussion, and we modified the title to reflect the focus on urban-related ecological trade-offs.**

(Lines 197–205) The metatranscriptomic diet inference is only presented for juveniles. Please clarify early why this is restricted to juveniles (whole-body data), and strengthen interpretation by addressing: (i) do these diet signatures vary systematically along the urban gradient, and (ii) what is the plausible mechanism of food increase in urban settings (direct subsidies vs nutrient-driven shifts in prey biomass/community composition)? Even if adults cannot be analysed similarly, avoid overgeneralising.

**Answer: The meta-transcriptomic diet could be derived for juveniles as we sequenced the whole body (including gut content). For adults, we only sequenced the livers and thus cannot perform the same analysis; it is possible that nutrient outfluxes promote greater primary productivity and presence of key species that adult damselfish target for feeding.**

**Note that we did not detect a significant influence of human presence on juvenile feeding status. This is now clearly indicated in the manuscript (L 223): "*no significant association to the measured environmental parameters"* nor on the non-host read results/diet signature.**

**As a related note, please find an improved version (Fig. 3e) of the diet-derivation for the juveniles *C. cyanea* based on presenting only the taxa corresponding to zooplankton, phytoplankton, metazoans, plants, and invertebrates, and removing microscopic bacteria and fungi that were present in the whole-body transcriptomes but do not correspond to food sources for juvenile damselfish.**

(Lines 235–239) This analysis appears to be the "missing link" that could make the manuscript's logic much clearer. I suggest introducing (or moving) this earlier, immediately after defining the environmental/urban gradient—so fish condition/feeding status and stress signals are mapped onto the gradient before the untargeted transcriptome interpretation. This re-organisation would substantially improve readability and strengthen the causal narrative.

**Answer: We modified the order of the results to match suggestions from the reviewer.**

Discussion scope

(Lines 323–327) The Discussion expands into pollution/toxicant framing, but chemical pollution is not directly measured or presented (as currently visible to the reader). I suggest narrowing the Discussion to what is supported by the measured variables and analyses, unless external pollution datasets are integrated explicitly and transparently. A tighter scope focused on the urban food–stress trade-off would increase clarity and impact.
**Answer: We narrowed the discussion to a tighter framing with the trade-off between food and physiological stress in urban environments.**

(Lines 340–346) The manuscript currently reads as two separate experiments connected by interpretation rather than by a single pre-stated hypothesis. A major revision should aim to unify the narrative: define the urban gradient, demonstrate its relationship with fish status/stress, interpret transcriptome patterns along that axis, and use the feeding experiment to anchor mechanistic interpretation.
**Answer: We unified the manuscript by modifying the presentation of the results to match the order suggested by the reviewer. We now explicitly use the feeding experiment to anchor mechanistic interpretation of patterns across biological processes.**

*Methods transparency and reproducibility (additional specifics)*
(Lines 506–507) Fish community data are mentioned but their analytical role is unclear. Please specify how community metrics were used (or, if not used, clarify why they were collected and where they are reported).
**Answer: We collected fish community to provide ecological context for each site. However, in our downstream analyses relating gene expression to environmental conditions, we prioritised parameters with established physiological links to gene regulation (temperature, salinity, circadian rhythm) and those related to our primary hypotheses (natural-urban gradient, feeding status).**

**We tested for relationships between C. cyanea density, community composition, and our derived feeding status from Fig. 3. Since we did not find significant associations, we did not include fish community composition data in statistical models. For transparency, we provide community composition data in the supplementary materials (Table S1, Extended Data Fig. 10) and on FigShare (10.6084/m9.figshare.27966972). Researchers interested in reusing our data for specific questions will therefore have access to a comprehensive characterisation of each site.**

(Lines 517–518) Land-cover methodology needs to be reproducible and conceptually appropriate for marine sampling points. Please clarify software/workflow, spatial unit (buffer), and how terrestrial land cover is linked to each marine site (shoreline buffer vs catchment influence).
**Answer: We apologize for the lack of clarity on this point. To address this comment, we updated the methods section as follows (L 671-680): "*Land use near each site was quantified from a publicly available dataset released in January 2023. As Okinawa's coastal zones are characterised by karstic geology with high permeability and rapid subterranean water flow, coastal zones are influenced by localised groundwater and surface outfluxes rather than solely by surface-topography defined watersheds. Therefore, we derived human presence from the percentage of natural zones within 1km radial buffer around the coordinates of each field site. Spatial analysis was performed in QGIS10 by extracting the percentage of pixels belonging to natural zones (evergreen broad-leaved forests, evergreen needle-leaved forests, mangrove forests) and excluding pixels corresponding to water bodies. This proximity-based metric served as a proxy for the degree of exposure to human activities near each site.*"**

(Lines 519–521) Several measured variables (substrate, fish density, turbidity, diversity metrics) do not appear in the key models. Clarify whether they were tested/excluded and provide a concise overview of variable selection.

**Answer: We agree that a transparent variable selection process is essential. We collected fish community and substrate data to provide an exhaustive ecological context for each site. However, in our downstream analyses relating gene expression to environmental conditions, we prioritised parameters with established physiological links to gene regulation (temperature, salinity, circadian rhythm) and those related to our primary hypotheses (natural-urban gradient, feeding status). As juvenile *C. cyanea* are planktivorous and adult *C. cyanea* rely on plankton and algal turf, we selected chlorophyll-a, a proxy of primary productivity. Since turbidity and chlorophyll-a were highly correlated (Extended Data Fig. 10a) we retained chlorophyll-a to avoid model overparameterisation.**

**Lastly, we tested for relationships between *C. cyanea* density, community composition, and our derived feeding status from Fig. 3. Since we did not find significant associations, we did not include fish community composition data in statistical models. For transparency, we provide community composition and substrate data in the supplementary materials (Table S1, S2, Extended Data Fig. 10) and on FigShare (10.6084/m9.figshare.27966972). Researchers interested in reusing our data for specific questions will therefore have access to a comprehensive characterisation of each site.**

(Line 573) The feeding experiment contrasts "fed (normal food amounts)" vs "fasted," which supports interpretation in terms of feeding status/energetic state. It does not directly test "excess" or "low-quality/high-lipid" urban diets unless diet composition/quality is measured. Please separate what is demonstrated from what is hypothesised, and temper wording accordingly if needed.
**Answer. The reviewer is correct. We tempered our wording to focus on the elevated feeding status and similarity of the urban fish with the well-fed experimental fish without overreaching about the mechanistic specificity of the experiment, and now refer to the experiment as fasting-feeding consistently, as well as to "*feeding status*" (*e.g.,* L 217, L 218, L 225, L 389) and "*predicted to be well fed*" (L 228).**

(Lines 596–598) Please report standard RNA quality metrics (e.g., TapeStation DIN/RIN-equivalent values) and key QC statistics, not only general statements about checks performed.
**Answer: We now provide RNA quality metrics in Tables S7,8,9 and reinforced the materials and methods section.**

(Line 599) Please clarify what "reference transcriptome" was used, provide accession/version, and describe gene-level aggregation from Kallisto outputs for DESeq2. Also replace "aligned" with accurate terminology for Kallisto-based quantification.
**Answer. As requested, we revised the methodology (L 772-776) and provided references to the reference transcriptome: "*To reduce transcript-level redundancy and focus on gene regulatory shifts, we used a reference transcriptome filtered to only keep the longest isoform for each of the 28,173 annotated genes. This reference transcriptome is available on FigShare (doi: 10.6084/m9.figshare.27143571). Gene-level abundance was quantified using Kallisto 0.46.218. The number and percentage of pseudo-aligned reads […]*".**

(Lines 678–690) If population structure analyses are included to rule out confounding, state the purpose and take-home message in the main text (with a pointer to the Extended Data figure), or move to Supplementary Methods to avoid disconnection.
**Answer: We now address this point in the first paragraph of the result (L 111-113): "*We first checked that the transcriptomic profiles were not confounded by genomic variation—variant calling retrieved no significant population structure across Okinawa (Extended Data Fig. 1).*"**

*Conclusion and Recommendation*
This manuscript presents a substantial field and experimental effort, and the authors have assembled a rich, multi-level dataset with clear potential for a strong contribution. In particular, the combination

of landscape transcriptomics with controlled feeding experiments is innovative, and the effort to translate multi-gene signals into interpretable summaries is promising.
That said, the manuscript currently requires major revision before it can be considered for publication. The central limitation is not the amount of data, but the conceptual focus and integration: the field and experimental components read as partially disconnected, and several key interpretations (especially regarding “human activities” attribution and diet-quality mechanisms) are not yet supported with sufficient analytical transparency and framing. Strengthening the definition of the environmental/urban gradient, clarifying how “human-activity–linked” signals are derived, tightening claims to what is directly demonstrated, and reorganising around a single hypothesis-driven narrative would substantially improve interpretability.
I therefore recommend major revisions. With a clearer hypothesis, a coherent analytical flow (environmental gradient → fish status/stress → transcriptome patterns → experimental anchoring), and strengthened methods transparency, I believe this study could become a compelling and well-supported manuscript.
**Answer: We deeply thank the reviewer for the amount of work done on our manuscript. We have done our best to take into account all the comments and in particular the suggestions about the conceptual focus and integration. We really hope that the reviewer will find this new version fully satisfactory.**

Disclaimer: I used a large language model (LLM) to assist in drafting and editing this peer-review report (improving structure, clarity, and wording). The scientific assessment, interpretation, and final recommendations are my own, and I take full responsibility for the content of this report.

**Reviewer #1**

I previously reviewed this manuscript and I think the authors did an excellent job responding to my suggestions as well as those of the other reviewer. The dramatic reorganization they did in response to the other reviewer really improves the overall impact of the paper. It feels more hypothesis-driven rather than technique driven now.
**Answer: We thank the reviewer for highlighting the improvement of the manuscript following the first round of reviews and agree that the reorganisation strengthens the narrative and improves clarity and impact.**

I have some feedback, but my purpose is to give constructive criticism. I want to emphasize that overall I think this is an important contribution that goes well beyond prior work on urbanization effects by leveraging transcriptomics in creative ways as a window into physiological effects of anthropogenic stressors on wild free-living fish across a large geographic range (along the coast of the island of Okinawa). Not only is it an important test case, but the results have important implications for our understanding of anthropogenic effects on animals in the short and possibly long term. The paper is very well written (often challenging with this kind of data), leverages a combination of field-based, experimental, and literature mining data to form conclusions. The results highlight the complex trade-offs that come with environments closer to human disturbance – fish appear well fed but suffer other stress effects according to their transcriptomes.
**Answer: We thank the reviewer for recognising the quality of our work and the value of our transcriptomic-based approach for addressing ecological questions.**

Some main points of feedback:

1. With the new reorganization, the radar plots come early, and while I do think this is the right place for them, the fact that these are presented as qualitative analyses gave the paper a bit of a story-telling vibe to me. I think the authors do a good job of highlighting certain sites as examples to underline their points, but I wonder if there is a quantitative method that could be developed to judge these comparisons. For example, perhaps something borrowed from geometric morphometrics to ask how similar two radar plots are in shape? Presumably a null distribution would be a circle traversing the middle of the radar plot… could that fact be leveraged somehow?
   **Answer: Thank you for this suggestion. We quantified the similarity between the radar plot profiles of each site using clustering based on Euclidean distances between the radar plot coordinates across biological processes for each site (Extended Data Fig. 5a,b), allowing quantitative assessment of site similarity. We now refer to this in the main text (Lines 166-168): *"Juveniles from sites with comparable water temperature and urbanisation level (A, B, L as well as N, R; see Fig. 2, Extended Data Fig. 5) exhibited closely aligned profiles, as did adults from the most natural sites (H, I) and most urbanised sites (E, G, M)".* We added the corresponding mention in the Methods, Lines 894-896: "*Similarity between sites was derived from Euclidean distances between radar plot coordinates (Extended Data Fig. 5).".***

2. The authors did a good job in their revision of better explaining how these radar plots are constructed. This is a bit of a quibble, but when they say that "The first eigenvector of the covariance matrix of the average site coordinates was used to define a main axis of site-wise variance across the retained PC dimensions." this is the same as the PC1 from a PCA of the average site coordinates.
   **Answer: Thank you for this clarification. We now state (Lines 880-882): *"The first principal component of the average site coordinates across the retained PC dimensions was used to define a projection axis representing the main axis of site-wise variance".***

3. I am still not a fan of the WGCNA analysis. It is done on a very small subset of curated genes and the authors of WGCNA have made it very clear back when they developed the software that they do not think that's an appropriate use. Also I'm not sure what it gains. From what I can see in the Extended Figure 7, the modules are difficult to interpret (span multiple categories). I would suggest instead taking each category of genes, taking the PC1 of the expression of genes within each and using those PC1 scores instead of scores from the eigengenes of constructed modules. Further exploration of the overlap in expression between these categories of genes could be done, for example with a correlation matrix.

   **Answer: We thank the reviewer for this suggestion and agree that applying WGCNA to a curated gene set may limit interpretability. We have therefore replaced Extended Data Fig. 7 (now Extended Data Fig. 5c,d). The previous analysis based on co-expressed gene modules has now been replaced with an equivalent approach using genes grouped *a priori* by biological processes. These include established stress-related categories (xenobiotics, heat shock proteins, immune response, corticoids, thyroid hormones, neurotransmission, reactive oxygen species). This revised approach captures variation in expression within defined biological processes, as suggested by the reviewer. The overall conclusion remains unchanged: we observe a limited natural-urban signature in canonical stress-related pathways.**

   **We describe the methods used to produce the new Extended Data Fig. 5c,d in Lines 892-894, "*The relationship between radar plot coordinates, environmental parameters, and intrinsic fish characteristics (standard length for juveniles, sex for adults) was tested using linear-mixed effect models with site as random effect.".***

4. The Discussion still feels a little lopsided, emphasizing landscape transcriptomics more relative to the implications of the results themselves.

   **Answer: Thank you for this important point. We have revised the Discussion to emphasise the biological discussion of the results. We modified the opening paragraph (Lines 354-370) to more directly highlight our findings: "*While urbanisation effects on wild organisms at the population level are increasingly reported, integrated responses to complex multi-stressor conditions remain largely unresolved. Here, landscape transcriptomics provides a molecular-based perspective on how anthropogenic pressures shape organismal status in urbanised coastal ecosystems. We observed that adult coral reef fish from Okinawa living on coastal sites close to human activities were well-fed, but showed elevated immune and inflammatory responses (Fig. 3,4b). This suggests that fish survival in urban zones depends on a trade-off: despite local stressors, increased food availability potentially attracts fish and could fuel stress responses[50,51]. Similar patterns have been reported in other field-based transcriptomic studies: in two bird species, immune response was upregulated and coupled with a lipid-rich diet in urban zones[26,27]. In contrast, immune and stress responses in juvenile fish were coupled with nutrition rather than with human presence, a tight link that persisted even in individuals from relatively pristine zones (Fig. 2, Extended Data Fig. 5). This footprint of food intake on stress markers was also noted in the Elbe estuary, where juvenile fish immune response at the transcriptomic level was hypothesised to be suppressed under starvation[52]. Crosstalk between nutritional status and stress mechanisms may thus be conserved across taxa and life stages, with energy availability influencing responses to anthropogenic effects."***

   **We also revised the third paragraph (Lines 382-393) to more explicitly connect our results to their local ecological implication before expanding to the broader value of landscape transcriptomics: "*The gene-environment atlas derived from***

***C. cyanea's landscape transcriptomes in Okinawa highlighted complex relationships and interacting effects between environmental drivers, and identified genes exclusively associated with human presence in juvenile whole-body and adult liver transcriptomes. These constitute reference resources to select genes for targeted molecular assays while excluding non-target effects. Adopting this methodology across field contexts would be of high value, as transcriptomic signatures provide higher specificity and sensitivity than single traits or markers under multi-stressor conditions and can be used to disentangle compensatory effects from irreversible consequences. Our results support a transition in environmental monitoring from discrete measurements of local parameters and organismal traits to the precise detection of organismal stress, even at sublethal and low intensity levels, and prediction of longer-term outcomes at the population level."*** **We believe that these changes provide a more balanced discussion.**

More minor points (line number refer to the version without tracked changes):
Line 110: Why is variant calling just with juveniles, and is it based on the transcriptomes or other genomic data?
**Answer: Variant calling was performed using the transcriptomes of juveniles only, as whole-body transcriptomic reads covered a larger fraction of the genome (on average 23%) compared to adult liver reads (10%). This is described in the Methods, Lines 895-899, *"For juvenile whole-body transcriptomes, in which a higher number of genes was expressed than in the adult liver samples and thus a larger proportion of the genome was captured, the raw sequencing reads were mapped to the reference genome using 2-pass mapping using STAR 2.7.9a[30] followed by data cleaning with Picard (AddOrReplaceReadGroups, MarkDuplicates).".***

Figure 1f: I was curious what the null distribution for this would look like – it could be derived through simulation by permuting the existing data.
**Answer: Thank you for this suggestion. We now present Extended Data Fig. 3 with the null distribution derived from 1,000 permutations preserving the number of DEGs per pairwise comparison while randomising gene assignment. We also performed Kolmogorov-Smirnov tests to compare the observed distribution from Fig. 1f with the null distribution, which showed that genes are detected across more pairwise site comparisons than expected by chance. We added the following to the results (Lines 119-125): "*Juveniles exhibited broad, frequently differentially expressed genes, with 2,410 genes retrieved in >25% of the pairwise site comparisons (significantly exceeding null distribution, Kolmogorov-Smirnov test, D > 0.53, p < 10-15, permutation p < 0.001; Fig. 1f, Extended Data Fig. 3). While adults showed more site-specific, fine-tuned patterns in their liver, a key organ for energy metabolism and detoxification, 251 genes were nonetheless retrieved in >25% comparisons (also significantly higher than null distribution, D > 0.52, p < 10-15, permutation p < 0.001).*".**

Line 256: I think I'd still refer to them as "radar plots" instead of "radars"
**Answer: The term "radar plots" is now used consistently throughout the manuscript.**

Line 280: Be careful with words like "modulate" that imply a cause-and-effect in a paper where the data are correlational.
**Answer: Thank you for this comment. We modified the sentence (Lines 284-285): from "*Numerous genes involved in endocrine signalling were modulated by temperature in both juvenile and adult transcriptomes*" to "*Numerous genes involved in endocrine signalling showed changes in expression levels correlated with temperature in both juvenile and adult transcriptomes*".**

Line 318: Change "to" to "with"

**Answer: Thank you for identifying this grammar issue.**

Line 352: Insert "but" before "with"

**Answer: We corrected the sentence at Lines 358-360: from "*We observed that adult fish from sites with higher human presence were well-fed, with elevated immune and inflammatory responses (Fig. 3,4b)*" to "*We observed that adult coral reef fish from Okinawa living on coastal sites close to human activities were well-fed, but showed elevated immune and inflammatory responses (Fig. 3,4b)*".**

Line 354: Why say "partially buffers"? I thought these fish were well-fed but more stressed?

**Answer: We revised the sentence to remove "*partially buffers*" and now state that increased food availability could "*fuel stress responses*" (Line 361).**

Line 392: There is a study in bumble bees that might be appropriate to cite here: DOI: 10.1111/mec.17626

**Answer: Thank you for this suggestion. We have added this study in two relevant sections (reference 60), improving the support for cross-comparability between experimental and field settings (Lines 400-407): "*To enhance data interpretation, the footprint of different stressors from additional laboratory assays[60] (e.g., for temperature, salinity, prevalent chemical compounds) […] Cross-comparability of transcriptomes from various studies and experimental contexts, accompanied by robust analytical methods, promises to uncover novel effects between factors[29,60].***

Figure 2 legend: "The first eigenvector of the average coordinates from each site" should say "first eigenvector of the covariance matrix of the average coordinates" according to your methods.

**Answer: We modified the terminology to "first principal component" instead of first eigenvector of the covariance matrix", as suggested by the reviewer, and accordingly updated Fig. 2's legend to: *"The first principal component of the average coordinates from each site across the retained PC dimensions was used to define a main axis of site-wise variance, summarising the dominant gradient of site-wise variation for each biological process"* (Lines 195-198).**

Supplemental Table 10: I missed what this was for?

**Answer: This table compares radar plot coordinates for each biological process with mean scaled expression for each gene within each biological process. In most cases, there is a strong positive correlation, indicating that higher radar coordinates, closer to the edge, correspond to higher overall gene expression within the biological process.**

Extended Data Figure 2: Minor typo – there are 8 PCs for adult liver and 6 for juvenile bodies, not 6 for each. Also can you clarify – is each column a single linear model?

**Answer: We corrected this typo and clarified the legend of Extended Data Fig. 2: *"For juvenile whole-body (a) and adult liver (b), bar charts of the percentage of contribution to the variance of the top PC dimensions, corresponding to over 50% of the total variance. Tile plots presenting the significance of the linear relationship between various environmental parameters and PC coordinates. For each PC, a linear mixed effect model with site as a random effect and variables selected from preliminary linear models on the basis of lowest BIC value using regsubset (leaps package in R).".***

In conclusion, I commend the authors on an excellent study and a very well written paper that was an enjoyable and interesting read.

**Answer: We thank the reviewer for their positive assessment and constructive feedback, which have helped to improve the manuscript.**

Reviewer #2 (Remarks to the Author):

I thank the authors for the thorough and thoughtful revision. The manuscript has improved substantially, and I appreciate the clear effort invested in addressing the conceptual, analytical, and methodological points raised in the first review.

In particular, the revised version is now much more coherent and hypothesis-driven. The manuscript successfully clarifies the central narrative and improves the integration between the landscape transcriptomics component and the feeding experiment. The strengthened definition and visualisation of the environmental/urban gradient, together with clearer treatment of potential confounding among environmental drivers, greatly improve interpretability and make the main conclusions more robust. I also commend the improved methodological transparency in the omics workflow and land-cover linkage, which strengthens reproducibility and reader confidence.

Overall, the revision has addressed my major concerns, and the manuscript now reads as a mature, well-supported contribution. I congratulate the authors on the quality of the work and the care taken in the revision.

Recommendation: I support publication of this manuscript in its current form (acceptance).

**Answer: We sincerely thank the reviewer for their thorough evaluation and constructive comments over both rounds of review. We are pleased that the revision has improved the coherence and interpretability of the manuscript and are grateful for the reviewer's support for publication of the manuscript in its current form.**

**Response to reviewers**

Extended Data Fig.2: Could you further clarify whether all variables in the table were included in models for each PC? In other words, was the same model run for each PC? Or could blanks mean that the variable was not included because of BIC criteria?

***Grey hashes were added to signify that the variable was not included while white tiles indicate that the variable was tested but did not reach the significance criterion of $p < 0.01$.***

Extended Data Fig. 3: The y-axis of c&d is log-scale, but the plotted variable is still the number of DEGs. So the y-axis title should still be simply "Number of DEGs". Also in the title of the caption, should "with" be "within"?

***The axis title was corrected. The caption title was updated to be clearer: "Distribution of DEG occurrence across pairwise site comparisons compared with a null expectation".***

Extended Data Fig. 5: Why is site M missing from part b? Also, a similar question as before; were all variables in this chart included or do blanks mean they weren't?

***M was missing due to a figure editing error during the formatting. It has been put back now (the submitted figure had two "J" sites for the adults). Grey shading was added to signify that the variable was not included while white tiles indicate that the variable was tested but did not reach the significance criterion of $p < 0.01$.***

Extended Data Fig. 12: Missing text on x axis.

***The missing text was put back in place.***
